# A Trinuclear Gadolinium Cluster with a Three-Center One-Electron Bond and an *S* = 11 Ground State

**DOI:** 10.1021/jacs.3c00182

**Published:** 2023-04-17

**Authors:** K. Randall McClain, Hyunchul Kwon, Khetpakorn Chakarawet, Rizwan Nabi, Jon G. C. Kragskow, Nicholas F. Chilton, R. David Britt, Jeffrey R. Long, Benjamin G. Harvey

**Affiliations:** † Naval Air Warfare Center, Weapons Division, Research Department, Chemistry Division, US Navy, China Lake, California 93555, United States; ‡ Department of Chemistry, University of California, Berkeley, Berkeley, California 94720, United States; § Department of Chemical and Biomolecular Engineering, University of California, Berkeley, Berkeley, California 94720, United States; ∥ Department of Chemistry, University of California, Davis, Davis, California 95616, United States; ⊥ Department of Chemistry, The University of Manchester, Manchester M13 9PL, U.K.; # Materials Sciences Division, Lawrence Berkeley National Laboratory, Berkeley, California 94720, United States

## Abstract

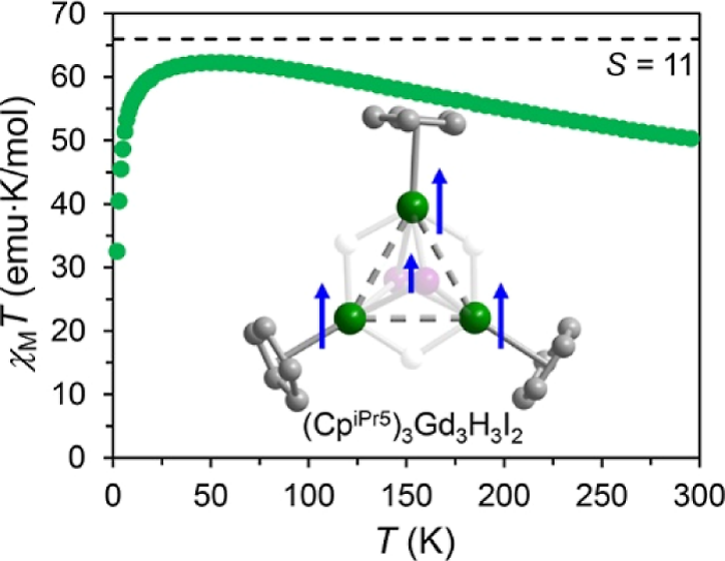

The recent discovery of metal–metal bonding and valence delocalization in the dilanthanide complexes (Cp^iPr5^)_2_Ln_2_I_3_ (Cp^iPr5^ = pentaisopropylcyclopentadienyl; Ln = Y, Gd, Tb, Dy) opened up the prospect of harnessing the 4f^*n*^5d_z^2^_^1^ electron configurations of non-traditional divalent lanthanide ions to access molecules with novel bonding motifs and magnetism. Here, we report the trinuclear mixed-valence clusters (Cp^iPr5^)_3_Ln_3_H_3_I_2_ (**1-Ln**, Ln = Y, Gd), which were synthesized via potassium graphite reduction of the trivalent clusters (Cp^iPr5^)_3_Ln_3_H_3_I_3_. Structural, computational, and spectroscopic analyses support valence delocalization in **1-Ln** resulting from a three-center, one-electron σ bond formed from the 4d_z^2^_ and 5d_z^2^_ orbitals on Y and Gd, respectively. Dc magnetic susceptibility data obtained for **1-Gd** reveal that valence delocalization engenders strong parallel alignment of the σ-bonding electron and the 4f electrons of each gadolinium center to afford a high-spin ground state of *S* = 11. Notably, this represents the first clear instance of metal–metal bonding in a molecular trilanthanide complex, and the large spin–spin exchange constant of *J* = 168(1) cm^–1^ determined for **1-Gd** is only the second largest coupling constant characterized to date for a molecular lanthanide compound.

## Introduction

The study of metal–metal orbital interactions in molecular compounds has significantly expanded the fundamental understanding of the nature of chemical bonding and is driving progress in areas such as organometallic catalysis^1^ and molecular magnetism.^2,3^ For example, transition metal complexes featuring direct metal–metal orbital overlap can exhibit peculiar electronic structures that are unrealizable in traditional metal clusters, as a result of strong electronic coupling between multiple paramagnetic centers.^4−8^ Metal–metal bonding in the hexanuclear iron cluster [(MeC(CH_2_NPh-*o*-NH)_3_)_2_Fe_6_(py)_2_]^2–^ (py = pyridine) results in an *S* = 11 ground state that remains almost exclusively populated up to ambient temperature.^9^ Likewise, the tetranuclear complex [Co_4_(NP^*t*^Bu_3_)_4_]^+^ exhibits an *S* = ^9^/_2_ ground state arising from direct metal–metal orbital overlap and a spin relaxation barrier of *U*_eff_ = 87 cm^–1^, the largest reported to date for a transition metal cluster single-molecule magnet.^10^

While examples of metal–metal bonding are well-known for complexes featuring transition metals with diffuse d orbitals,^11^ the realization of direct metal–metal interactions in multinuclear lanthanide complexes has traditionally been considered an insurmountable challenge, owing to the radially contracted nature of the core-like 4f valence orbitals. However, the discovery of non-traditional divalent lanthanides with 4f^*n*^5d_z^2^_^1^ electron configurations^12−15^ introduced the possibility of accessing new metal–metal bonding motifs in lanthanide compounds. Exploiting this unique electronic structure, we recently reported the mixed-valence dilanthanide complexes (Cp^iPr5^)_2_Ln_2_I_3_ (Cp^iPr5^ = pentaisopropylcyclopentadienyl; Ln = Y, Gd, Tb, Dy), which exhibit unprecedented valence delocalization as a result of a singly occupied σ-bonding molecular orbital of 5d_z^2^_ parentage (4d_z^2^_ in the case of Y). Here, strong spin–spin coupling between the 4f electrons on each paramagnetic Ln center and the σ electron spin results in a well-isolated, high-spin ground state of *S* = ^15^/_2_ for the Gd congener and large spin–orbit coupled ground states of *M*_*J*_ = ±31/2 ( term) and *M*_J_ = ±25/2 (^14^I_25/2_ term) for the Dy and Tb congeners, respectively. Notably, as a result of their unique electronic structures, the latter two complexes also exhibit massive coercive fields that exceed previously reported values for any molecule or molecule-based material.

Building on this precedent, we were interested in accessing higher-nuclearity lanthanide clusters exhibiting metal–metal bonding. Given the large single ion magnetic moments and magnetic anisotropy of the lanthanide ions, such clusters could potentially support very large spin ground states that persist at or near room temperature. We hypothesized that by using a smaller bridging ligand such as hydride, it might be possible to access higher-nuclearity clusters while also decreasing the average Ln···Ln separation, due to the smaller ionic radius of hydride (1.40 Å) versus iodide (2.20 Å).^16^ Herein, we report the synthesis and characterization of the trinuclear mixed-valence clusters (Cp^iPr5^)_3_Ln_3_H_3_I_2_ (**1-Ln**, Ln = Y, Gd), which are obtained via reduction of the corresponding homovalent precursors (Cp^iPr5^)_3_Ln_3_H_3_I_3_. Structural, computational, and spectroscopic analyses of **1-Ln** support the presence of a three-center one-electron σ bond of d_z^2^_ parentage. In the case of **1-Gd**, valence delocalization enforces a parallel alignment of the 4f electrons on all three lanthanide centers, giving rise to a well-isolated *S* = 11 ground state.

## Results and Discussion

### Synthesis and Structural Characterization

**Scheme 1:**
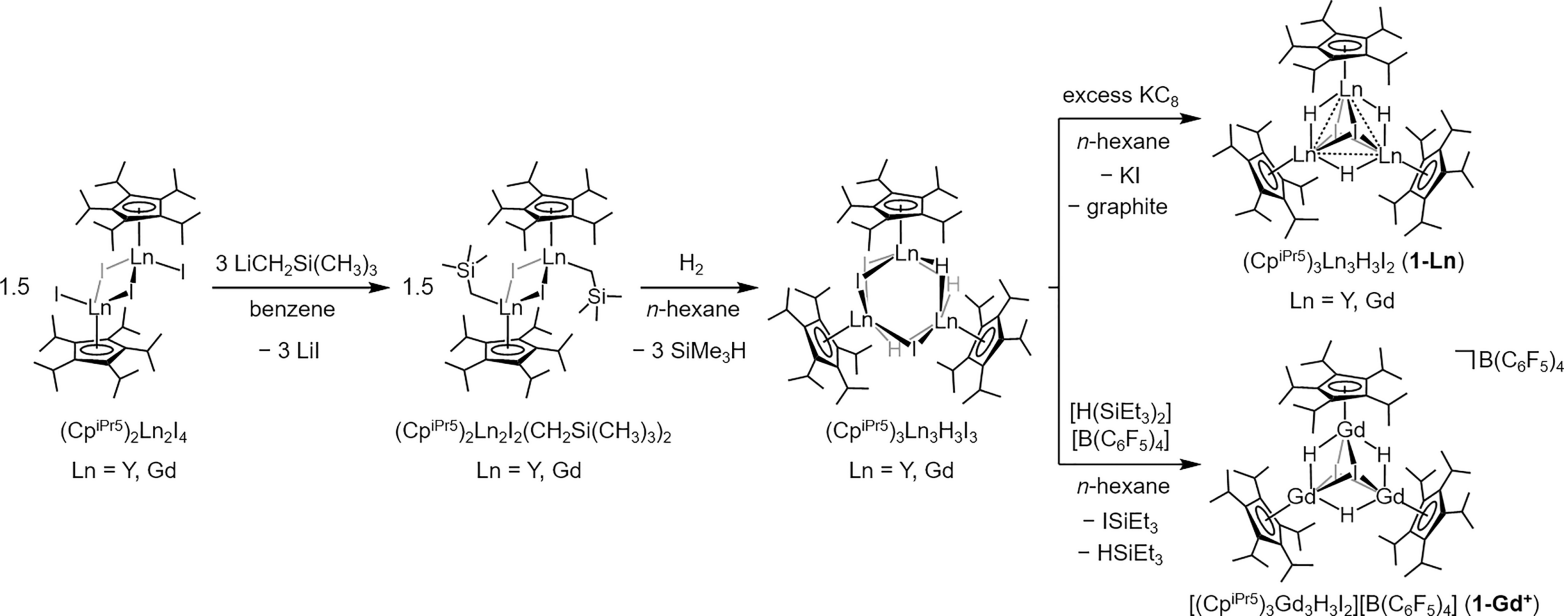
Synthetic Routes to the Mixed-Valent Trinuclear Clusters **1-Ln** (Ln = Y and Gd) and **1-Gd**^**+**^

We previously isolated the mixed-valence dinuclear lanthanide complexes (Cp^iPr5^)_2_Ln_2_I_3_ via reduction of the trivalent precursors (Cp^iPr5^)_2_Ln_2_I_4_.^3^ Toward isolation of the proposed trinuclear cluster, we carried out sequential alkylation and protonolysis reactions to selectively substitute iodide for hydride (Scheme 1; see Section 1 of the Supporting Information). In brief, alkylation of (Cp^iPr5^)_2_Ln_2_I_4_ (Ln = Y, Gd) with two equiv of LiCH_2_Si(CH_3_)_3_ in benzene resulted in the formation of the mixed iodide/alkyl complexes (Cp^iPr5^)_2_Ln_2_(CH_2_Si(CH_3_)_3_)_2_I_2_ (Figures S12 and S13), which were subjected to protonolysis under an H_2_ atmosphere in *n*-hexane. Subsequent liberation of tetramethylsilane and a change in the cluster nuclearity generated the trinuclear precursor complexes (Cp^iPr5^)_3_Ln_3_H_3_I_3_ (Figures S14 and S15). Chemical reduction of (Cp^iPr5^)_3_Ln_3_H_3_I_3_ with potassium graphite (KC_8_) in *n*-hexane and crystallization from *n*-hexane then afforded the mixed-valence trinuclear clusters (Cp^iPr5^)_3_Ln_3_H_3_I_2_ (**1-Ln**) as dark green (**1-Y**) or dark green-blue (**1-Gd**) crystals. The isolated compounds are indefinitely stable at room temperature under argon; however, they decompose rapidly at ambient temperature in air as indicated by a color change to a yellow or colorless solid. The analogous deuterated species (Cp^iPr5^)_3_Y_3_D_3_I_2_ (**1-Y**_**D**_) was prepared in a similar manner with protonolysis conducted under a D_2_ atmosphere. In order to investigate the impact of the additional electron on the Ln_3_H_3_I_2_ core, the isostructural, homovalent complex salt [(Cp^iPr5^)_3_Gd_3_H_3_I_2_][B(C_6_F_5_)_4_] (**1-Gd**^**+**^) was also synthesized via iodide abstraction from (Cp^iPr5^)_3_Gd_3_H_3_I_3_ with [H(SiEt_3_)_2_][B(C_6_F_5_)_4_].

In **1-Ln** and **1-Gd**^**+**^, the presence of three bridging hydrides is supported by MALDI mass spectrometry, infrared spectroscopy (Figures S2–S4), and electron paramagnetic resonance (EPR) spectroscopy (see below). Further, the calculated infrared spectra and isotope shifts for **1-Y** and **1-Y**_**D**_ (Figure S45; see Section 8 of the Supporting Information) are in excellent agreement with the experiment, confirming the presence of hydrides.

**Figure 1:**
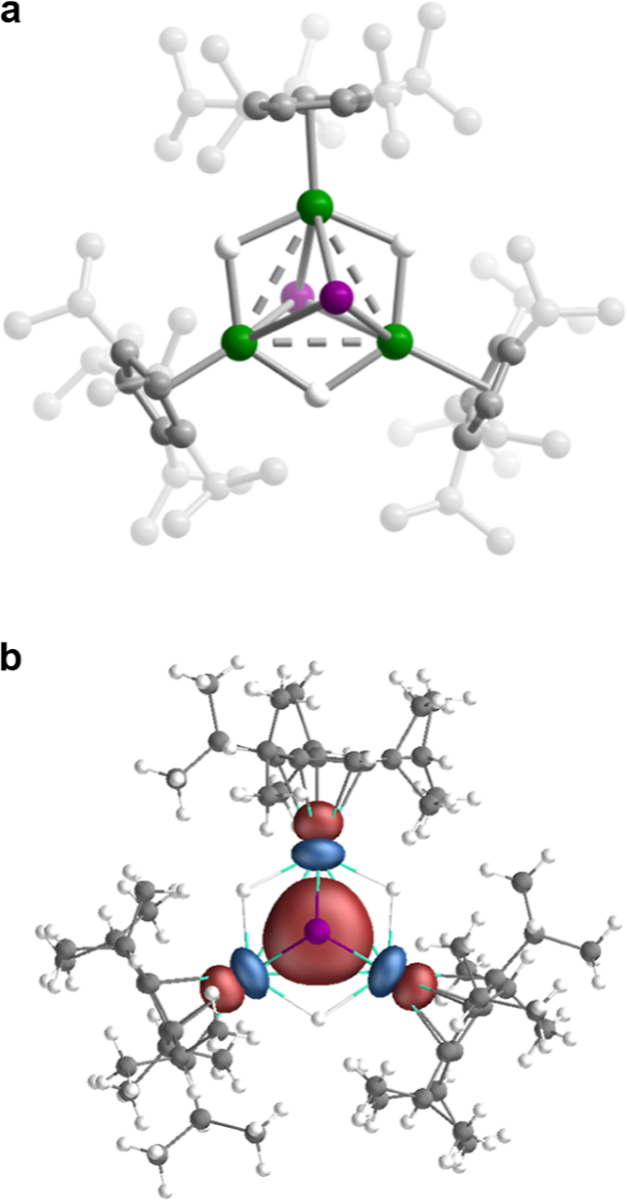
(a) Solid-state molecular structure of **1-Gd**. Green, gray, and white spheres represent Gd, C, and H atoms, respectively; H atoms on Cp^iPr5^ ligands and positional disorder are omitted for clarity; **1-Y** is isostructural to **1-Gd**. Ln···Ln distances (Å) for **1-Y**/**1-Gd**, respectively: 3.486(1), 3.519(1), and 3.520(1)/3.561(1), 3.595(1), and 3.602(1). (b) The σ-bonding SOMO of **1-Gd** as determined from DFT calculations (see the Supporting Information for details).

The solid-state structures of **1-Y**, **1-Gd**, and **1-Gd**^**+**^ were determined from the analysis of single-crystal X-ray diffraction data. The structures of **1-Y** and **1-Gd** feature an equilateral triangle of three metal centers with three edge-bridging μ-H anions lying within the plane of the triangle (Figure 1a). Two μ_3_-I anions are situated above and below the plane, and each lanthanide ion is capped by a radially extending Cp^iPr5^ ligand. The mean Ln···Ln distances of 3.508(1) and 3.586(1) Å, respectively, are ∼0.2 Å shorter than the Ln···Ln distances in (Cp^iPr5^)_2_Ln_2_I_3_ (Ln = Y, Gd; Table S6). The [Ln_3_H_3_I_2_]^3+^ core possesses a nearly ideal *D*_3*h*_ symmetry, supporting the possibility of valence delocalization within the clusters. Alternatively, an asymmetric structure would be expected for a valence-localized structure with two Ln^3+^ ions and one Ln^2+^ ion, due to the larger ionic radius of Ln^2+^.

The structure of **1-Gd**^**+**^ (Figure S19) features a [Gd_3_H_3_I_2_]^4+^ core that also closely approaches *D*_3*h*_ symmetry but with a mean Gd···Gd separation of 3.797(2) Å. Notably, this is significantly longer than the 3.586(1) Å in **1-Gd**, despite the smaller ionic radius of Gd^3+^, supporting the possibility of metal–metal bonding interactions in **1-Gd**. The mean Gd–Cp_centroid_ distance in **1-Gd**^**+**^ (2.339(1) Å) is shorter than that in **1-Gd** (2.406(1) Å), as expected given the average oxidation state of Gd^3+^ compared to Gd^2.67+^.

Complete active space self-consistent field (CASSCF) and DFT calculations carried out on **1-Y** and **1-Gd**, respectively, suggest a valence delocalized structure with a singly occupied molecular orbital (SOMO) that arises from σ bonding between the d_z^2^_ orbitals of the lanthanide ions (Figure 1b and Table S11). Of note, this bonding is reminiscent of that characterized in the recently discovered mixed-valence thorium cluster [(C_8_H_8_}_3_Th_3_Cl_6_]^2–^, which features a three-center, two-electron σ bond of 6d parentage.^14^ The σ-bonding SOMO of **1-Gd** is dominated by contributions from the Gd 5d_z__^2^_ orbitals (∼70%), with smaller contributions from I 5p and 5d orbitals (∼13%) and Gd 6p orbitals (∼10%). Thus, we characterize the metal–metal bonding interactions in **1-Gd** as occurring through a three-center, one-electron σ bond.

### EPR Spectroscopy

**Figure 2:**
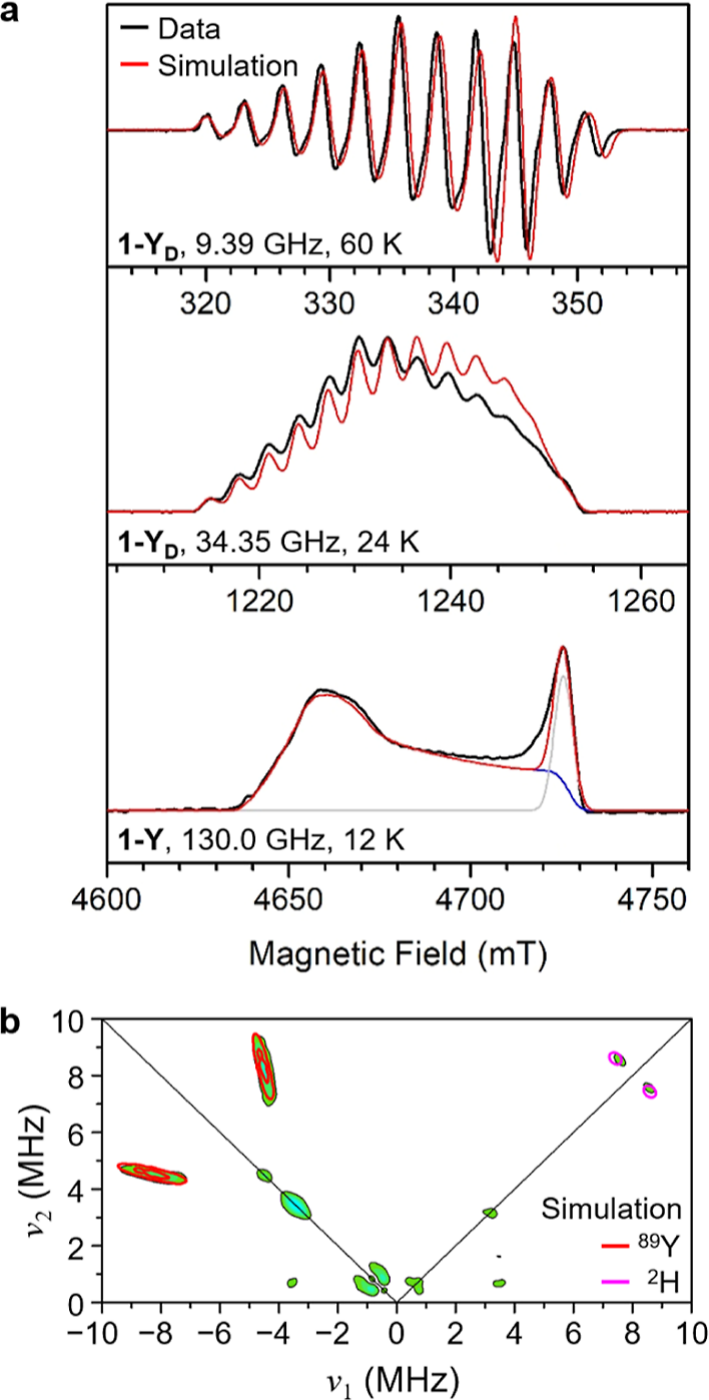
(a) CW X-band (upper) and field-sweep echo-detected Q-band (middle) EPR spectra collected for **1-Y**_**D**_ dissolved in methylcyclohexane, compared with a field-sweep echo-detected D-band EPR spectrum for **1-Y** in methylcyclohexane (lower). Experimental spectra are in black and simulated spectra are in red. In the D-band spectrum, the blue and grey lines represent, respectively, simulations of **1-Y** and a second paramagnetic species (∼15% of the total signal; see the Supporting Information S39b), which is ascribed to a small amount of sample decomposition. (b) Q-band HYSCORE spectrum for **1-Y**_**D**_ in methylcyclohexane collected at 20 K and 1.229 T. Green-cyan contours represent experimental data, while simulated ^89^Y and ^2^H hyperfine interactions are depicted as red and magenta contours, respectively.

Continuous-wave (CW) X-band EPR spectra collected for **1-Y** and **1-Y**_**D**_ in methylcyclohexane (Figure 2a, upper, and Figure S37) feature an isotropic signal split into 11 peaks via hyperfine interactions with two ^127^I nuclei (*I* = 5/2). Hyperfine splitting due to the ^1^H (or ^2^H) and ^89^Y nuclei are unresolved, indicating that these effects are relatively small and masked by the linewidth of the spectra (which display a peak-to-peak linewidth of 1.2 mT or 34 MHz). The field-sweep echo-detected Q-band spectrum of a frozen solution of **1-Y**_**D**_ (Figure 2a, middle) features a signal split into 13 peaks, suggesting that *g* anisotropy is partially resolved at this frequency (34 GHz). To further resolve *g* anisotropy, the EPR spectrum of **1-Y** was collected at D-band frequency (130 GHz) (Figure 2a, lower), revealing an axial signal with *g*_⊥_ = 1.9957 and *g*_∥_ = 1.965. The small deviation of the *g* factor from the free electron value (*g*_e_ = 2.0023) is consistent with the unpaired electron residing in a singly degenerate 4d_z^2^_ orbital, as observed previously for other cyclopentadienide complexes of Y.^3,17^

To resolve the anisotropy of the hyperfine interaction with ^127^I, we performed electron–electron double resonance-detected nuclear magnetic resonance (EDNMR) spectroscopy at D-band frequency (130 GHz). Similar to electron–nuclear double resonance (ENDOR) spectroscopy, EDNMR probes the transitions of a hyperfine-coupled nuclear spin; however, this technique is more sensitive than ENDOR spectroscopy (see Section 7.2 of the Supporting Information).^18^ Moreover, nuclear quadrupole couplings facilitate otherwise forbidden EDNMR transitions, such that hyperfine spectra of nuclei with quadrupole moments (such as ^127^I, *I* = 5/2) can be obtained with high sensitivity.^18^ We collected EDNMR spectra for **1-Y** at high magnetic fields ranging from 4.65 to 4.71 T (the field range of the field sweep D-band EPR spectrum in Figure 2a, lower), where the greater *g* factor separation enables *g* and *A* anisotropies to be resolved. The spectra exhibit multiple nuclear transitions (Figure S40) that could be simulated with hyperfine coupling to ^127^I (*A*_⊥_ = 87 MHz and *A*_∥_ = 20 MHz). Both the *A* and *g* tensors have axial symmetry (Figure S40). The CW and field-sweep spectra in Figure 2a could be well simulated using these *A*_⊥_ and *A*_∥_ values and the *g*_⊥_ and *g*_∥_ values extracted from the D-band spectrum of **1-Y** (Figure 2a).

As noted above, hyperfine splitting due to ^1^H (or ^2^H) and ^89^Y nuclei were not resolved in the CW spectra obtained for **1-Y** (**1-Y**_**D**_). To extract hyperfine interactions involving the bridging deuteride ligands, we collected the Q-band ^2^H ENDOR spectrum for **1-Y**_**D**_ (Figure S41). Simulation of this spectrum gave *A*_⊥_ (^2^H) = 1.5 MHz and *A*_∥_ (^2^H) = 0.7 MHz. Additionally, Q-band hyperfine sublevel correlation spectroscopy (HYSCORE) analysis of **1-Y**_**D**_ revealed a strong (*A* > ω_Y_, where ω_Y_ is the Larmor frequency of the ^89^Y nucleus) hyperfine coupling interaction between the σ electron and the ^89^Y nuclei, which could be well simulated with *A*_⊥_ (^89^Y) = 15.5 MHz and *A*_∥_ (^89^Y) = −1.2 MHz (Figure 2b). Of note, this is a rare example of an ^89^Y HYSCORE experiment; to the best of our knowledge, ^89^Y HYSCORE has previously only been used to analyze the compound (Cp^Me4H^)_2_Y(abpy) (abpy^•–^ = azobispyridine radical anion), which, in contrast to **1-Y**_**D**_, exhibits weak ^89^Y hyperfine coupling due to the unpaired electron residing on the organic radical ligand.^19^

Only one type of ^127^I, ^2^H, and ^89^Y nucleus was detected by the pulsed EPR experiments, which indicates that the unpaired electron in **1-Y** is fully delocalized over the trimetallic core. Table 1 summarizes the hyperfine tensors of ^89^Y, ^127^I, and ^2^H nuclei extracted from these experiments and decomposed into isotropic (*A*_iso_) and anisotropic or dipolar (*T*) components. Using atomic hyperfine parameters calculated by Morton and Preston,^20^ the electron density on each magnetic nucleus was calculated from the experimental hyperfine parameters (Table 1; see Section 7.3 of the Supporting Information for details). Note that the absolute signs of the hyperfine tensors could not be determined from the experiments. Instead, the signs reported in Table 1 were chosen such that the % d (^89^Y) is positive, as the majority of the spin density originates from d orbitals on the Y atoms; *T* (^2^H) is positive because it arises solely from the through-space dipolar interaction. The sign of *A* (^127^I) was chosen to coincide with that determined from our DFT calculations (see Table S12).

**Table 1:** Hyperfine Parameters (in MHz) and Experimentally Derived Electron Densities on the Atomic Orbitals (%) of Each Nucleus, Obtained from Simulations of Q-Band ^89^Y HYSCORE (**1-Y**_**D**_), D-Band EDNMR (**1-Y**), Field-Sweep Echo-Detected D-Band EPR (**1-Y**), and Q-Band ^2^H ENDOR (**1-Y**_**D**_) Spectra^a^

nuclei	^89^Y	^127^I	^2^H
*A* exp.	[15.5, 15.5, −1.2]	[−87, −87, −20]	[−1.5, −1.5, −0.7]
*A* calc.	[14.5, 11.4, −0.4]	[−42.7, −41.4, −1.4]	[−2.4, −1.9, −0.5]
*A*_iso_ (exp)^b^	9.9	–65	–1.2
*A*_iso_ (calc)	8.5	–28.5	–1.6
*T* (exp)^c^	–5.6	22	0.3
*T* (calc)	–4.5	13.6	0.6
% s	–0.8	–0.16	–0.55
% p		2.8	
% d	31		
% total	92	5.2	–1.7

a The total electron densities summed over all equivalent nuclei are also given. Calculated values were obtained from DFT. From the D-band EPR spectrum of **1-Y**, we determined an experimental *g* = [1.9957, 1.9957, 1.9650].

b *A*_iso_ = (2*A*_⊥_ + *A*_∥_)/3.

c *T* = (*A*_∥_ – *A*_⊥_)/3. Calculated values of *A*_iso_ and *T* were determined using the average values reported in Table S12.

The electron density analysis indicates that the unpaired electron predominantly resides in a d orbital of each Y and is equally distributed among the three Y centers. Combined with the small deviation of the *g* factor from the free electron *g* noted above, the EPR results are in agreement with the presence of a three-center, one-electron σ bond formed mainly from the 4d_z^2^_ orbital of each Y. The hyperfine coupling parameters and spin densities obtained from the experiment for ^89^Y and ^2^D are in good agreement with the values calculated using DFT. However, in the case of ^127^I, the DFT-calculated values are significantly smaller than those obtained experimentally (see Tables 1, S12, and S13).

Variable-temperature CW X-band spectra collected for solution samples of **1-Y** and **1-Y**_**D**_ in methylcyclohexane change noticeably from 150 to 200 K (Figure S43), as a result of melting of the frozen solutions and thus a transition from immobilized to rapidly-tumbling paramagnetic species. Molecular tumbling results in a collapse of the anisotropic hyperfine and *g* tensors to the isotropic values (*A*_iso_ and *g*_iso_). At the highest temperature measured for each compound (283 K for **1-Y** and 289 K for **1-Y**_**D**_), the ^127^I hyperfine splitting becomes isotropic and the spectra are sharpened as a result, allowing the ^89^Y and ^1^H hyperfine splittings to be resolved. The spectrum for **1-Y** was well simulated with three equivalent Y and three equivalent ^1^H nuclei using |*A*_iso_ (^89^Y)| = 8.8 MHz and |*A*_iso_ (^1^H)| = 7.82 MHz (along with two |*A*_iso_ (^127^I)| = 60 MHz; Figure S44), confirming that the unpaired electron is fully delocalized over the trinuclear core. The value of |*A*_iso_ (^1^H)| = 7.82 MHz corresponds to |*A*_iso_ (^2^H)| = 1.20 MHz for **1-Y**_**D**_, which is too small to be resolved, and hence the spectrum for **1-Y**_**D**_ was simulated using only three |*A*_iso_ (^89^Y)| = 8.8 MHz and two |*A*_iso_ (^127^I)| = 60 MHz (Figure S44). These isotropic hyperfine parameters for ^127^I and ^89^Y are slightly smaller than those obtained from pulsed measurements (Table 1). This difference might indicate a small degree of electron delocalization out of the Y_3_H_3_I_2_ core into the cyclopentadienyl rings at high temperature.

### Electrochemistry

**Figure 3:**
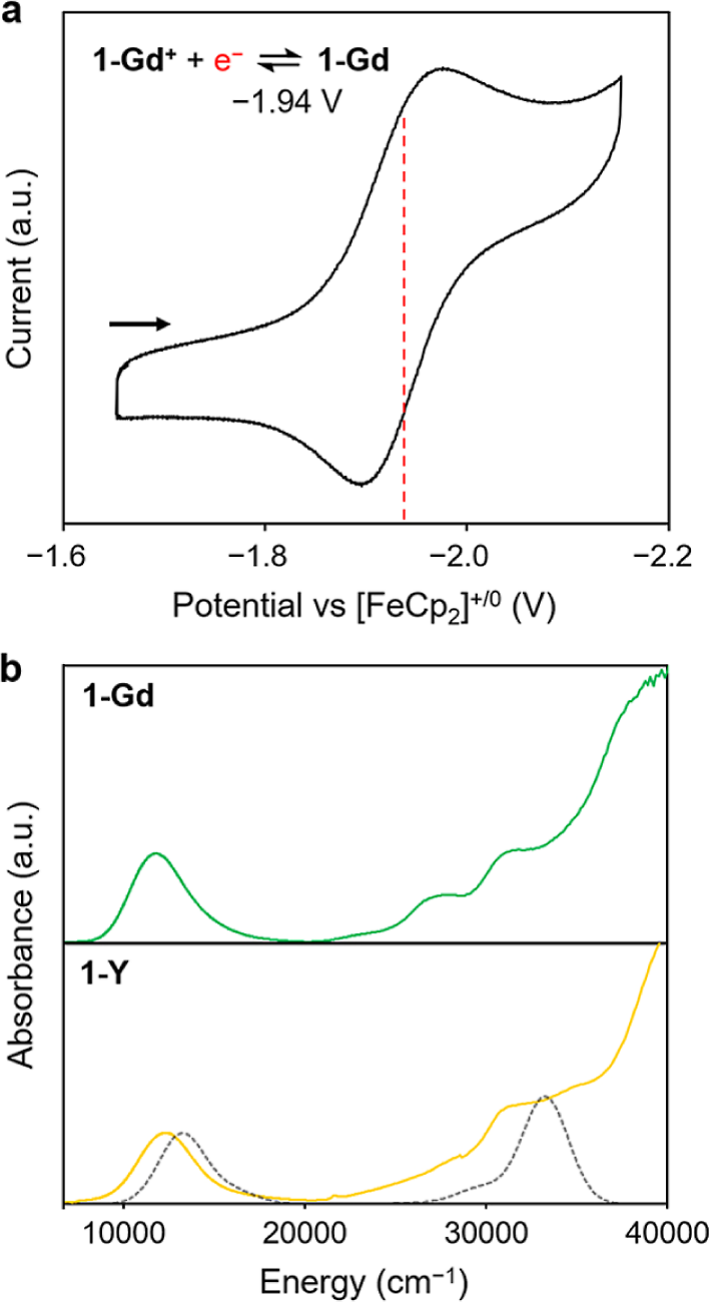
(a) Cyclic voltammogram for **1-Gd**^**+**^ in 1,2-difluorobenzene with a sweep rate of 100 mV/s. (b) UV–vis–NIR spectra for **1-Gd** (green) and **1-Y** (yellow) in *n*-hexane. The calculated spectrum for **1-Y** obtained using SA-CASSCF–MCPDFT methods is shown in gray.

Cyclic voltammetry was used to probe the redox properties of **1-Gd**^**+**^ at room temperature in 1,2-difluorobenzene. A voltammogram collected using a sweep rate of 100 mV/s (Figures 3a and S20) features a quasi-reversible, one-electron couple with a reduction potential of *E*_1/2_ = −1.94 V versus [Cp_2_Fe]^0/+^ (Δ*E* = 80.9 mV) that is assigned to the formation of **1-Gd**. Additional irreversible peaks were detected at more negative potentials, suggesting that lower-valence species may also be chemically accessible (Figure S20). Of note, the one-electron reduction potential is considerably lower than the spectroscopically determined Gd^3+^/Gd^2+^ reduction potential (−4.53 V versus [Cp_2_Fe]^0/+^)^14,21,22^ (see Section 4 of the Supporting Information) and also lower than experimental Gd^3+^/Gd^2+^ reduction potentials measured for Cp′_3_Ln (Cp′ = C_5_H_4_SiMe_3_) and Cp^tet^_3_Ln (Cp^tet^ = C_5_Me_4_H) (−2.98 and −3.04 V versus [Cp_2_Fe]^0/+^).^23^ The much smaller negative Gd^3+^/Gd^2+^ reduction potential for **1-Gd**^**+**^ is attributed to stabilization of the reduced species as a result of valence delocalization and the σ-bonding interaction.^4,7,24^

### UV–Vis–NIR Spectroscopy

UV–vis–NIR spectra were collected for solutions of **1-Y** and **1-Gd** in *n*-hexane to probe valence delocalization. At room temperature, the spectra exhibit broad NIR features (ε_max_ ≥ 5900 M^–1^ cm^–1^) at ν_max_ ≈ 12000 and 11700 cm^–1^, respectively, which we assign as intervalence charge-transfer (IVCT) bands (Figure 3b). Diffuse reflectance spectra collected for powder samples of **1-Y** and **1-Gd** exhibit similar features in the visible region (Figure S31 and S32). For **1-Y**, CASSCF multiconfigurational pair-density-functional theory (MCPDFT) calculations indicate that the band at 831 nm is a σ-to-σ* transition, while the shoulder around 330 nm is a σ-to-π transition (Figure 3b and Table S11). The full-width-at-half-maximum bandwidths of the IVCT features for **1-Y** and **1-Gd** are Δν_1/2_ = 3979 and 3708 cm^–1^, respectively, narrower than the theoretical bandwidths of ν_1/2_° = 5272 and 5204 cm^–1^.^25^ Using the experimental and calculated values for each compound, we extracted Γ = 1 – Δν_1/2_/ν_1/2_°, which can be used to classify mixed-valency.^3,26,27^ For **1-Y** and **1-Gd**, the Γ values are 0.26 and 0.29, respectively, consistent with the Robin-Day Class II–III formalism.

### Magnetic Susceptibility

**Figure 4:**
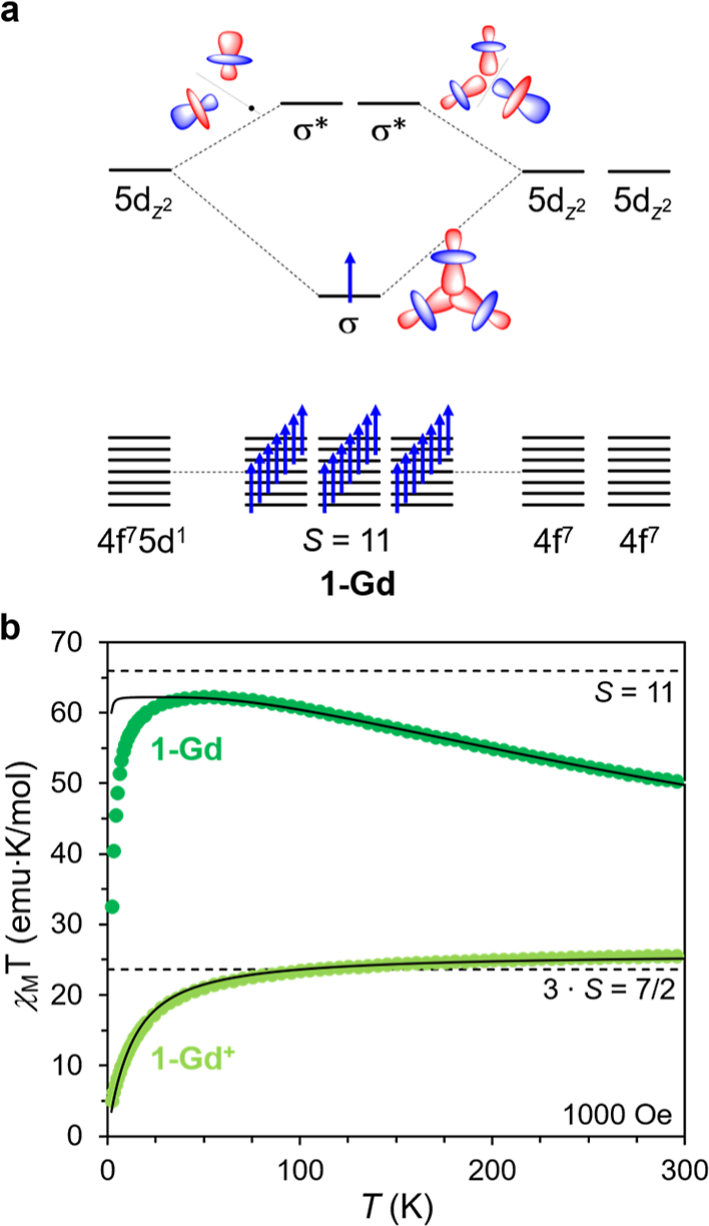
(a) Simplified molecular orbital diagram for **1-Gd** illustrating the formation of a singly occupied three-center σ-bonding orbital of 5d_z^2^_ parentage and spin–spin coupling between the σ and 4f electrons. (b) Variable temperature dc magnetic susceptibility data for **1-Gd** (dark green) and **1-Gd**^**+**^ (yellow green). Dotted lines correspond to the predicted values of χ_M_*T* and solid black lines represent fits to the data obtained using the program PHI.^28^

In the dinuclear mixed-valence complexes (Cp^iPr5^)_2_Ln_2_I_3_ (Ln = Gd, Tb, Dy), there is strong alignment of the σ-bonding electron and the f electrons on both lanthanides. We hypothesized that the shared electron in **1-Gd** would play a similar role, giving rise to a large-spin ground state by aligning the 4f electrons on all three Gd centers according to Hund’s rules (Figure 4a). To test this hypothesis, dc magnetic susceptibility data were collected for polycrystalline samples of **1-Gd**^**+**^ and **1-Gd** under a field of 1000 Oe (Figure 4b; see Section 6 of the Supporting Information). For **1-Gd**^**+**^, the magnitude of χ_M_*T* at 300 K is 25.54 emu K/mol, in reasonable agreement with that expected for three magnetically isolated *S* = ^7^/_2_ Gd^3+^ ions (23.63 emu K/mol for *g* = 2.00). The magnitude of χ_M_*T* decreases gradually with decreasing temperature until a more significant decrease below 50 K, which we ascribe to weak antiferromagnetic coupling between the Gd^3+^ ions. Indeed, these data could be well fit using an isotropic exchange Hamiltonian for a trinuclear exchange coupled cluster^28^ to give a weak antiferromagnetic exchange constant of *J*_Gd–Gd_ = −0.3 cm^–1^ between the Gd^3+^ ions.

In the case of **1-Gd**, the magnitude of χ_M_*T* is 50.08 emu K/mol at 300 K, more than twice the value of 24.00 emu K/mol predicted for three non-interacting Gd^3+^ ions and one *S* = ^1^/_2_ radical (*g* = 2.00), indicative of strong exchange coupling. The χ_M_*T* product increases monotonically with decreasing temperature, reaching a maximum of 62.27 emu K/mol at 50 K, which is close to that expected for parallel alignment of the 21 4f electrons from the three Gd centers with the unpaired electron in the σ-bonding orbital (*S* = 11, 66.00 emu K/mol). The slight deviation of the maximum value from the theoretical value could be due to a combination of Zeeman splitting of the high-spin ground state, weak antiferromagnetic 4f–4f exchange coupling (which would lower the energy of *S* < 11 excited states), a *g* value slightly less than 2, and/or zero-field splitting of the *S* = 11 ground state. The variable-temperature χ_M_*T* data for **1-Gd** were fit using a Heisenberg Hamiltonian for a symmetric exchange-coupled model (Figures 4b and S37, Table S7; see Section 6 of the Supporting Information), yielding *J*_4f−σ_ = 168(1) cm^–1^ (−2*J* formalism).^28^ This gives rise to an *S* = 11 ground state with a pair of *S* = 10 first excited states at 168 cm^–1^. The drop in χ_M_*T* at the lowest temperatures (*T* ≤ 50 K) was modeled as originating from zero-field splitting of the ground *S* = 11 state with *D* = 4.2(2) cm^–1^ and Zeeman splitting, the latter effect being more pronounced at higher fields (Figure S35). Of note, the experimental exchange constant for **1-Gd** is the second highest reported to date for a molecular gadolinium compound, the same within error as the value of *J* = 170(10) cm^–1^ reported for Gd_2_@C_79_N^29^ and superseded only by *J*_4f−σ_ = 387(4) cm^–1^ determined for Cp^iPr5^_2_Gd_2_I_3_.^3^

Broken-symmetry DFT calculations using the crystal structure for **1-Gd** predict *J*_4f−σ_ = 176 cm^–1^ (with *J*_Gd–Gd_ = −2.2 cm^–1^; Table S14 and Figure S47), in excellent agreement with the experimental value of *J*_4f−σ_ = 168(1) cm^–1^. On the other hand, CASSCF calculations predict an average value of *J*_4f−σ_ = 333 cm^–1^, while CASSCF–MCPDFT calculations afford an average value of *J*_4f−σ_ = 219 cm^–1^ (Figure S48 and Table S15). Our CASSCF calculations do not account for dynamic correlation effects and so are dominated by direct exchange terms: this clearly overestimates the *J*_4f−σ_ exchange coupling for **1-Gd**. The addition of dynamic correlation with MCPDFT lowers the predicted coupling magnitude, and the use of DFT (here using B3LYP, which has a 20% fraction of exact Hartree–Fock exchange) lowers it further toward the experimental value. These results are distinct from those obtained in our recent analysis of Cp^iPr5^_2_Gd_2_I_3_,^3^ where equivalent CASSCF-like calculations only slightly overestimated the experimental *J*_4f−σ_ magnitude, and DFT calculations with B3LYP significantly underestimated the value. As a result, therein we determined that the direct exchange mechanism dominated *J*_4f−σ_ for Cp^iPr5^_2_Gd_2_I_3_.^3^ In contrast, the results for **1-Gd** indicate that direct exchange is likely responsible for the large spin–spin coupling (i.e., the parallel alignment of the 4f and σ electrons obeys Hund’s rule), but that significant superexchange and/or kinetic (double) exchange contributions result in a lower calculated *J*_4f−σ_ than determined for Cp^iPr5^_2_Gd_2_I_3_.

## Conclusions

We have reported the synthesis and characterization of (Cp^iPr5^)_3_Ln_3_H_3_I_2_ (**1-Ln**; Ln = Y, Gd), which feature a three-center, one-electron σ bond of 4d_z^2^_ or 5d_z^2^_ parentage. Direct metal–metal orbital overlap induces strong electron delocalization that corresponds to a Robin-Day Class II–III formalism, as supported by EPR and UV–vis–NIR spectroscopy data. In **1-Gd**, the σ-bonding electron engages in strong spin–spin coupling with the 4f electrons on all three lanthanide centers, giving rise to a high-spin *S* = 11 ground state. We are currently investigating the properties of **1-Ln** for lanthanide ions such as Tb or Dy that possess large single-ion magnetic anisotropy. The chemistry developed here suggests that with judicious design, it may be possible to obtain even higher-nuclearity clusters featuring Ln–Ln bonding interactions, with potential relevance to the discovery of new high-performance single-molecule magnets.

## Supporting Information Available

Synthetic procedures, IR, UV–vis–NIR, and EPR spectroscopy data, X-ray crystallography data, CV data, magnetic data, and computational data (PDF)
