# Supplementary material for: A Trinuclear Gadolinium Cluster with a Three-Center One-Electron Bond and an S = 11 Ground State
Source: J Am Chem Soc. 2023 Apr 17;145(16):8996–9002. doi: 10.1021/jacs.3c00182 (PMC10141408; doi:10.1021/jacs.3c00182)
Supplement: Supplementary file 1 — ja3c00182_si_001.pdf [file ja3c00182_si_001.pdf]

Electronic Supplementary Information for:

A Trinuclear Gadolinium Cluster with a Three-Center One-Electron Bond and an *S* = 11 Ground State

K. Randall McClain,[†,‡] Hyunchul Kwon,[§,‡] Khetpakorn Chakarawet,[⊥,#‡] Rizwan Nabi,[£] Jon G. C. Kragskow,[£] Nicholas F. Chilton,[*,£] R. David Britt,[*,⊥] Jeffrey R. Long,[*,§,∥,¶] and Benjamin G. Harvey[*,†]

‡These authors contributed equally

[†]US Navy, Naval Air Warfare Center, Weapons Division, Research Department, Chemistry Division, China Lake, California, 93555, United States

[§]Department of Chemistry and [∥]Department of Chemical and Biomolecular Engineering, University of California, Berkeley, Berkeley, California 94720, United States

[⊥]Department of Chemistry, University of California, Davis, Davis, California 95616, United States

[£]Department of Chemistry, The University of Manchester, Manchester M13 9PL, United Kingdom

[¶]Materials Sciences Division, Lawrence Berkeley National Laboratory, Berkeley, California 94720, United States

[#]Department of Chemistry, Faculty of Science, Mahidol University, Bangkok 10400, Thailand

Table of Contents

1. Experimental/Methods & Synthesis of All Compounds S3

2. Infrared Spectra for $(Cp^{iPr5})_3Y_3H_3I_3$, $(Cp^{iPr5})_3Gd_3H_3I_3$, **1-Y**, **1-Y$_D$**, **1-Gd**, and **1-Gd$^+$** S7

3. X-ray Crystallography Data Collection and Refinement Details S11

4. Cyclic Voltammetry Data for **1-Gd$^+$** S23

5. UV-Vis-NIR Spectra for **1-Y**, **1-Gd**, and **1-Gd$^+$** S24

6. Magnetization and Dc Magnetic Susceptibility Data for **1-Gd** and **1-Gd$^+$** S31

7. EPR Spectroscopy Data for **1-Y** and **1-Y$_D$** S34

8. Computational Methods and Analysis S43

9. References S67

1. Experimental/Methods & Synthesis of All Compounds

General Procedures. Unless otherwise specified, all manipulations were performed using Schlenk or glovebox techniques under an atmosphere of purified argon with rigorous exclusion of water and oxygen. All solvents were purchased from Sigma-Aldrich as anhydrous grade in Sure/Seal™ bottles, purged for several hours with purified argon, and stored over activated 3 Å molecular sieves in an argon filled glovebox. Celite (AW Standard Super-Cel® NF) was purchased from Sigma-Aldrich and dried under vacuum at 150–200 °C overnight before being transferred to the glovebox. Potassium graphite (KC_8) was purchased from Strem and used as received or synthesized by the reaction of graphite with potassium at ~100 °C under argon. Anhydrous YI_3 and GdI_3 were purchased from Alfa Aesar as Ultra Dry™ grade reagents and used as received. The ligand salt $NaCp^{iPr5}$ was prepared using a previously published method.[1] The compounds $(Cp^{iPr5})_2Ln_2I_4$ (Ln = Y, Gd) were prepared using the previously reported procedure.[2] We note that, in our hands, these dinuclear precursors could only be isolated with iodide. Indeed, our attempts to prepare analogous dinuclear compounds with bromide or chloride resulted in the formation of trinuclear species $(Cp^{iPr5})_3Ln_3X_6$ (X = Br^- or Cl^-) with all bridging halides, likely a consequence of the smaller covalent/ionic radii of these anions and their greater propensity for engaging in bridging interactions. The compound $[H(SiEt_3)_2][B(C_6F_5)_4]$ was synthesized via a previously published method and used shortly after preparation.[3] FT-IR spectra were recorded on a Perkin Elmer Avatar Spectrum 400 FTIR Spectrometer equipped with an attenuated total reflectance (ATR) attachment. UV-vis-NIR absorption spectra were collected with a CARY 5000 spectrophotometer interfaced with Varian WinUV software. Matrix Assisted Laser Desorption Ionization Time of Flight (MALDI-TOF) mass spectra were recorded on an Applied Biosystems Voyager-DE PRO Workstation in positive ion mode. Samples were co-crystalized in an anthracene matrix on an AB SCIEX MALDI-TOF stainless steel sample plate. Spectra were averaged over 200 laser pulses with a low mass gate of 300 Dalton and a high mass gate of 2000 Dalton. Elemental analyses (C, H, N) were performed by the Microanalytical Facility at the University of California, Berkeley using a Perkin-Elmer 2400 Series II combustion analyzer. Magnetic susceptibility measurements were collected using a Quantum Design MPMS2 SQUID magnetometer.

Synthesis of $(Cp^{iPr5})_2Y_2(CH_2SiMe_3)_2I_2$. Under argon, $(Cp^{iPr5})_2Y_2I_4$ (0.500 g / 0.404 mmol) and benzene (50 mL) were combined in a 100 mL Schlenk flask to give a mostly colorless solution. Solid $LiCH_2SiMe_3$ (0.076 g / 0.81 mmol) was added and the reaction mixture was stirred at room temperature for 24 h, during which time the formation of a colorless precipitate was noted. The solvent was removed under vacuum to leave a mostly colorless solid residue, which was slurried by stirring with pentane (40 mL) for 30 min, then filtered through Celite. The filter pad was extracted with additional pentane (2 × 10 mL), to give a pale-yellow filtrate which was concentrated to 25 mL and left at −35 °C. Colorless plate crystals were obtained in multiple crops, washed with a small amount of cold (−35 °C) pentane and dried under vacuum (0.203 g / 0.175 mmol / 43% based on $(Cp^{iPr5})_2Y_2I_4$). MALDI ToF MS *m/z*: 982.75 ($[M{-}2CH_2Si(CH_3)_3]^+$). $C_{48}H_{92}I_2Si_2Y_2$ (1157.06): calcd (%) C 49.83, H 8.01; found (%) C 49.46, H 7.78.

Synthesis of $(Cp^{iPr5})_2Gd_2(CH_2SiMe_3)_2I_2$. Under argon, $(Cp^{iPr5})_2Gd_2I_4$ (0.500 g / 0.364 mmol) and benzene (50 mL) were combined in a 100 mL Schlenk flask to give a pale-yellow solution. Solid $LiCH_2SiMe_3$ (0.069 g / 0.73 mmol) was added and the reaction mixture was stirred at room temperature for 24 h, during which time the formation of a yellow/amber suspension with colorless precipitate was noted. The solvent was removed under vacuum to leave a yellow/amber solid

residue, which was slurried by stirring with pentane (40 mL) for 30 min, then filtered through Celite. The filter pad was extracted with additional pentane (2 × 10 mL), to give a yellow/amber filtrate which was concentrated to 25 mL and left at −35 °C. Yellow plate crystals were obtained in multiple crops, washed with a small amount of cold (−35 °C) pentane and dried under vacuum (0.230 g / 0.178 mmol / 49% based on $(Cp^{iPr5})_2Gd_2I_4$). MALDI ToF MS *m/z*: 1119.35 ($[M-2CH_2Si(CH_3)_3]^+$) and 559.10 ($[M-2CH_2Si(CH_3)_3]^{2+}$). $C_{48}H_{92}I_2Si_2Gd_2$ (1293.74): calcd (%) C 49.83, H 8.01; found (%) C 49.46, H 7.78.

Synthesis of $(Cp^{iPr5})_3Y_3H_3I_3$. Under argon, $(Cp^{iPr5})_2Y_2I_4$ (0.400 g / 0.323 mmol) and benzene (50 mL) were combined in a 100 mL Schlenk flask to give a colorless solution. Solid $LiCH_2SiMe_3$ (0.061 g / 0.65 mmol) was added and the reaction mixture was stirred at room temperature for 24 h, during which time the formation of a colorless precipitate was noted. The solvent was removed under vacuum to leave a mostly colorless solid residue, which was slurried by stirring with n-hexane (30 mL) for 30 min, then filtered through Celite. The filter pad was extracted with additional n-hexane (2 × 5 mL), to give a pale-yellow filtrate. This solution containing crude $(Cp^{iPr5})_2Y_2(CH_2SiMe_3)_2I_2$ was added to a Fisher-Porter tube apparatus and frozen with liquid N_2 while applying dynamic vacuum. While the solution was still frozen, H_2 gas was dosed into the tube at a pressure of 80 psi for approximately 30 s, the apparatus was then sealed, and the reaction mixture was allowed to thaw and stirred at room temperature for 24 h to give a pale-yellow solution. The solvent was removed under vacuum to leave a mostly colorless sticky residue, which was taken up in pentane (25 mL), filtered through Celite, and the filter pad washed with additional pentane (2 × 5 mL) to give a pale-yellow filtrate. The filtrate was concentrated to ~2 mL and left at −35 °C. Colorless crystals were isolated in multiple crops, washed with a small amount of cold (−35 °C) pentane and dried under vacuum (0.203 g / 0.137 mmol / 64% based on $(Cp^{iPr5})_2Y_2I_4$). MALDI ToF MS *m/z*: 1201.85 ($[M-Cp^{iPr5}]^+$). $C_{60}H_{108}I_3Y_3$ (1476.95): calcd (%) C 48.79, H 7.37; found (%) C 49.08, H 7.38.

Synthesis of $(Cp^{iPr5})_3Gd_3H_3I_3$. Under argon, $(Cp^{iPr5})_2Gd_2I_4$ (0.650 g / 0.473 mmol) and benzene (50 mL) were combined in a 100 mL Schlenk flask to give a pale-yellow solution. Solid $LiCH_2SiMe_3$ (0.089 g / 0.95 mmol) was added and the reaction mixture was stirred at room temperature for 24 h, during which time the formation of a yellow/amber suspension with colorless precipitate was noted. Solvent was removed under vacuum to leave a yellow/amber solid residue, which was slurried by stirring with n-hexane (30 mL) for 30 min, then filtered through Celite. The filter pad was extracted with additional n-hexane (2 × 5 mL), to give a yellow/amber filtrate. This yellow-amber solution containing crude $(Cp^{iPr5})_2Gd_2(CH_2SiMe_3)_2I_2$ was added to a Fisher-Porter tube apparatus and frozen with liquid N_2 while applying dynamic vacuum. While the solution was still frozen, H_2 gas was dosed into the tube at a pressure of 80 psi for approximately 30 s, the apparatus was then sealed, and the reaction mixture was allowed to thaw and stirred at room temperature for 24 h to give a yellow/amber solution. Solvent was removed under vacuum to leave a yellow/amber sticky residue, which was taken up in pentane (25 mL), filtered through Celite and the pad washed with additional pentane (2 × 5 mL) to give a yellow/amber filtrate. This was concentrated to ~2 mL and left at −35 °C. Light yellow prism crystals were isolated in multiple crops, washed with a small amount of cold (−35 °C) pentane and dried under vacuum (0.296 g / 0.176 mmol / 56% based on $(Cp^{iPr5})_2Gd_2I_4$). MALDI ToF MS *m/z*: 1406.49 ($[M-Cp^{iPr5}]^+$). $C_{60}H_{108}I_3Gd_3$ (1681.99): calcd (%) C 42.85, H 6.47; found (%) C 42.75, H 6.43.

Synthesis of $(Cp^{iPr5})_3Y_3H_3I_2$ (1-Y). Under argon, $(Cp^{iPr5})_2Y_2I_4$ (0.750 g / 0.607 mmol) and benzene (50 mL) were combined in a 100 mL Schlenk flask to give a nearly colorless solution.

Solid $LiCH_2SiMe_3$ (0.114 g / 1.21 mmol) was added and the reaction mixture was stirred at room temperature for 24 h, during which time the formation of a pale yellow suspension with colorless precipitate was noted. Solvent was removed under vacuum to leave a mostly colorless solid residue, which was slurried by stirring with n-hexane (30 mL) for 30 min, then filtered through Celite. The filter pad was extracted with additional n-hexane (2 × 5 mL), to give a pale yellow filtrate. This pale yellow solution containing crude $(Cp^{iPr5})_2Y_2(CH_2SiMe_3)_2I_2$ was added to a Fisher-Porter tube apparatus and frozen with liquid N_2 while applying dynamic vacuum. While the solution was still frozen, H_2 gas was dosed into the tube at a pressure of 80 psi for approximately 30 s, the apparatus was then sealed, and the reaction mixture was allowed to thaw and stirred at room temperature for 24 h to give a pale yellow solution. Solvent was removed under vacuum to leave a mostly colorless sticky residue containing crude $(Cp^{iPr5})_3Y_3H_3I_3$, which was taken up in n-hexane (50 mL), filtered through Celite and the pad washed with additional n-hexane (2 × 10 mL) to give a pale yellow filtrate. The filtrate was transferred to a 100 mL Schlenk flask, KC_8 (0.273 g / 2.02 mmol) was added, and the mixture stirred at room temperature for 9 d. The reaction mixture was filtered through Celite to give a dark green filtrate, and the filter pad was extracted with additional n-hexane (200 mL) and filtered again over Celite. This second filtrate was combined with the first to give a dark green solution, which was concentrated to 60 mL, heated to boiling to re-dissolve green precipitate, left to cool to room temperature overnight, and then transferred to the freezer (−35 °C). Small dark green, thin rectangular, plank-like crystals of **1-Y** were isolated in multiple crops, washed with a small amount of cold (−35 °C) pentane and dried under vacuum (0.167 g / 0.124 mmol / 31% based on $(Cp^{iPr5})_2Y_2I_4$). MALDI ToF MS *m/z*: 1350.07 ($[M]^+$). $C_{60}H_{108}Y_3I_2$ (1350.05): calcd (%) C 53.38, H 8.06; found (%) C 54.26, H 8.15.

Synthesis of $(Cp^{iPr5})_3Y_3D_3I_2$ (1-Y_D). Under argon, $(Cp^{iPr5})_2Y_2I_4$ (0.500 g / 0.404 mmol) and benzene (50 mL) were combined in a 100 mL Schlenk flask to give a nearly colorless solution. Solid $LiCH_2SiMe_3$ (0.076 g / 0.81 mmol) was added and the reaction mixture was stirred at room temperature for 24 h, during which time the formation of a pale yellow suspension with colorless precipitate was noted. Solvent was removed under vacuum to leave a mostly colorless solid residue, which was slurried by stirring with n-hexane (30 mL) for 30 min, then filtered through Celite. The filter pad was extracted with additional n-hexane (2 × 5 mL), to give a pale yellow filtrate. This pale yellow solution containing crude $(Cp^{iPr5})_2Y_2(CH_2SiMe_3)_2I_2$ was added to a Fisher-Porter tube apparatus and frozen with liquid N_2 while applying dynamic vacuum. While the solution was still frozen, D_2 gas was dosed into the tube at a pressure of 80 psi for approximately 30 s, the apparatus was then sealed, and the reaction mixture was allowed to thaw and stirred at room temperature for 24 h to give a pale yellow solution. Solvent was removed under vacuum to leave a mostly colorless sticky residue containing crude $(Cp^{iPr5})_3Y_3D_3I_3$, which was taken up in n-hexane (50 mL) and filtered through Celite. The pad was washed with additional n-hexane (2 × 10 mL) to give a pale yellow filtrate. This solution was transferred to a 100 mL Schlenk flask, KC_8 (0.182 g / 1.35 mmol) was added, and the mixture was stirred at room temperature for 9 d. The reaction mixture was filtered through Celite to give a dark green filtrate, and the residue on the filter pad was then stirred with additional n-hexane (200 mL) and filtered through Celite. The filtrates were combined, and the dark green solution was concentrated to 40 mL, heated to boiling to re-dissolve a green precipitate, and left to cool to room temperature overnight, then transferred to the freezer (−35 °C). Small dark green, thin rectangular plank-like crystals of **1-Y_D** were isolated in multiple crops, washed with a small amount of cold (−35 °C) pentane and dried under vacuum (0.086 g / 0.064 mmol / 24% based on $(Cp^{iPr5})_2Y_2I_4$). MALDI ToF MS *m/z*: 1353.19 ($[M]^+$). $C_{60}H_{105}D_3Y_3I_2$ (1353.07): calcd (%) C 53.26, H 8.27; found (%) C 52.78, H 7.79.

Synthesis of $(Cp^{iPr5})_3Gd_3H_3I_2$ (1-Gd). Under argon, $(Cp^{iPr5})_2Gd_2I_4$ (0.650 g / 0.473 mmol) and benzene (50 mL) were combined in a 100 mL Schlenk flask to give a pale-yellow solution. Solid $LiCH_2SiMe_3$ (0.089 g / 0.95 mmol) was added and the reaction mixture was stirred at room temperature for 24 h, during which time the formation of a yellow/amber suspension with colorless precipitate was noted. Solvent was removed under vacuum to leave a yellow/amber solid residue, which was slurried by stirring with n-hexane (30 mL) for 30 min, then filtered through Celite. The filter pad was extracted with additional n-hexane (2 × 5 mL) to give a yellow/amber filtrate. This yellow-amber solution containing crude $(Cp^{iPr5})_2Gd_2(CH_2SiMe_3)_2I_2$ was added to a Fisher-Porter tube apparatus and frozen with liquid N_2 while applying dynamic vacuum. While the solution was still frozen, H_2 gas was dosed into the tube at a pressure of 80 psi for approximately 30 s, the apparatus was then sealed, and the reaction mixture was allowed to thaw and stirred at room temperature for 24 h to give a yellow/amber solution Solvent was removed under vacuum to leave a yellow/amber sticky residue containing crude $(Cp^{iPr5})_3Gd_3H_3I_3$, which was taken up in n-hexane (50 mL) and filtered through Celite; the pad washed with additional n-hexane (2 × 10 mL) to give a yellow/amber filtrate. This filtrate was transferred to a 100 mL Schlenk flask, KC_8 (0.213 g / 1.58 mmol) was added, and the mixture stirred at room temperature for 9 d. The reaction mixture was filtered through Celite to give a dark blue filtrate, and the residue remaining on the filter pad extracted with additional n-hexane (200 mL) and filtered through Celite. The filtrates were combined, concentrated to 60 mL, heated to boiling to re-dissolve a blue precipitate, left to cool to room temperature overnight, then transferred to the freezer (−35 °C). Small dark green-blue, thin rectangular plank-like crystals of **1-Gd** were isolated in multiple crops, washed with a small amount of cold (−35 °C) pentane and dried under vacuum (0.120 g / 0.077 mmol / 24% based on $(Cp^{iPr5})_2Gd_2I_4$). A separate batch of **1-Gd** crystals was also grown from a concentrated methylcyclohexane solution in the freezer (−35 °C). MALDI ToF MS *m/z*: 1555.55 ($[M]^+$). $C_{60}H_{108}Gd_3I_2$ (1555.08): calcd (%) C 46.34, H 7.00; found (%) C 46.55, H 6.98.

Synthesis of $[(Cp^{iPr5})_3Gd_3H_3I_2][B(C_6F_5)_4]$ (1-Gd$^+$). Under argon, $(Cp^{iPr5})_3Gd_3H_3I_3$ (0.250 g / 0.149 mmol) and $[H(SiEt_3)_2][B(C_6F_5)_4]$ (0.135 g / 0.149 mmol) were combined in a 100 mL pressure flask and n-hexane (50 mL) was added. The flask was sonicated for 2 days at −5 °C, during which time the formation of a fine yellow powder was noted. The contents of the flask were filtered and the obtained yellow powder was washed with pentane (3 × 15 mL). The crude yellow powder was dissolved in cold (−35 °C) fluorobenzene (3 mL), then filtered to give a yellow solution which was layered with cold (−35 °C) pentane (5 mL) in an 8 mL vial and left at −35 °C. After several days, yellow crystals were obtained, washed with a small amount of cold (−35 °C) pentane and dried under vacuum. These crystals were dissolved in cold (−35 °C) fluorobenzene (5 mL) to give a yellow solution which was filtered and layered with pentane (25 mL) in a 40 mL vial and left at −35 °C. After several days, yellow crystals were obtained, washed with a small amount of cold (−35 °C) pentane and dried under vacuum. This recrystallization procedure was repeated multiple times until analytically pure yellow crystals of **1-Gd$^+$** were obtained (0.045 g / 0.020 mmol / 13% based on $(Cp^{iPr5})_3Gd_3H_3I_3$). MALDI ToF MS *m/z*: 1555.5 ($[M−B(C_6F_5)_4]^+$). $C_{84}H_{108}BF_{20}Gd_3I_2$ (2234.13): calcd (%) C 45.16, H 4.87; found (%) C 44.80, H 4.69.

2. Infrared Spectra for $(Cp^{iPr5})_3Y_3H_3I_3$, $(Cp^{iPr5})_3Gd_3H_3I_3$, 1-Y, 1-Y_D, 1-Gd, and 1-Gd^+

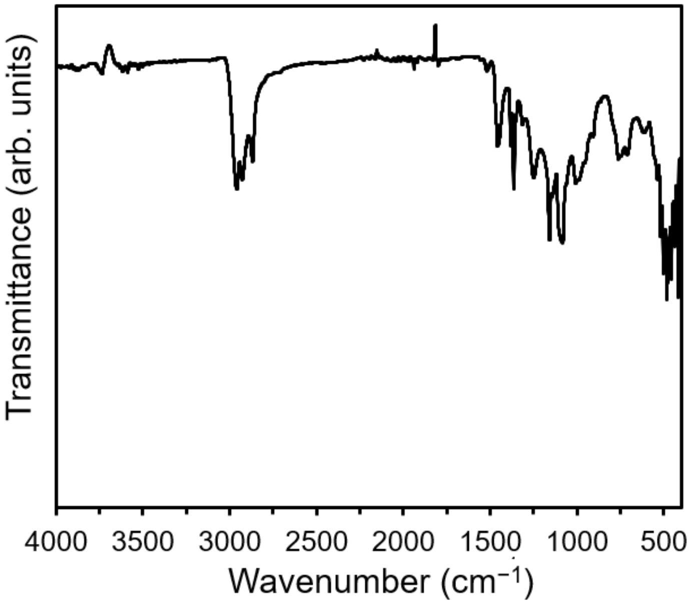

Figure S1. IR spectrum of $(Cp^{iPr5})_3Y_3H_3I_3$.

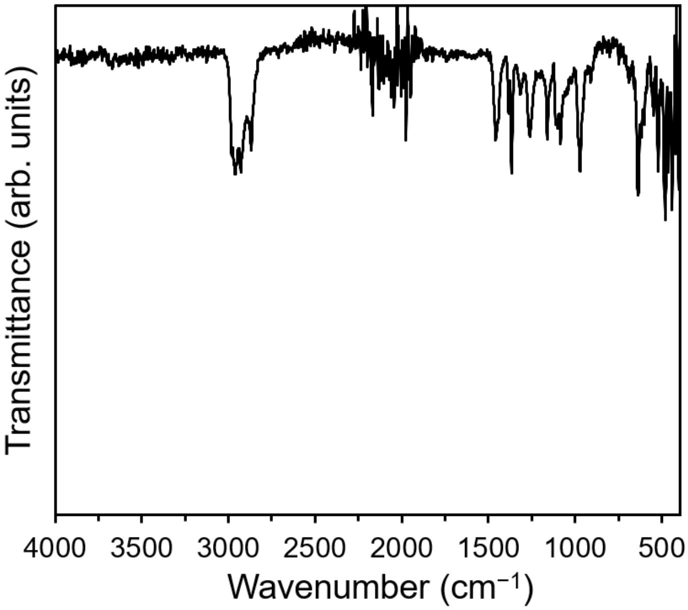

Figure S2. IR spectrum of $(Cp^{iPr5})_3Y_3H_3I_2$, **1-Y**.

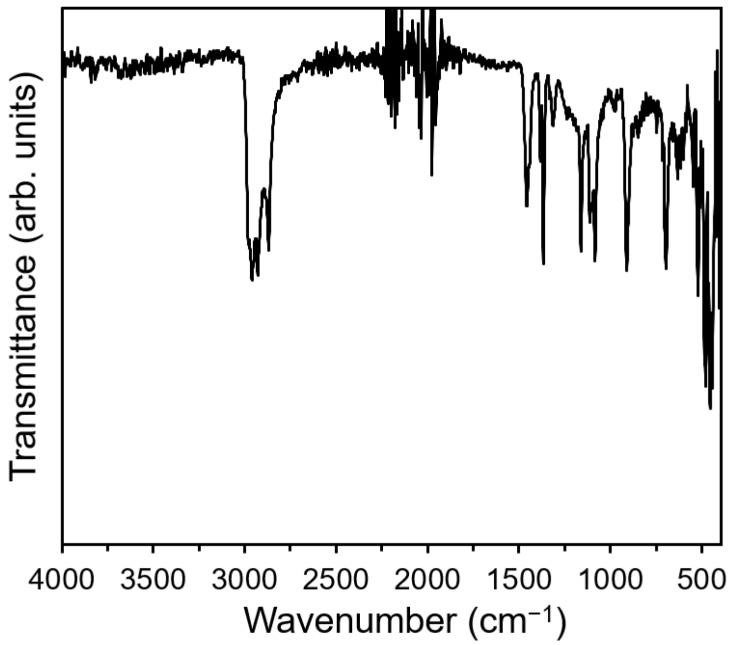

Figure S3. IR spectrum of $(Cp^{iPr5})_3Y_3D_3I_2$, **1-Y$_D$**.

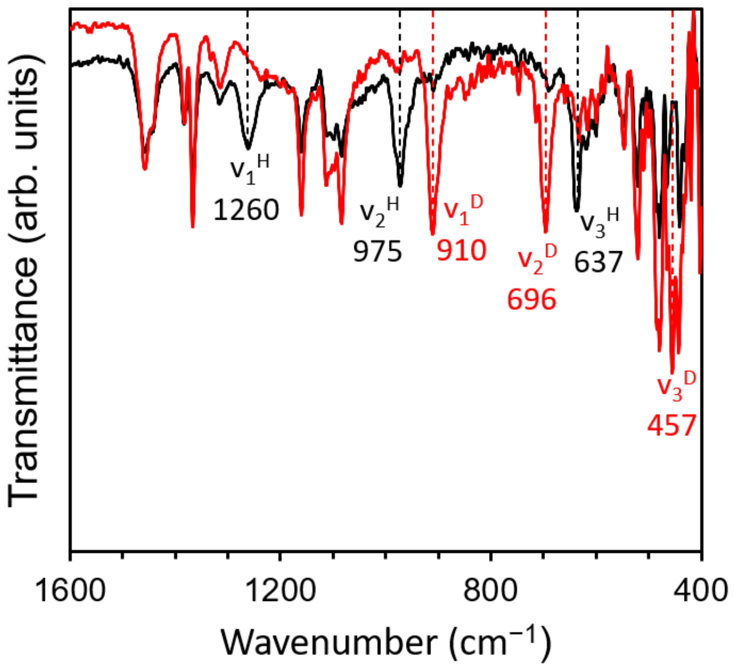

Figure S4. Comparison of infrared spectra collected for **1-Y** (black trace) and **1-Y$_D$** (red trace) in the region between 400 and 1600 cm^{-1}. The peaks originating from Y–H vibrations and the corresponding Y–D peaks are labeled. See Section 8 below for a detailed discussion of the computed and experimental IR spectra for **1-Y** and **1-Y$_D$**.

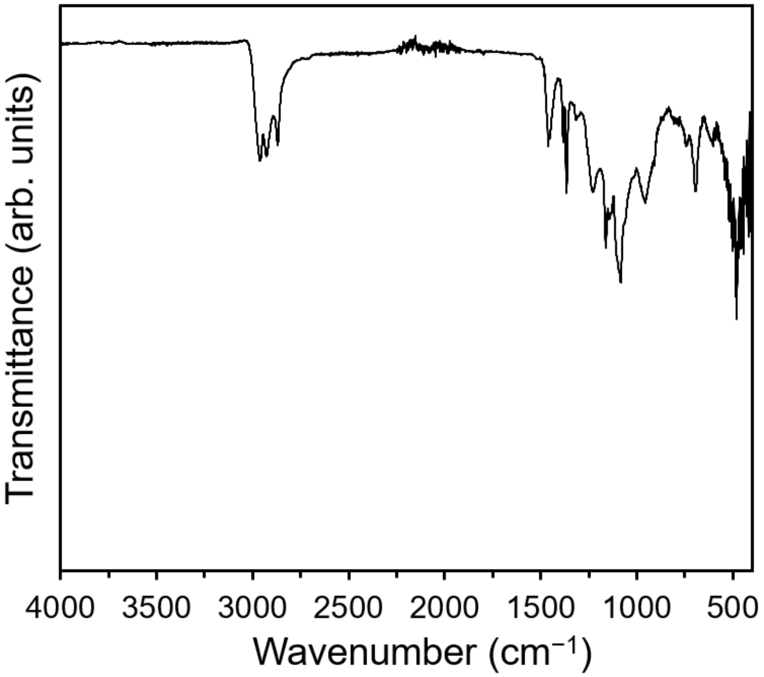

Figure S5. Infrared spectrum of $(Cp^{iPr5})_3Gd_3H_3I_3$.

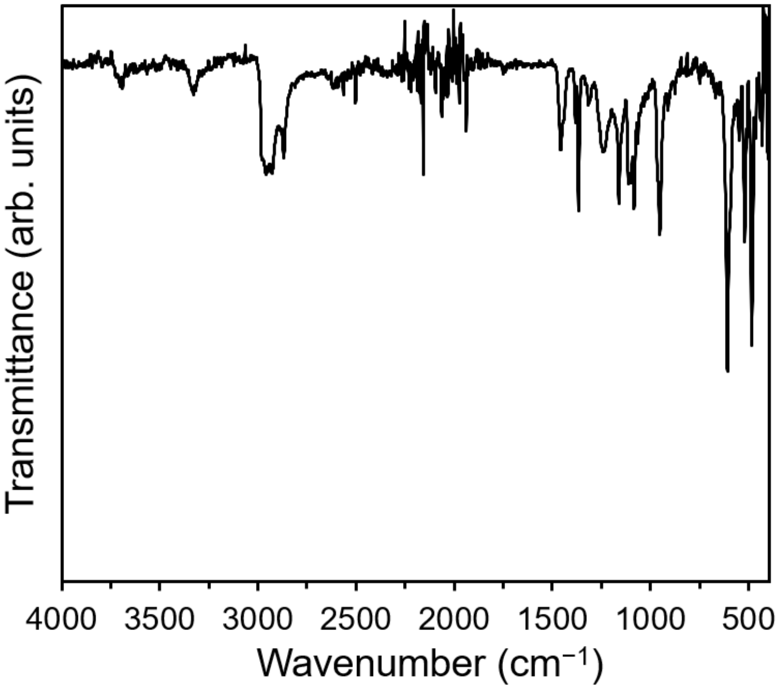

Figure S6. Infrared spectrum of $(Cp^{iPr5})_3Gd_3H_3I_2$, **1-Gd**.

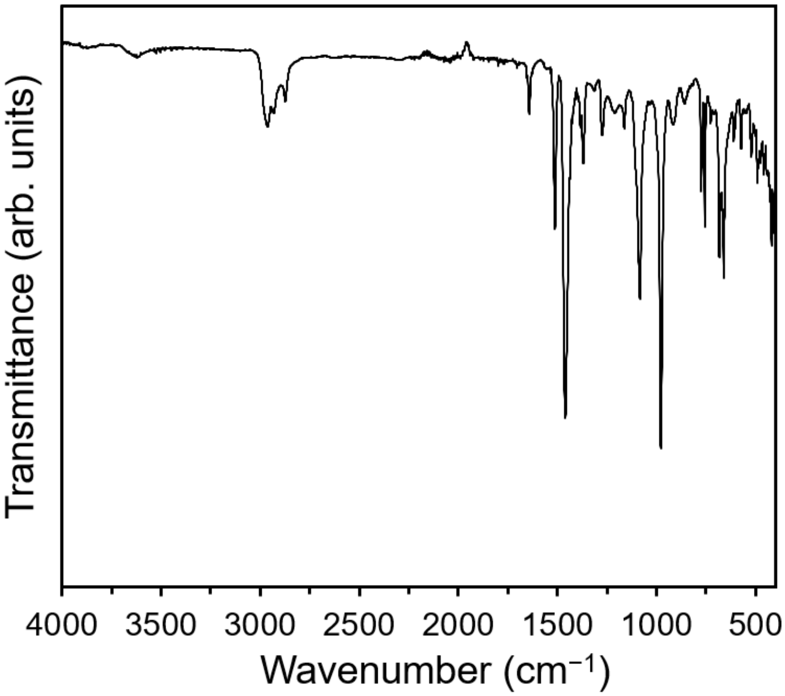

Figure S7. Infrared spectrum of $[(Cp^{iPr5})_3Gd_3H_3I_2][B(C_6F_5)_4]$, **1-Gd**$^+$.

3. X-ray Crystallography Data Collection and Refinement Details

Structure solutions were determined by SHELXT using direct methods and these solutions were refined via least-square refinement against F^2 by SHELXL, as implemented in OLEX2 crystallographic software.[4,5] For all structures, all non-hydrogen atoms were refined anisotropically. All hydrogen atoms except hydride ligands were placed on geometrically calculated positions using the riding model and refined isotropically.

$(Cp^{iPr5})_2Y_2(CH_2SiMe_3)_2I_2$. The compound crystallized in the space group $P\overline{1}$ with one molecule in the asymmetric unit. The Cp^{iPr5} ligands showed positional disorder over two sites, and the occupancies of these two components were refined while constraining the sum to unity, yielding a ratio of 0.501(5):0.499(5) and 0.727(10):0.273(10), respectively. The application of the standard combination of SIMU, DELU restraints and EADP constraints to the displacement parameters of the cyclopentadienyl carbons—and EADP constraints to the displacement parameters of the analogous carbon atoms bound to the Y atom in the CH_2SiMe_3—groups gave free R factors of R_1 = 3.38% and wR_2 = 7.85%. In checkcif, one alert B (PLAT230_ALERT_2_B) was generated from the metal bound carbon atoms in the structure. No A alerts were found.

$(Cp^{iPr5})_2Gd_2(CH_2SiMe_3)_2I_2$. The compound crystallized in the space group $P\overline{1}$ with half of a molecule in the asymmetric unit and an inversion center located in the middle of two Gd atoms within the molecule. The Cp^{iPr5} ligand, the methylene carbon atom in the CH_2SiMe_3 group, and the iodide ligand showed positional disorder over two sites, and the occupancies of these two components were refined while constraining the sum to unity, yielding ratios of 0.576(10):0.424(10), 0.52(3):0.48(3), and 0.9446(17):0.0554(17), respectively. The application of the standard combination of SIMU, DELU restraints to the displacement parameters of the cyclopentadienyl carbons, and EADP constraints to the displacement parameters of the disordered carbon atom in the CH_2SiMe_3 group and the disordered iodine atom gave free R factors of R_1 = 3.10% and wR_2 = 7.07%. In checkcif, two level B alerts (PLAT213_ALERT_2_B and PLAT230_ALERT_2_B) were generated from the metal bound carbon atoms in the structure.

$(Cp^{iPr5})_3Y_3H_3I_3$. The compound crystallized in the space group $P2_1/n$ with one molecule in the asymmetric unit. Solvent mask, as implemented in Olex2 (analogous to SQUEEZE), was used to remove the electron density from the highly disordered solvent molecules in the lattice (V = 1754 Å^3, 300 e$^-$). One Cp^{iPr5} ligand and the three iodide ligands showed positional disorder over two sites, and the occupancies of these two components were refined while constraining the sum to unity, yielding a ratio of 0.673(9):0.327(9) and 0.9608(7):0.0392(7), respectively. The hydride ligands near the Y atoms were located manually using distance restraints (DFIX or SADI). The application of EADP constraints to the displacement parameters of the cyclopentadienyl carbons and iodide ligands gave free R factors of R_1 = 4.84% and wR_2 = 13.44%. In checkcif, four level B alerts (PLAT971_ALERT_2_B) were generated from positive residual density near the iodide ligands. This residual density could not be modelled as any chemically reasonable species, and it likely arises due to strongly absorbing Y or I or due to problems with the absorption correction.

$(Cp^{iPr5})_3Gd_3H_3I_3$. The compound crystallized in the space group $P2_1/n$ with one molecule in the asymmetric unit. Solvent mask, as implemented in Olex2 (analogous to SQUEEZE), was used to remove the electron density from the highly disordered solvent molecules in the lattice (V = 1736 Å^3, 1018 e$^-$). One Cp^{iPr5} ligand and iodide ligands showed positional disorder over two sites, and the occupancies of these two components were refined while constraining the sum to unity, yielding a ratio of 0.730(5):0.270(5) and 0.9678(6):0.0322(6), respectively. The hydride ligands

near the Y atoms were located manually using distance restraints (DFIX or SADI). The application of the standard combination of SIMU, DELU restraints and EADP constraints to the displacement parameters of the cyclopentadienyl carbons and iodide ligands gave free R factors of R_1 = 3.25% and wR_2 = 9.05%. In checkcif, one level B alert (PLAT971_ALERT_2_B) were generated from positive residual density near the iodide ligands. This residual density could not be modelled as any chemically reasonable species and likely arises due to strongly absorbing Gd or I or due to problems with the absorption correction.

$(Cp^{iPr5})_3Y_3H_3I_2$ (1-Y). The compound crystallized in the space group *Pbca* with one molecule in the asymmetric unit. The structural refinement gives free R factors of R_1 = 5.31% and wR_2 = 13.50%. In checkcif, one level B alert (PLAT971_ALERT_2_B) was generated from positive residual density near the iodide ligands. This residual density could not be modelled as any chemically reasonable species, and it likely arises due to the strongly absorbing Y or I ions or due to problems with the absorption correction.

$(Cp^{iPr5})_3Y_3D_3I_2$ (1-Y_D). The compound crystallized in the space group *Pbca* with one molecule in the asymmetric unit. The structural refinement gives free R factors of R_1 = 4.73% and wR_2 = 12.44%. In checkcif, three level B alerts (PLAT971_ALERT_2_B and PLAT972_ALERT_2_B) were generated from positive residual density near the iodide ligands. This residual density could not be modelled as any chemically reasonable species and likely arises due to the strongly absorbing Y or I ions or due to problems with the absorption correction.

$(Cp^{iPr5})_3Gd_3H_3I_2$ (1-Gd). When crystallized from n-hexane, the compound crystallized in the space group *Pbca* with one molecule in the asymmetric unit. The structural refinement gave free R factors of R_1 = 8.10% and wR_2 = 24.09%. In checkcif, three level A alerts (PLAT972_ALERT_2_A) and four level B alerts (PLAT971_ALERT_2_B) were generated from residual density near the iodide ligands and Gd ions. This residual density could not be modelled as any chemically reasonable species, and it likely arises due to the strongly absorbing Gd or iodide ions or due to problems with the absorption correction. In addition, one level B alert (PLAT029_ALERT_3_B) was found due to low data completeness.

In order to resolve these issues, we analyzed crystals of **1-Gd** prepared using a different crystallization approach, as described in Section 1. In the second structure obtained from these crystals, the compound crystallized in the space group $P\overline{1}$ with one molecule in the asymmetric unit. Solvent mask, as implemented in Olex2 (analogous to SQUEEZE), was used to remove the electron density from the highly disordered solvent molecules in the lattice (V = 232 Å^3, 53 e$^-$). The structural refinement gave free R factors of R_1 = 4.74% and wR_2 = 13.27%. In checkcif, one level B alert (PLAT971_ALERT_2_B) was generated from residual density near the iodide ligands, likely due to the strongly absorbing iodide ions or due to problems with the absorption correction. For consistency with **1-Y**, we used the parameters from the first structure in the manuscript.

$[(Cp^{iPr5})_3Gd_3H_3I_2][B(C_6F_5)_4]$ (1-Gd^+). The compound crystallized in the space group *Cc* with one $[(Cp^{iPr5})_3Gd_3H_3I_2]^+$ cation and one $[B(C_6F_5)_4]^-$ anion in the asymmetric unit. Solvent mask, as implemented in Olex2 (analogous to SQUEEZE), was used to remove the electron density from the highly disordered solvent molecules in the lattice (V = 3102 Å^3, 1425 e$^-$). Two Cp^{iPr5} ligands showed positional disorder over two sites, and the occupancies of these two components were refined while constraining the sum to unity, yielding a ratio of 0.759(14):0.241(14) and 0.579(14):0.421(14), respectively. The hydride ligands near the Gd atoms were located manually

using several restraints (DFIX, SADI or FLAT). The application of the standard combination of SIMU, DELU restraints and EADP constraints to the displacement parameters of the cyclopentadienyl carbons and iodide ligands gave free R factors of R_1 = 4.34% and wR_2 = 11.45%. In checkcif, one level B alert (PLAT342_ALERT_3_B) was generated from the severe disorder within the Cp^{iPr5} ligands and the tetrakis(pentafluorophenyl)borate counter anion.

Table S1. Structure details for $(Cp^{iPr5})_2Ln_2(CH_2SiMe_3)_2I_2$ (Ln = Y, Gd).

	$\mathbf{(Cp^{iPr5})_2Y_2(CH_2SiMe_3)_2I_2}$	$\mathbf{(Cp^{iPr5})_2Gd_2(CH_2SiMe_3)_2I_2}$
Empirical formula	$C_{48}H_{92}I_2Si_2Y_2$	$C_{48}H_{92}Gd_2I_2Si_2$
Formula weight	1157.01	1293.69
Temperature/K	100.0	100.0
Crystal system	triclinic	triclinic
Space group	P-1	P-1
a/Å	10.3207(13)	9.6209(4)
b/Å	15.4296(19)	10.8612(4)
c/Å	19.605(3)	14.1447(6)
α/°	109.100(4)	72.2060(10)
β/°	104.733(4)	77.6510(10)
γ/°	94.813(4)	75.7750(10)
Volume/Å^3	2805.0(6)	1348.68(9)
Z	2	1
ρ_{calc}g/cm^3	1.370	1.5804
μ/mm^{-1}	3.229	3.655
F(000)	1184.0	642.0
Crystal size/mm^3	0.605 × 0.094 × 0.05	0.2 × 0.067 × 0.046
Radiation	MoKα (*λ* = 0.71073)	MoKα (*λ* = 0.71073)
2Θ range for data collection/°	4.15 to 50.7	3.058 to 50.696
Index ranges	$-12 \le h \le 12$<br>$-18 \le k \le 18$<br>$-23 \le l \le 23$	$-11 \le h \le 11$<br>$-13 \le k \le 13$<br>$-17 \le l \le 17$
Reflections collected	86448	38203
Independent reflections	10283 [R_{int} = 0.0754, R_{sigma} = 0.0424]	4938 [R_{int} = 0.0498, R_{sigma} = 0.0288]
Data/restraints/parameters	10283/909/683	4938/30/312
Goodness-of-fit on F^2	1.031	1.042
Final R indexes [I>=2σ (I)]	R_1 = 0.0338, wR_2 = 0.0710	R_1 = 0.0310, wR_2 = 0.0669
Final R indexes [all data]	R_1 = 0.0539, wR_2 = 0.0785	R_1 = 0.0389, wR_2 = 0.0707
Largest diff. peak/hole / e Å^{-3}	0.98/−0.44	2.20/−0.84

Table S2. Structure details for $(Cp^{iPr5})_3Ln_3H_3I_3$ (Ln = Y, Gd).

	**$(Cp^{iPr5})_3Y_3H_3I_3$**	**$(Cp^{iPr5})_3Gd_3H_3I_3$**
Empirical formula	$C_{60}H_{108}I_3Y_3$	$C_{60}H_{108}Gd_3I_3$
Formula weight	1476.89	1681.91
Temperature/K	296.15	100.0
Crystal system	monoclinic	monoclinic
Space group	$P2_1/n$	$P2_1/n$
a/Å	18.0363(8)	18.1407(14)
b/Å	17.1449(8)	17.1162(13)
c/Å	24.2130(11)	24.2503(19)
α/°	90	90
β/°	90.9060(10)	91.125(3)
γ/°	90	90
Volume/Å^3	7486.5(6)	7528.3(10)
Z	4	4
ρ_{calc}g/cm^3	1.310	1.484
μ/mm^{-1}	3.572	3.871
F(000)	2976.0	3276.0
Crystal size/mm^3	0.265 × 0.251 × 0.149	0.179 × 0.163 × 0.123
Radiation	MoKα (λ = 0.71073)	MoKα (λ = 0.71073)
2Θ range for data collection/°	3.278 to 50.746	3.272 to 50.696
Index ranges	$-21 \le h \le 21$<br>$-20 \le k \le 20$<br>$-29 \le l \le 29$	$-21 \le h \le 21$<br>$-20 \le k \le 20$<br>$-29 \le l \le 29$
Reflections collected	167640	155782
Independent reflections	13725 [R_{int} = 0.0740, R_{sigma} = 0.0349]	13757 [R_{int} = 0.0445, R_{sigma} = 0.0201]
Data/restraints/parameters	13725/7/701	13757/682/643
Goodness-of-fit on F^2	1.028	1.043
Final R indexes [I>=2σ (I)]	R_1 = 0.0484, wR_2 = 0.1264	R_1 = 0.0325, wR_2 = 0.0866
Final R indexes [all data]	R_1 = 0.0611, wR_2 = 0.1344	R_1 = 0.0379, wR_2 = 0.0905
Largest diff. peak/hole / e Å^{-3}	3.23/−1.69	2.80/−1.47

Table S3. Structure details for $(Cp^{iPr5})_3Y_3H_3I_2$ (**1-Y**) and $(Cp^{iPr5})_3Y_3D_3I_2$ (**$1\text{-}Y_D$**).

	**1-Y**	**$1\text{-}Y_D$**
Empirical formula	$C_{60}H_{108}I_2Y_3$	$C_{60}H_{105}D_3I_2Y_3$
Formula weight	1349.99	1353.01
Temperature/K	100	100
Crystal system	orthorhombic	orthorhombic
Space group	Pbca	Pbca
a/Å	24.3726(10)	24.3744(6)
b/Å	18.9432(8)	18.9626(5)
c/Å	27.6371(12)	27.6562(7)
α/°	90	90
β/°	90	90
γ/°	90	90
Volume/$Å^3$	12759.9(9)	12782.8(6)
Z	8	8
$\rho_{calc}g/cm^3$	1.405	1.406
μ/mm^{-1}	3.706	3.700
F(000)	5528.0	5528.0
Crystal size/mm^3	0.35 × 0.091 × 0.08	0.208 × 0.095 × 0.053
Radiation	MoKα (λ = 0.71073)	MoKα (λ = 0.71073)
2Θ range for data collection/°	3.342 to 50.7	3.094 to 50.7
Index ranges	$-29 \le h \le 27$<br>$-22 \le k \le 22$<br>$-33 \le l \le 33$	$-29 \le h \le 29$<br>$-21 \le k \le 22$<br>$-33 \le l \le 33$
Reflections collected	117323	107442
Independent reflections	11681 [R_{int} = 0.0953, R_{sigma} = 0.0506]	11706 [R_{int} = 0.0614, R_{sigma} = 0.0345]
Data/restraints/parameters	11681/0/598	11706/0/628
Goodness-of-fit on F^2	1.060	1.040
Final R indexes [I>=2σ (I)]	R_1 = 0.0531, wR_2 = 0.1223	R_1 = 0.0519, wR_2 = 0.1367
Final R indexes [all data]	R_1 = 0.0758, wR_2 = 0.1350	R_1 = 0.0671, wR_2 = 0.1459
Largest diff. peak/hole / e $Å^{-3}$	2.80/−1.41	2.24/−3.23

Table S4. Structure details for $(Cp^{iPr5})_3Gd_3H_3I_2$ (**1-Gd**).

	**Structure 1**	**Structure 2**
Empirical formula	$C_{60}H_{108}Gd_3I_2$	$C_{60}H_{108}Gd_3I_2$
Formula weight	1555.01	1555.01
Temperature/K	100	100
Crystal system	orthorhombic	triclinic
**Space group**	**Pbca**	**P-1**
a/Å	24.556(5)	14.9765(2)
b/Å	18.937(4)	15.0481(2)
c/Å	27.772(6)	18.0252(2)
α/°	90	83.4540(10)
β/°	90	85.0350(10)
γ/°	90	60.589(2)
Volume/Å3	12915(4)	3513.49(10)
Z	8	2
ρcalcg/cm3	1.600	1.470
μ/mm^{-1}	27.371	25.152
F(000)	6128.0	1532.0
Crystal size/mm^3	0.1 × 0.02 × 0.01	0.1 × 0.08 × 0.06
Radiation	CuKα (λ = 1.54178)	Cu Kα (λ = 1.54184)
2Θ range for data collection/°	6.364 to 130.168	6.766 to 160.906
Index ranges	−21 ≤ h ≤ 28<br>−22 ≤ k ≤ 18<br>−32 ≤ l ≤ 27	−18 ≤ h ≤ 19<br>−18 ≤ k ≤ 19<br>−23 ≤ l ≤ 22
Reflections collected	47893	104473
Independent reflections	10527 [R_{int} = 0.0985, R_{sigma} = 0.0720]	14945 [R_{int} = 0.0869, R_{sigma} = 0.0400]
Data/restraints/parameters	10527/12/628	14945/945/764
Goodness-of-fit on F2	1.043	1.094
Final R indexes [I>=2σ (I)]	R1 = 0.0810, wR2 = 0.2203	R1 = 0.0474, wR2 = 0.1243
Final R indexes [all data]	R1 = 0.1062, wR2 = 0.2409	R1 = 0.0557, wR2 = 0.1328
Largest diff. peak/hole / e $Å^{-3}$	3.57/−2.94	2.89/−1.81

Table S5. Structure details for [(Cp^{iPr5})$_3$Gd$_3$H$_3$I$_2$][B(C$_6$F$_5$)$_4$] (**1-Gd$^+$**).

Empirical formula	$C_{84}H_{108}BF_{20}Gd_3I_2$
Formula weight	2234.06
Temperature/K	100.0
Crystal system	monoclinic
Space group	Cc
a/Å	14.8679(10)
b/Å	25.5832(16)
c/Å	29.237(2)
α/°	90
β/°	102.513(2)
γ/°	90
Volume/Å^3	10856.8(13)
Z	4
ρ_{calc}g/cm^3	1.3636
μ/mm^{-1}	2.447
F(000)	4380.0
Crystal size/mm^3	0.426 × 0.201 × 0.082
Radiation	Mo Kα (*λ* = 0.71073)
2Θ range for data collection/°	3.272 to 50.698
Index ranges	$-17 \leq h \leq 17$<br>$-30 \leq k \leq 30$<br>$-35 \leq l \leq 35$
Reflections collected	119665
Independent reflections	19759 [R_{int} = 0.0457, R_{sigma} = 0.0361]
Data/restraints/parameters	19759/1208/1116
Goodness-of-fit on F^2	1.049
Final R indexes [I>=2σ (I)]	R_1 = 0.0434, wR_2 = 0.1102
Final R indexes [all data]	R_1 = 0.0493, wR_2 = 0.1145
Largest diff. peak/hole / e Å^{-3}	0.98/−1.14
Flack parameter	0.131(13)

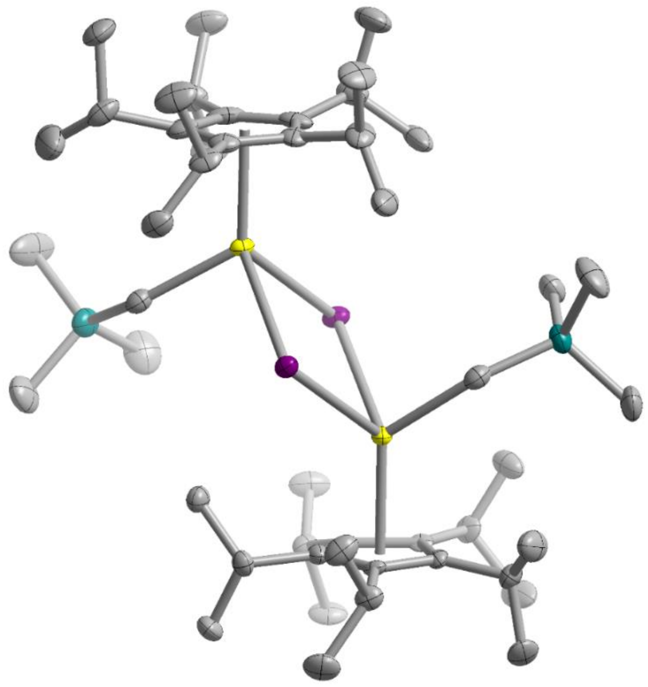

Figure S12. Solid-state structure of $(Cp^{iPr5})_2Y_2(CH_2SiMe_3)_2I_2$ with thermal ellipsoids at the 50% probability level. Yellow, purple, turquoise, and grey ellipsoids represent Y, I, Si, and C atoms, respectively. Hydrogen atoms are omitted for clarity.

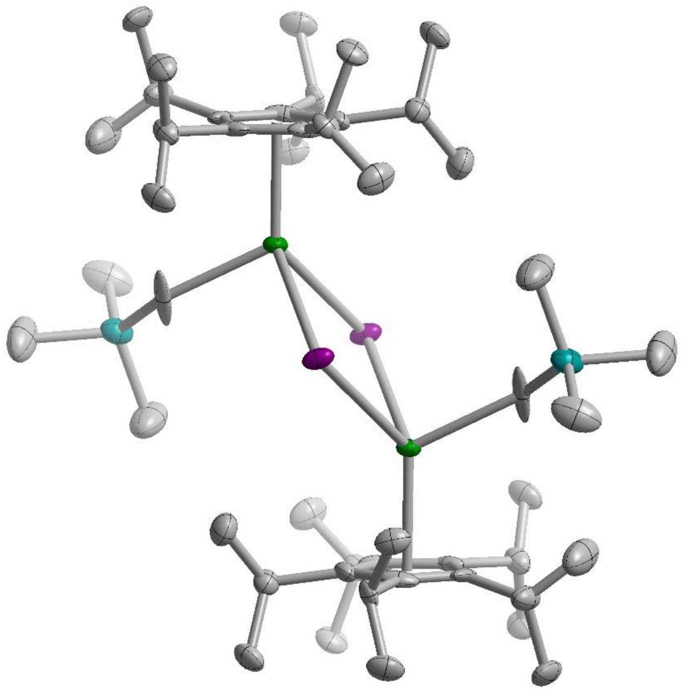

Figure S13. Solid-state structure of $(Cp^{iPr5})_2Gd_2(CH_2SiMe_3)_2I_2$ with thermal ellipsoids at the 50% probability level. Green, purple, turquoise, and grey ellipsoids represent Gd, I, Si, and C atoms, respectively. Hydrogen atoms are omitted for clarity.

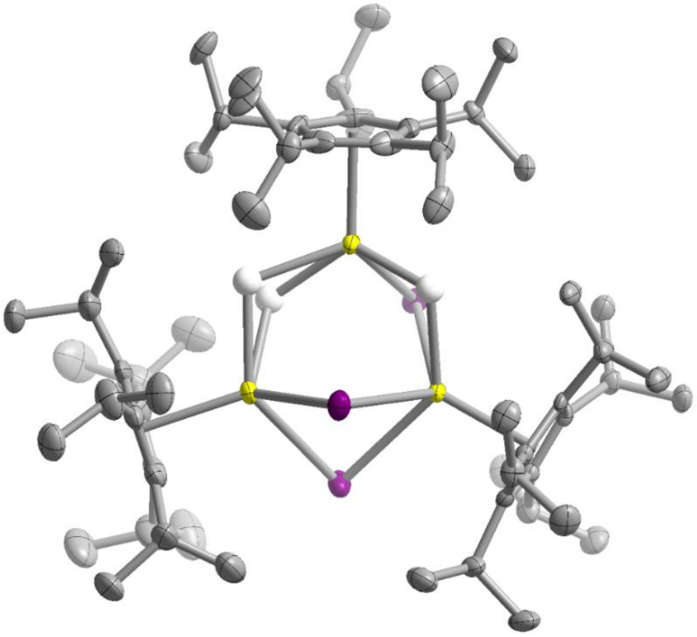

Figure S14. Solid-state structure of $(Cp^{iPr5})_3Y_3H_3I_3$ with thermal ellipsoids at the 50% probability level. Yellow, purple, and grey ellipsoids represent Y, I, and C atoms, respectively. Hydride ligands bound to Y are indicated with white spheres. Hydrogen atoms and the positional disorder of the Cp^{iPr5} ligand are omitted for clarity.

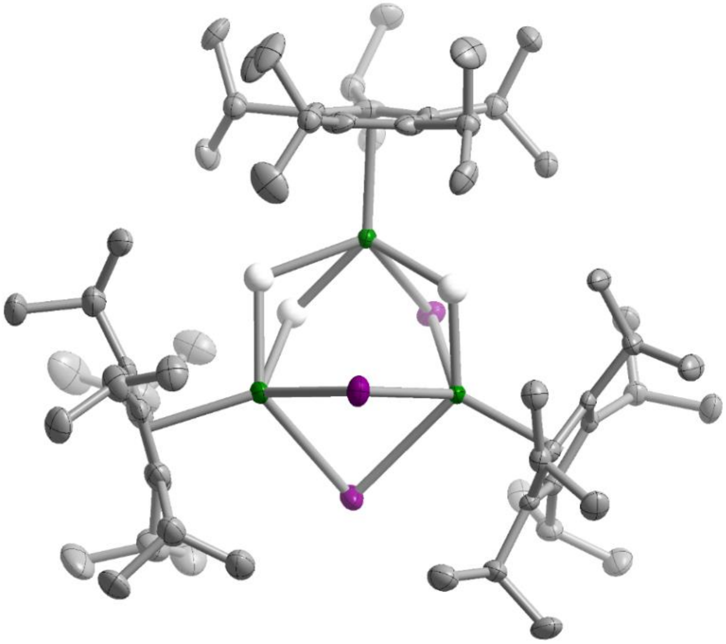

Figure S15. Solid-state structure of $(Cp^{iPr5})_3Gd_3H_3I_3$ with thermal ellipsoids at the 50% probability level. Green, purple, and grey ellipsoids represent Gd, I, and C atoms, respectively. Hydride ligands bound to Gd are indicated with white spheres. Hydrogen atoms and positional disorder of the Cp^{iPr5} ligand are omitted for clarity

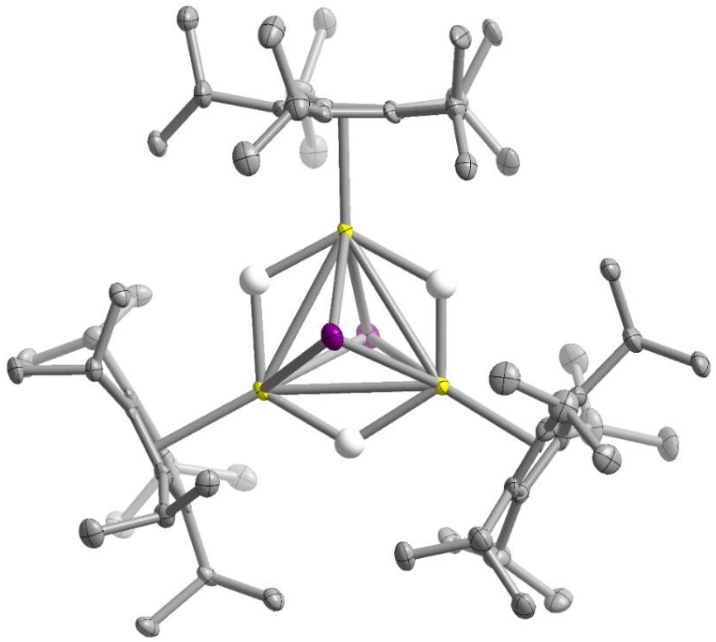

Figure S16. Solid-state structure of **1-Y** with thermal ellipsoids at the 50% probability level. Yellow, purple, and grey ellipsoids represent Y, I, and C atoms, respectively. Hydride ligands bound to Y are indicated with white spheres. Hydrogen atoms are omitted for clarity

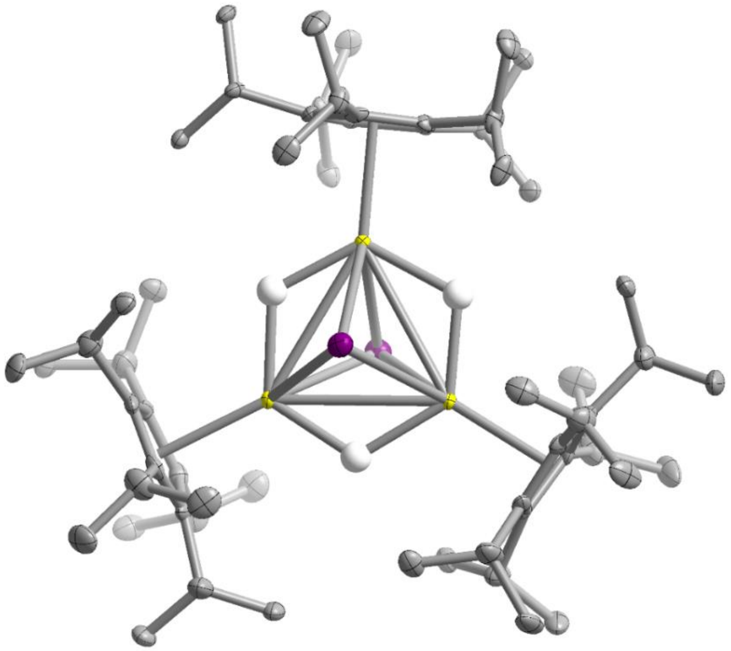

Figure S17. Solid-state structure of **1-Y$_D$** with thermal ellipsoids at the 50% probability level. Yellow, purple, and grey ellipsoids represent Y, I, and C atoms, respectively. Deuteride ligands bound to Y are indicated with white spheres. Hydrogen atoms are omitted for clarity.

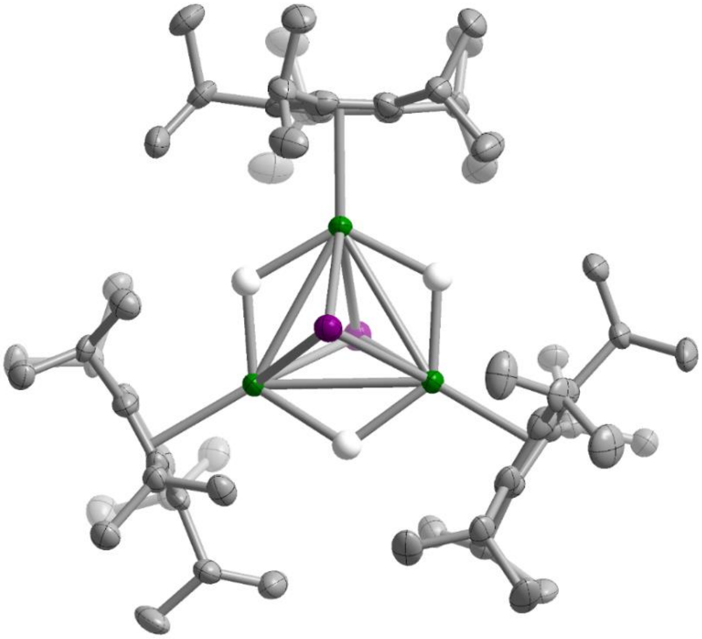

Figure S18. Solid-state structure of **1-Gd** with thermal ellipsoids at the 50% probability level. Green, purple, and grey ellipsoids represent Gd, I, and C atoms, respectively. Hydride ligands bound to Gd are indicated with white spheres. Hydrogen atoms are omitted for clarity.

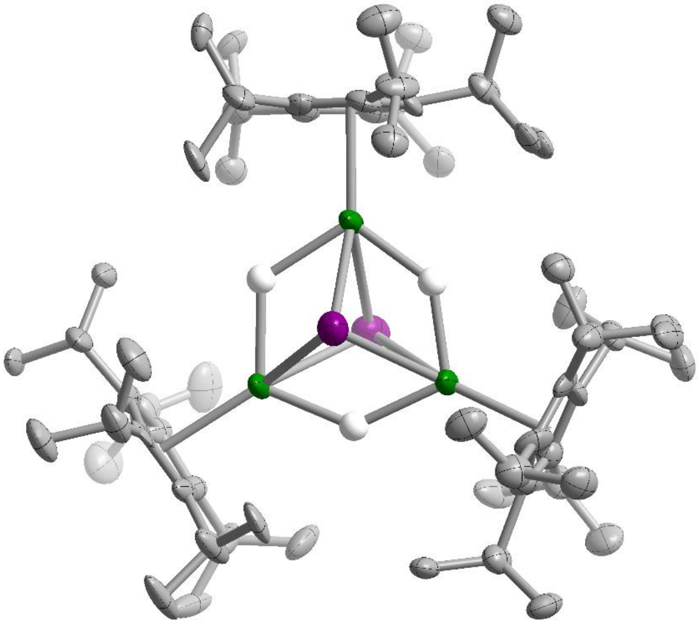

Figure S19. Solid-state structure of **1-Gd$^+$** with thermal ellipsoids at the 50% probability level. Green, purple, and grey ellipsoids represent Gd, I, and C atoms, respectively. Hydride ligands bound to Gd are indicated with white spheres. Hydrogen atoms, positional disorder of the Cp^{iPr5} ligand, and the $B(C_6F_5)_4$ anion are omitted for clarity.

Table S6. Selected bond distances (Å) for **1-Gd**, **1-Gd$^+$**, **1-Y**, $(Cp^{iPr5})_2Gd_2I_3$ and $(Cp^{iPr5})_2Y_2I_3$[2].

		**1-Gd**	**1-Gd$^+$**	**1-Y**	$(Cp^{iPr5})_2Gd_2I_3$	$(Cp^{iPr5})_2Y_2I_3$
Ln1	Ln2	3.6023(12)	3.7858(8)	3.4865(8)		
Ln2	Ln3	3.5613(11)	3.8042(8)	3.5194(8)		
Ln3	Ln1	3.5952(11)	3.7717(8)	3.5189(8)		
Ln···Ln (avg)		3.5863(7)[a]	3.7872(4)[a]	3.5083(5)[a]	3.769(1)	3.727(1)
Ln1	I1	3.1555(12)	3.1694(11)	3.1435(7)		
Ln1	I2	3.1995(12)	3.1624(12)	3.1720(7)		
Ln2	I1	3.2099(12)	3.1714(11)	3.1725(7)		
Ln2	I2	3.1500(12)	3.1622(12)	3.1323(7)		
Ln3	I1	3.1752(13)	3.1843(11)	3.1241(7)		
Ln3	I2	3.1917(13)	3.1745(10)	3.1825(7)		
Ln−I (avg)		3.1803(5)[a]	3.1707(5)[a]	3.1545(3)[a]	3.060(1)	3.023(1)
Ln1	C1	2.693(12)	2.661(10)	2.655(6)		
Ln1	C2	2.740(11)	2.616(10)	2.670(6)		
Ln1	C3	2.716(11)	2.604(11)	2.644(6)		
Ln1	C4	2.689(12)	2.646(12)	2.631(6)		
Ln1	C5	2.670(12)	2.667(12)	2.631(6)		
Ln2	C21	2.630(11)	2.618(11)	2.606(6)		
Ln2	C22	2.673(13)	2.593(11)	2.643(6)		
Ln2	C23	2.714(12)	2.650(10)	2.705(6)		
Ln2	C24	2.716(12)	2.666(11)	2.683(6)		
Ln2	C25	2.676(12)	2.627(10)	2.632(6)		
Ln3	C41	2.681(12)	2.625(11)	2.691(6)		
Ln3	C42	2.701(10)	2.635(9)	2.642(6)		
Ln3	C43	2.701(10)	2.657(10)	2.617(6)		
Ln3	C44	2.685(10)	2.678(10)	2.664(6)		
Ln3	C45	2.677(11)	2.626(11)	2.692(6)		
Ln−C (avg)		2.6908(3)[a]	2.638(3)[a]	2.654(2)[a]	2.668(13)	2.628(2)
Ln1	centroid	2.4161(8)	2.3461(5)	2.3528(6)		
Ln2	centroid	2.3963(8)	2.3483(5)	2.3620(5)		
Ln3	centroid	2.3997(7)	2.3476(5)	2.3706(5)		
Ln−$Cp_{centroid}$ (avg)		2.4040(4)[a]	2.3473(3)[a]	2.3618(3)[a]	2.375(16)	2.337(3)

[a] The standard deviation of the average value was estimated from $\sigma = \sqrt{\sum \sigma i2} / N$, where σi is the standard deviation of each bond distance i and N is the number of distances averaged.

4. Cyclic Voltammetry Data for 1-Gd$^+$

Cyclic voltammograms were recorded using a Bio-Logic SP-200 potentiostat and a three-electrode setup, with Pt working electrode, glassy carbon auxiliary electrode, and Ag wire reference electrode. Data were collected on 2 mM solutions of **1-Gd$^+$** in 1,2-difluorobenzene with tetrabutylammonium tetrakis(pentafluorophenyl)borate ([nBu$_4$][B(C$_6$F$_5$)$_4$]) as electrolyte (0.5 M) at a scan rate of 100 mV/s. Potentials were referenced to the [FeCp$_2$]$^{+/0}$ redox couple.

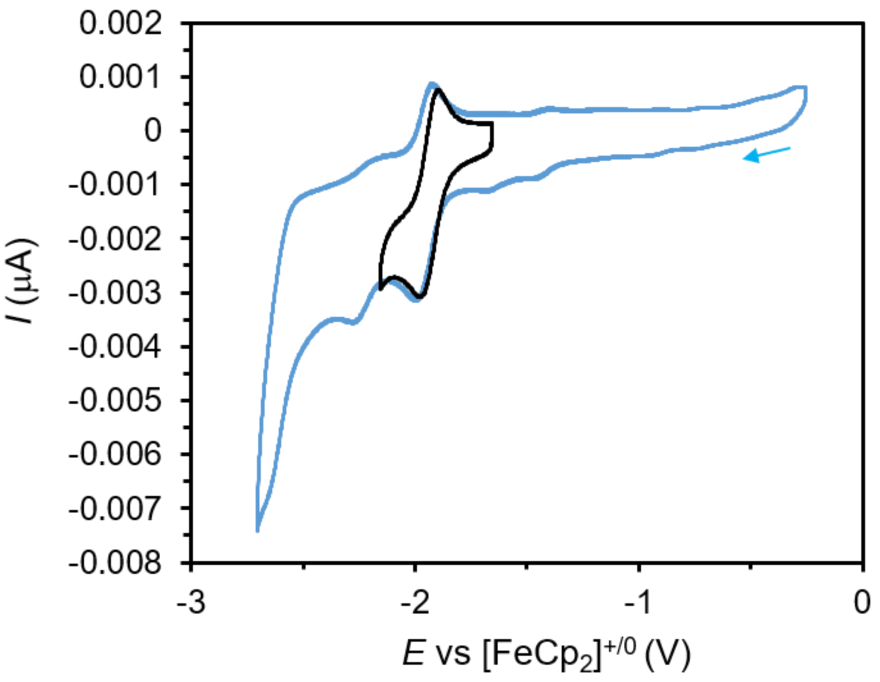

Figure S20. Cyclic voltammogram of **1-Gd$^+$**. The arrow represents the starting point and the direction of the scan.

5. UV-Vis-NIR Spectra for 1-Y, 1-Gd, and 1-Gd⁺

UV-Vis-NIR spectra were collected on solutions of **1-Ln** (Ln = Y, Gd) in n-hexane and **1-Gd⁺** in 1,2-difluorobenzene. Plots of concentration versus absorbance were used to extract extinction coefficients for each of the features observed in the spectra. Diffuse reflectance spectra were collected for polycrystalline samples of **1-Ln** (Ln = Y, Gd) and **1-Gd⁺**, which were diluted in $BaSO_4$ and ground with a mortar and pestle to produce a homogenous powder.

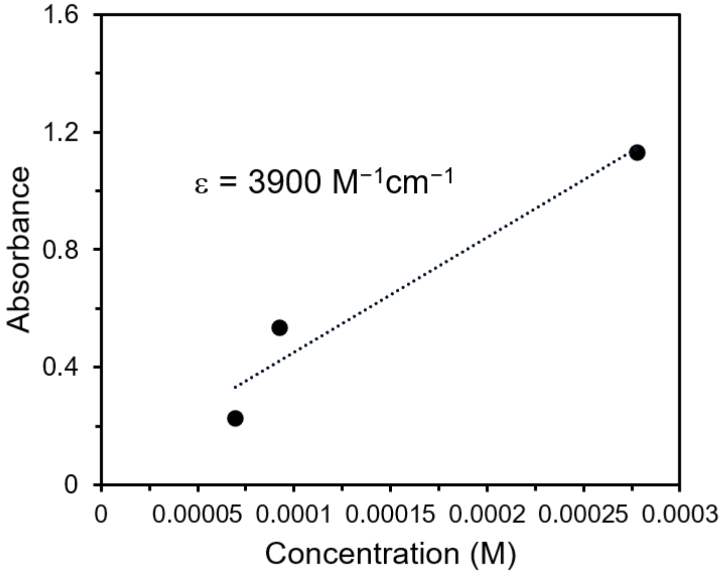

Figure S21. Plot of absorbance versus concentration for the feature at 831 nm in the UV-Vis-NIR spectrum of **1-Y**. Black points represent experimental data and the black line represents the fit to the data used to extract the extinction coefficient.

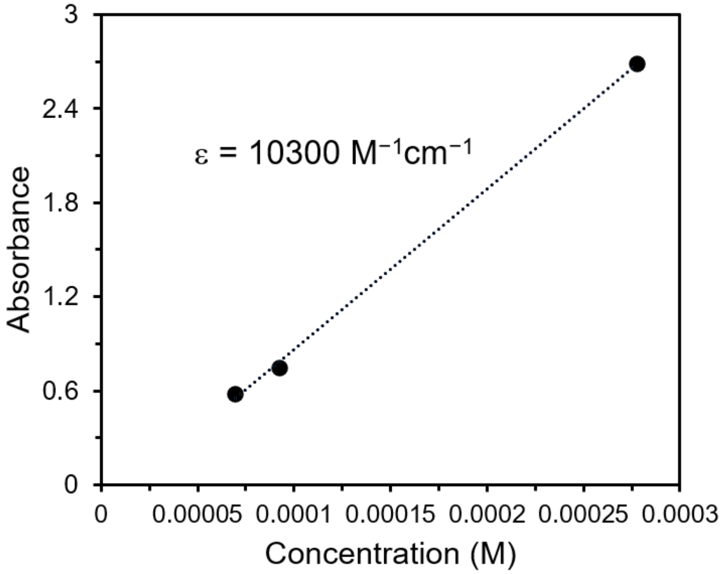

Figure S22. Plot of absorbance versus concentration for the feature at 319 nm in the UV-Vis-NIR spectrum of **1-Y**. Black points represent experimental data and the black line represents the fit to the data used to extract the extinction coefficient.

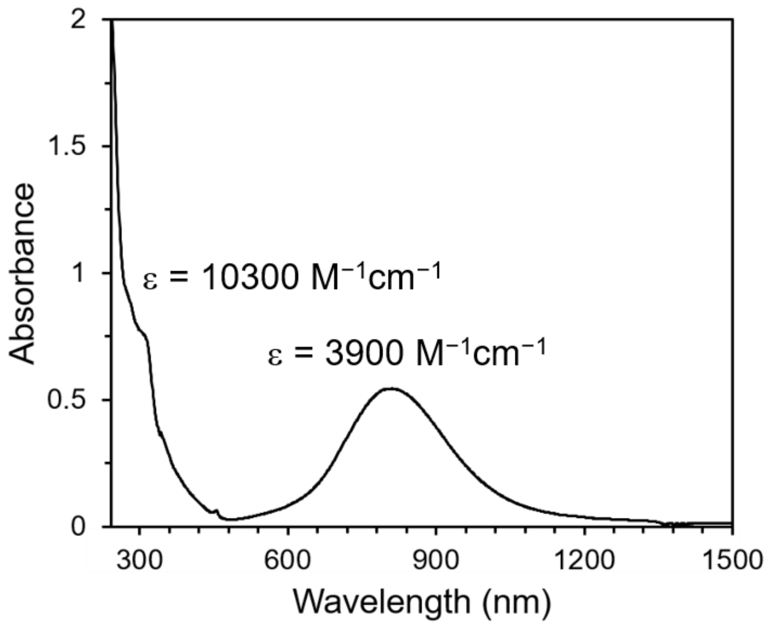

Figure S1. UV-Vis-NIR spectrum of **1-Y**.

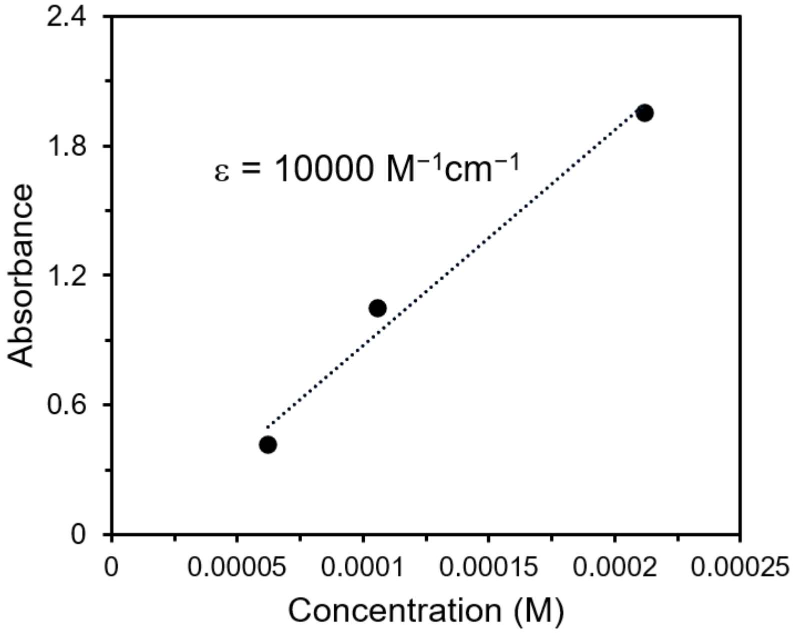

Figure S24. Plot of absorbance versus concentration for the feature at 313 nm in the UV-Vis-NIR spectrum of **1-Gd**. Black points represent experimental data and the black line represents the fit to the data used to extract the extinction coefficient.

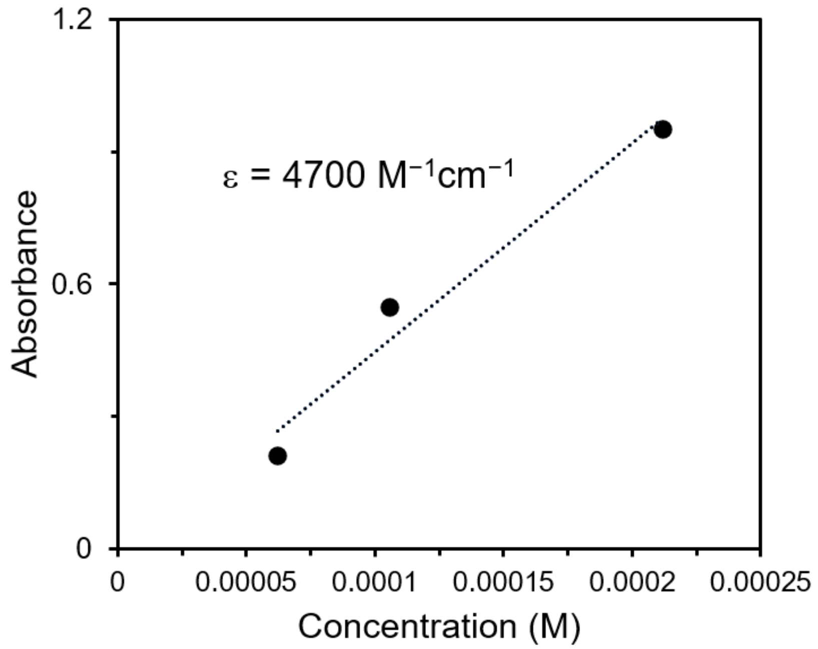

Figure S25. Plot of absorbance versus concentration for the feature at 358 nm in the UV-Vis-NIR spectrum of **1-Gd**. Black points represent experimental data and the black line represents the fit to the data used to extract the extinction coefficient.

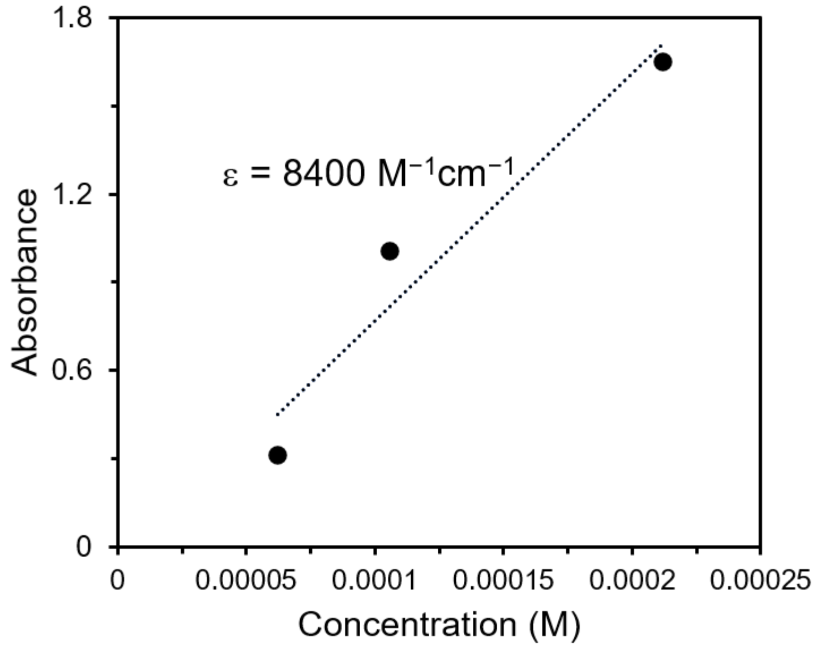

Figure S26. Plot of absorbance versus concentration for the feature at 853 nm in the UV-Vis-NIR spectrum of **1-Gd**. Black points represent experimental data and the black line represents the fit to the data used to extract the extinction coefficient.

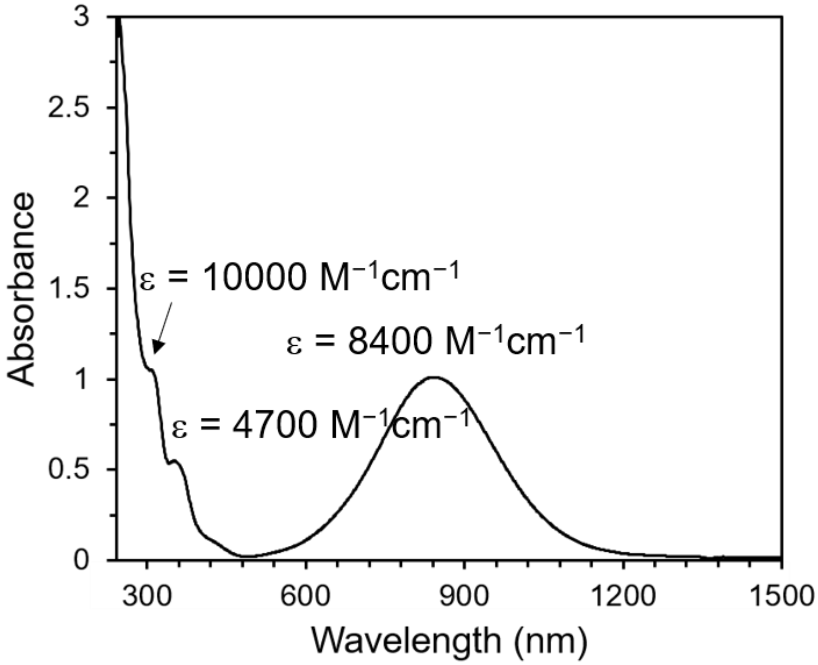

Figure S27. UV-Vis-NIR spectrum of **1-Gd**.

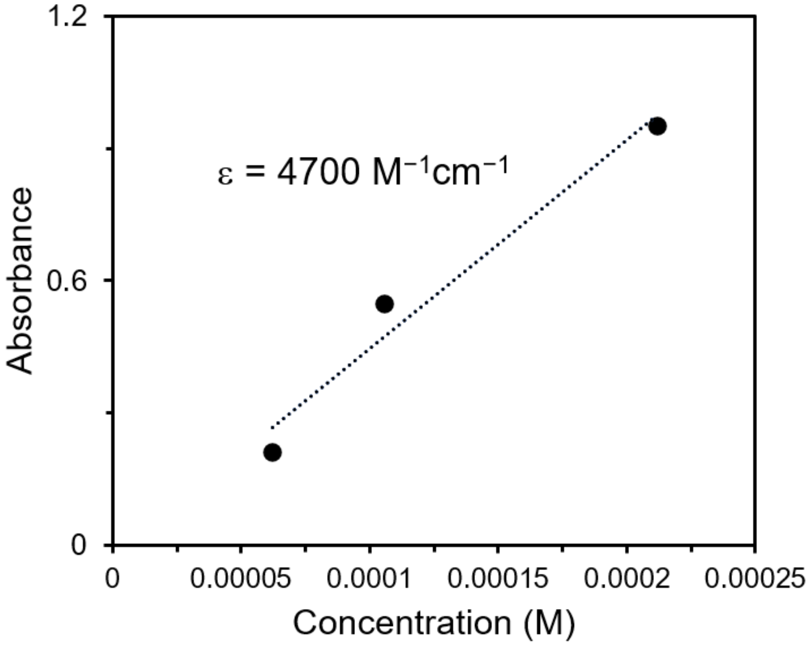

Figure S28. Plot of absorbance versus concentration for the feature at 358 nm in the UV-Vis-NIR spectrum of **1-Gd**$^{+}$. Black points represent experimental data and the black line represents the fit to the data used to extract the extinction coefficient.

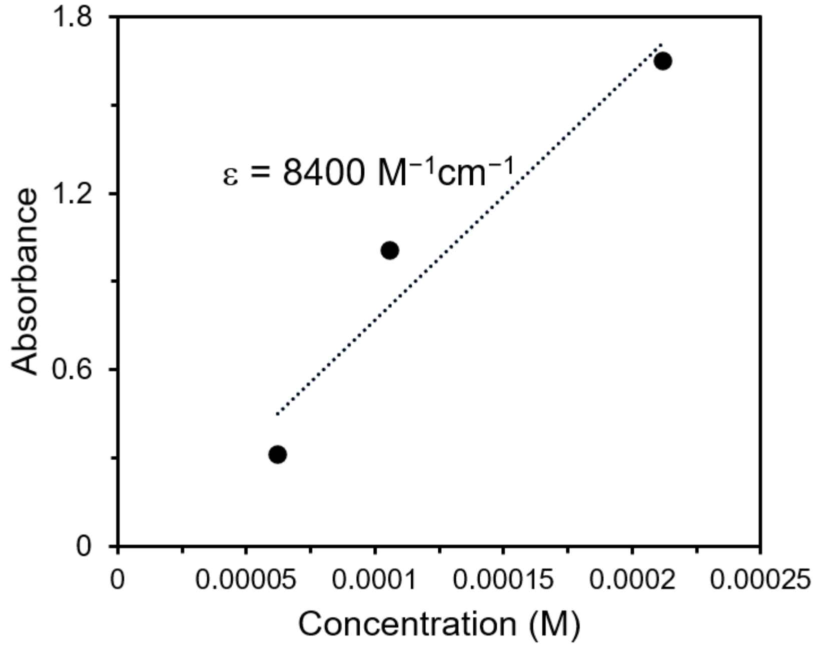

Figure S29. Plot of absorbance versus concentration for the feature at 853 nm in the UV-Vis-NIR spectrum of **1-Gd**$^{+}$. Black points represent experimental data and the black line represents the fit to the data used to extract the extinction coefficient.

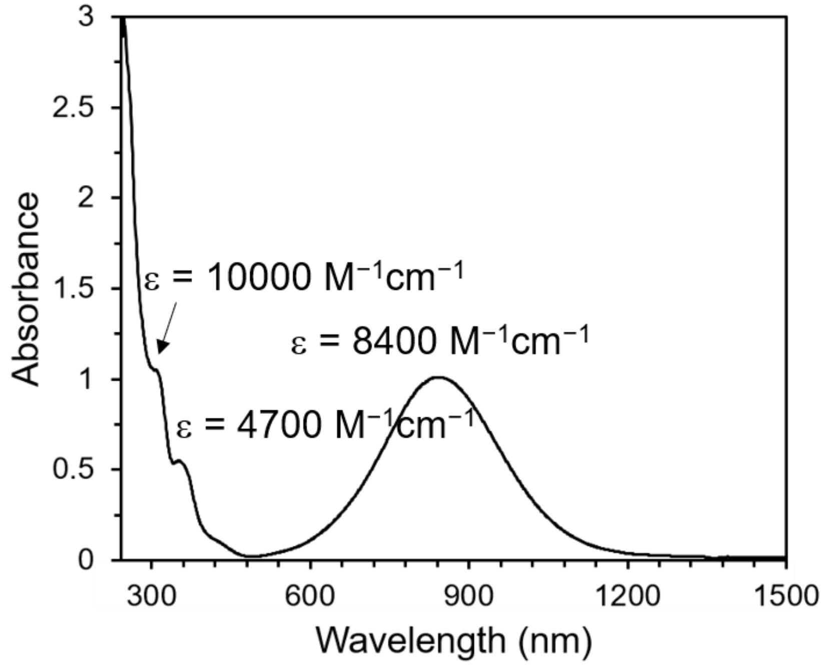

Figure S30. UV-Vis-NIR spectrum of **1-Gd**$^{+}$.

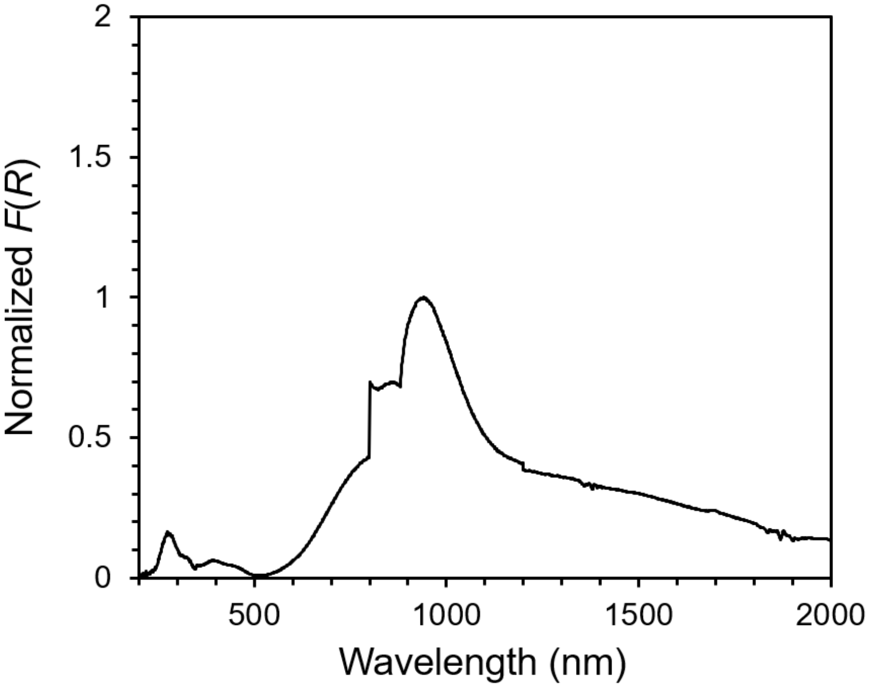

Figure S2 Diffuse reflectance spectrum of **1-Y**. The value of the Kubelka-Munk transformation of the raw diffuse reflectance, $F(R)$, was normalized with the peak absorbance at 279 nm set to $F(R) = 1$ to facilitate comparisons.

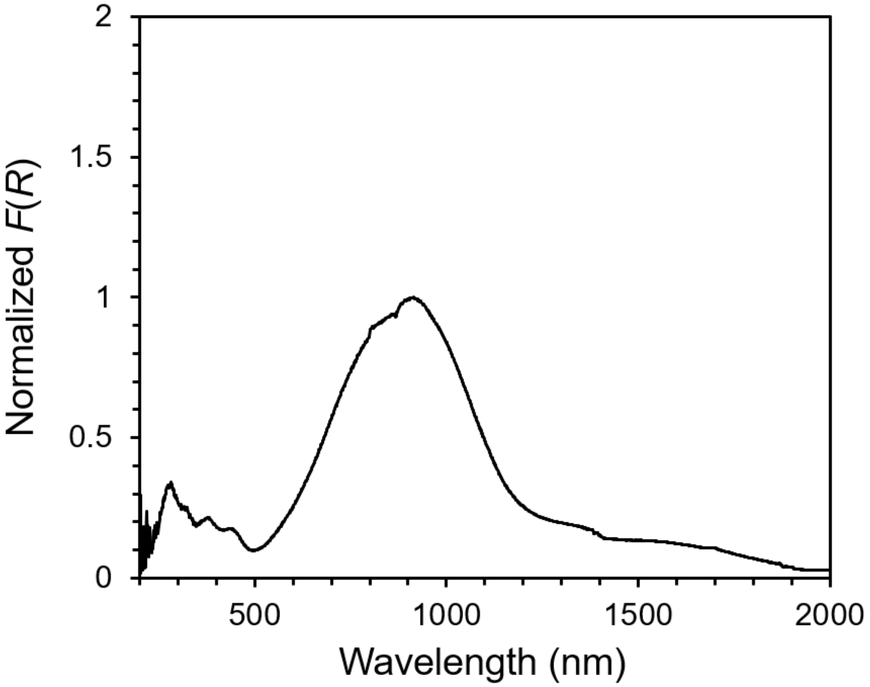

Figure S3 Diffuse reflectance spectrum of **1-Gd**. The value of the Kubelka-Munk transformation of the raw diffuse reflectance, $F(R)$, was normalized with the peak absorbance at 279 nm set to $F(R) = 1$ to facilitate comparisons.

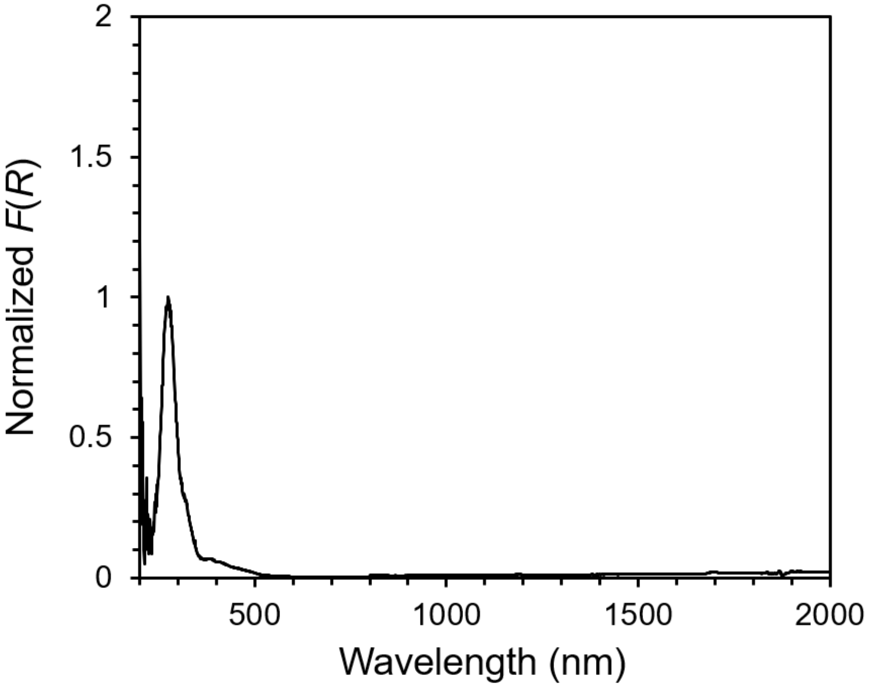

Figure S4 Diffuse reflectance spectrum of **1-Gd**$^{+}$. The value of the Kubelka-Munk transformation of the raw diffuse reflectance, $F(R)$, was normalized with the peak absorbance at 279 nm set to $F(R) = 1$ to facilitate comparisons.

6. Magnetization and Dc Magnetic Susceptibility Data for 1-Gd and 1-Gd$^+$

Using a Quantum Design MPMS2 SQUID magnetometer, dc magnetic susceptibility data were collected at temperatures ranging from 2 to 300 K under applied fields of 1, 5, and 10 kOe for **1-Gd** and **1-Gd$^+$**. Magnetic samples were prepared by adding polycrystalline powder (19.0 mg for **1-Gd**, 8.2 mg for **1-Gd$^+$**) to a 5 mm i.d./7 mm o.d. quartz tube with a raised quartz platform. A layer of solid eicosane was added on top of each sample (19.0 mg for **1-Gd**, 20.2 mg for **1-Gd$^+$**). The tubes were then fitted with Teflon sealable adapters, evacuated using a glovebox vacuum pump, and removed from the glovebox. The portion of the tube containing the sample was cooled quickly in liquid nitrogen, and the tube was flame sealed with an O_2/H_2 flame. After flame-sealing, the eicosane was melted in a 45 °C water bath in order to provide good thermal contact and to prevent crystallite torqueing. All data were corrected for diamagnetic contributions from the core diamagnetism of the sample and eicosane, estimated using Pascal's constants: $\chi_{\mathrm{dia}} = -0.00082865$ emu/mol for **1-Gd**, −0.00111744 emu/mol for **1-Gd$^+$**, and 0.00024306 emu/mol for eicosane.

The dc susceptibility data obtained for **1-Gd** and **1-Gd$^+$** were fit with the program PHI.[6] Fits to the dc susceptibility data for **1-Gd** were performed using the Hamiltonian:

$$\hat{H} = -2J\hat{R} \cdot (\hat{S}_{\mathrm{Gd1}} + \hat{S}_{\mathrm{Gd2}} + \hat{S}_{\mathrm{Gd3}}) + \mu_B g(\hat{S}_{\mathrm{Gd1}} + \hat{S}_{\mathrm{Gd2}} + \hat{S}_{\mathrm{Gd3}} + \hat{R}) \cdot \vec{B} \quad \text{(S1)}$$

where $\hat{S}_{\mathrm{Gd1}}$, $\hat{S}_{\mathrm{Gd2}}$, and $\hat{S}_{\mathrm{Gd3}}$ are the spin operators for each metal site in the trinuclear complex and $\hat{R}$ is the spin operator for the σ-bonding electron, and J is the spin-spin coupling constant.

We have not included Gd–Gd exchange coupling, as there is no sensitivity in the experimental data to fit this value. The only datum we have is the slope of $\chi_{\mathrm{M}}T$ at high temperatures, and here Gd–Gd and 4f–σ parameters are correlated. Thus, we have focused on estimating the strength of the most significant term.

For **1-Gd$^+$**, the data were fit using the Hamiltonian

$$\hat{H} = -2J(\hat{S}_{\mathrm{Gd1}} \cdot \hat{S}_{\mathrm{Gd2}} + \hat{S}_{\mathrm{Gd2}} \cdot \hat{S}_{\mathrm{Gd3}} + \hat{S}_{\mathrm{Gd3}} \cdot \hat{S}_{\mathrm{Gd1}}) + \mu_B g(\hat{S}_{\mathrm{Gd1}} + \hat{S}_{\mathrm{Gd2}} + \hat{S}_{\mathrm{Gd3}}) \cdot \vec{B} \quad \text{(S2)}$$

where $\hat{S}_{\mathrm{Gd1}}$, $\hat{S}_{\mathrm{Gd2}}$, and $\hat{S}_{\mathrm{Gd3}}$ are the spin operators for each metal site and J represents the magnetic exchange coupling between Gd^{3+} ions.

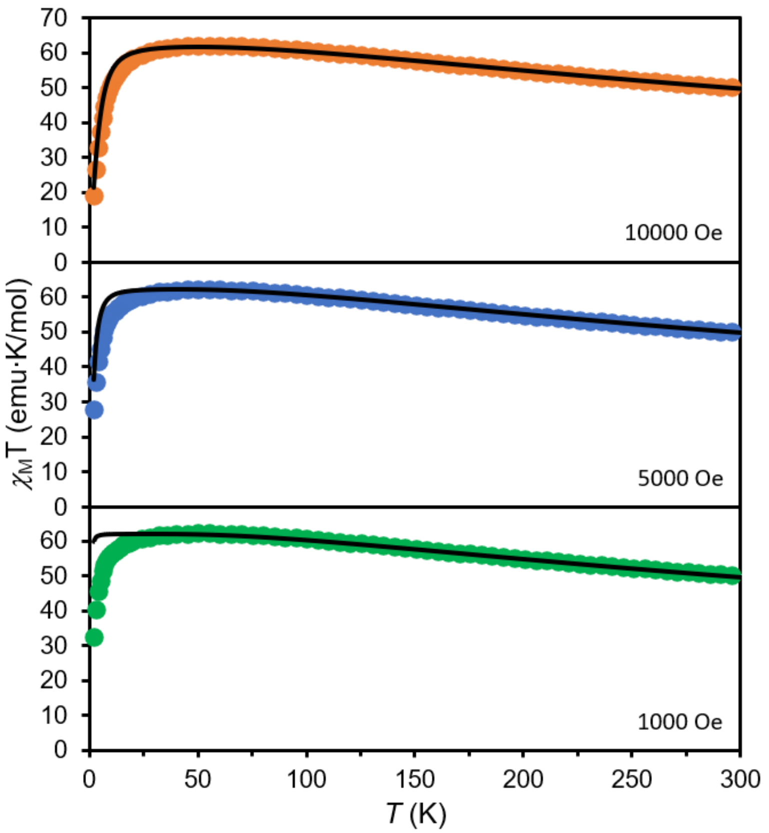

Figure S34. Zero-field-cooled dc magnetic susceptibility data for **1-Gd** under applied fields of 1, 5, and 10 kOe. Black lines represent fits to the data, as described above.

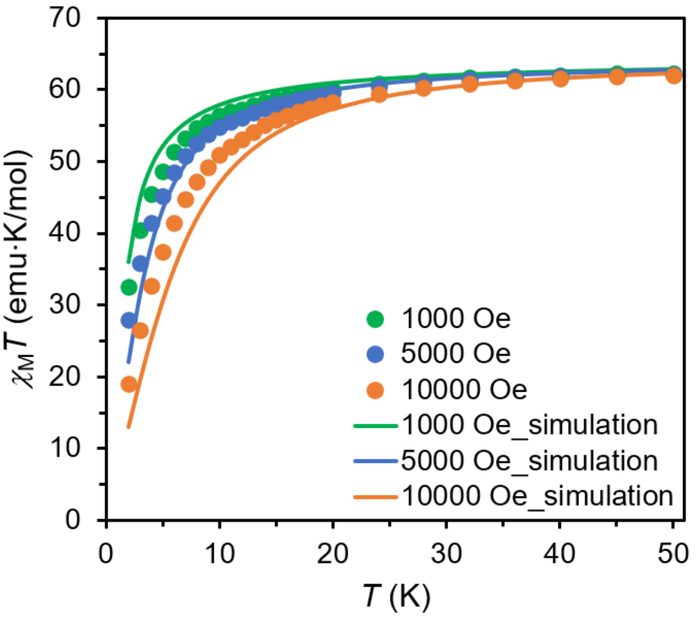

Figure S35. Magnetic susceptibility data for **1-Gd** in the low temperature region (2 to 50 K). The solid lines correspond to simulations of the data assuming population of only an $S = 11$ state with $g = 1.97$, zero field splitting ($D = 4.2(2)$ cm^{-1}), and Zeeman splitting using PHI.[6]

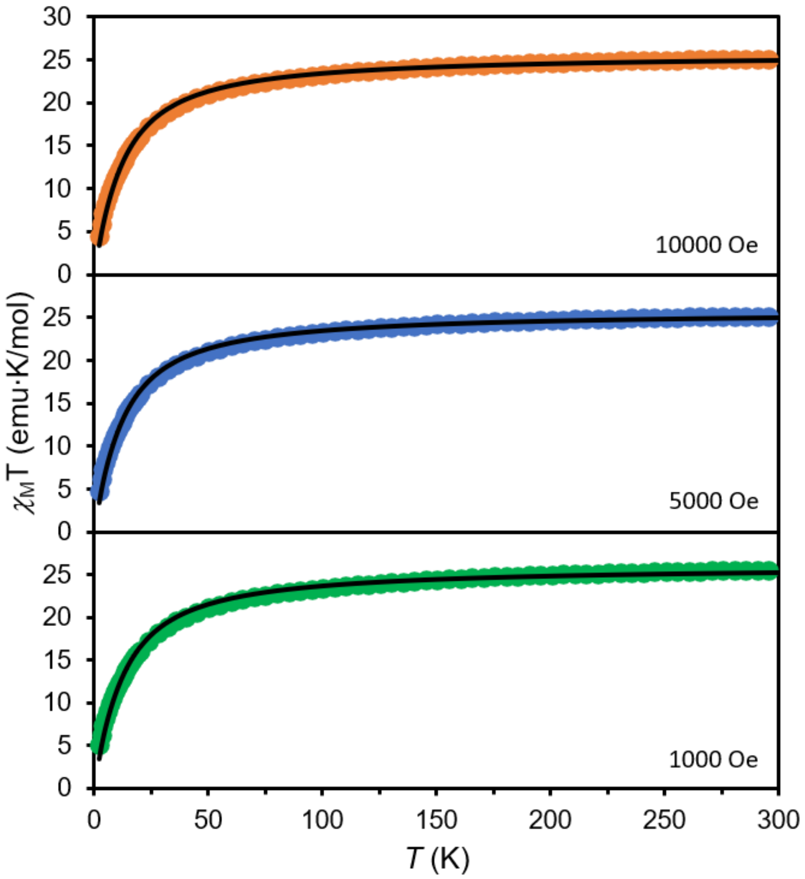

Figure S36. Zero-field-cooled dc magnetic susceptibility data for **1-Gd**$^+$ under applied fields of 1, 5, and 10 kOe. Black lines represent fits to the data, as described above.

Table S7. Parameters used to fit the dc susceptibility data for **1-Gd** and **1-Gd**$^+$.

	**Field (T)**	***g***	***J***
**1-Gd**	0.1, 0.5, and 1	1.94(1)	168.5(4)
**1-Gd**$^+$	0.1	2.09(1)	$-3.16(4) \times 10^{-1}$
	0.5 and 1	2.08(1)	$-3.09(2) \times 10^{-1}$

7. EPR Spectroscopy Data for 1-Y and 1-Y_D

7.1 Sample Preparations and Instrument Details

EPR samples were prepared from solutions of $(Cp^{iPr5})_3Y_3H_3I_2$ (**1-Y**) or $(Cp^{iPr5})_3Y_3D_3I_2$ (**1-Y_D**) dissolved in methylcyclohexane solution (ca. 1 mM). The sample used for X-band and Q-band measurements was prepared as follows: the solution was loaded into a quartz tube (o.d. 2.40 mm, i.d. 2.00 mm) inside an Ar-filled glovebox, capped with a rubber septum, and removed from the glove box where the sample was immersed in liquid nitrogen. The sample was then flame sealed under static reduced pressure. The D-band EPR sample was prepared by loading the solution sample into a quartz tube (o.d. 0.600 mm, i.d. 0.500 mm) inside of an Ar-filled glovebox. The sample tube was put inside a 2 mL cryovial, which was capped, then was taken out of the glovebox and immediately submerged in liquid nitrogen. The cryovial was kept submerged in liquid N_2 for ~2 h while being transported for measurement (note the cryovial is not airtight). The sample was then removed from the cryovial and directly exposed to air while being loaded into the spectrometer, which had pre-cooled at 90 K under a flow of helium. The sample was kept frozen throughout the whole process.

Note, EPR spectra were not collected for **1-Gd**. The first reason for this is that the compound has a non-Kramers ground state and is therefore not necessarily EPR active. For this reason, **1-Y** offers a simpler way of studying electron delocalization using EPR spectroscopy. Second, even though EPR signal might be detected, the distribution of zero-field splitting interaction for Gd ions tends to broaden EPR spectra (at high frequencies), obscuring important features, or gives rise to multiple splittings (at X-band frequency), which can be very complex. Lastly, given that the majority (70%) of the isotopes of Gd nuclei are non-magnetic, the use of hyperfine spectroscopies (HYSCORE, ENDOR, EDNMR spectroscopies) to determine the degree of electron delocalization is much more challenging and the results are not as useful as those obtained for **1-Y**.

EPR spectra were recorded at the CalEPR center in the Department of Chemistry, University of California, Davis. Continuous wave (CW) X-Band (9.39 GHz) spectra were collected using a Bruker Biospin EleXsys E500 spectrometer (Billerica, MA) equipped with a super high Q resonator (ER4122SHQE). Cryogenic temperatures were controlled and maintained through the use of an ESR900 liquid helium cryostat in conjunction with a temperature controller (Oxford Instruments ITC503) and a gas flow controller. Other spectrometer settings are given in the corresponding figure captions.

Echo-detected field sweep spectra at Q-band (34 GHz) were collected using a Bruker Biospin EleXsys E580 spectrometer equipped with a 10 W amplifier and an R. A. Isaacson-built cylindrical TE011 resonator mounted in an Oxford CF935 cryostat. The standard Hahn echo sequence ($\pi/2 - \tau - \pi -$ echo) was applied to each sample via the XEPR software at 24 K and with $\pi/2$ = 16 ns and τ = 200 ns at various magnetic field values. HYSCORE spectra were collected using a four-pulse sequence ($\pi/2 - \tau - \pi/2 - t_1 - \pi - t_2 - \pi/2 - \tau -$ echo) at 20 K ($\pi/2$ = 16 ns, τ = 300 ns). ENDOR measurements were performed at 20 K by employing the Mims pulse sequence: π – RF $- \pi/2 - \tau - \pi - \tau -$ echo ($\pi/2$ = 12 ns, RF pulse = 20 μs, τ = 300 ns).

Echo-detected field sweep spectra at D-band (129.996 GHz) were collected using a home-built 130 GHz EPR spectrometer equipped with an Oxford-CF935 liquid helium cryostat as described previously.[7] Magnetic field-swept echo-detected EPR spectrum was acquired using the Hahn echo pulse sequence: $\pi/2 - \tau - \pi -$ echo, with $\pi/2$ pulse duration = 35 ns and τ = 250 ns at various

magnetic field values. ELDOR-detected NMR (EDNMR) spectra were collected using the pulse sequence $\pi_{HTA} - T - \pi/2 - \tau - \pi - \tau -$ echo. The length of the high turning angle pulse (π_{HTA}) was set to 14 μs, and the length of microwave pulse $\pi/2$ was set to 35 ns. The interpulse delays T and τ were set to 6 μs and 250 ns, respectively. A Lorentzian function was used to model the central blind spot and subtracted from the raw EDNMR spectra as previously described.[8] EDNMR spectra were simulated in the same way as ENDOR spectra.

Simulations of all EPR spectra were generated using the Easyspin 6.0.0 toolbox[9] in the Matlab R2021b software suite (Mathworks Inc., Natick, MA).

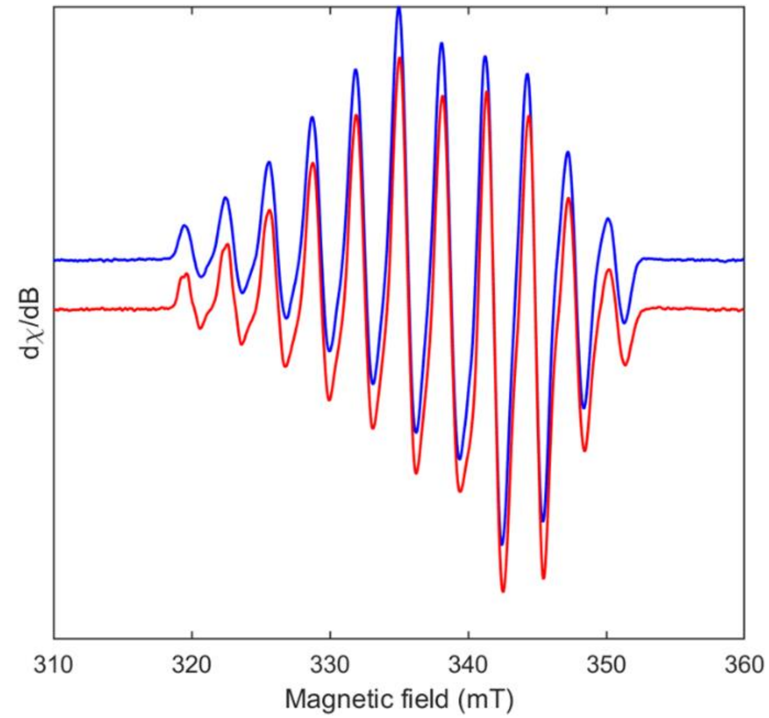

Figure S37. CW X-band spectra of **1-Y** (blue trace) and **1-Y$_D$** (red trace) measured at 30 K. Experimental conditions: microwave frequency, 9.3886 GHz (**1-Y** or 9.3901 GHz (**1-Y$_D$**); microwave power, 20 μW; modulation amplitude, 0.3 mT; modulation frequency, 100 kHz; conversion time, 59 μs.

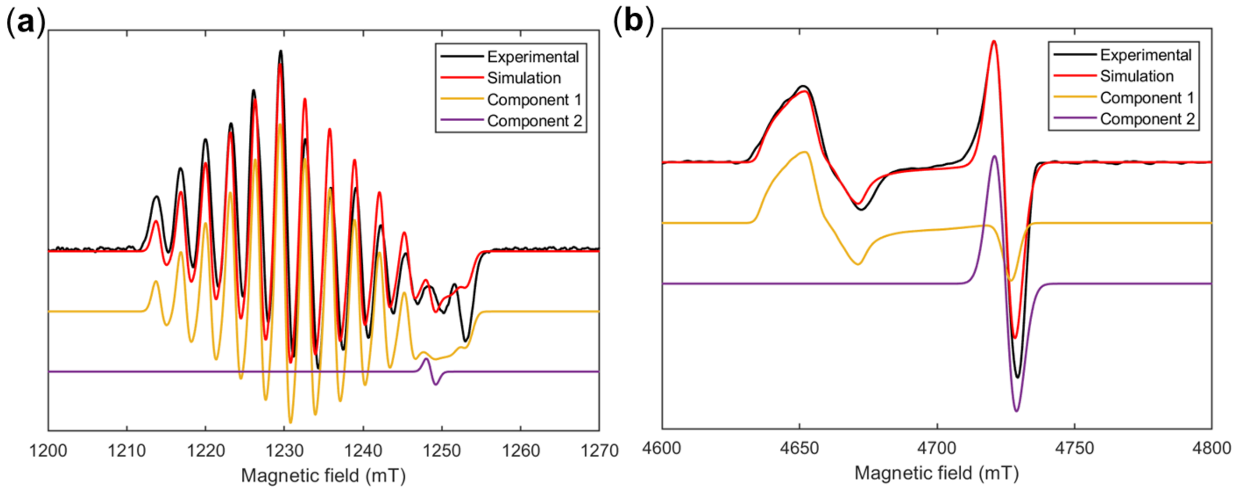

Figure S38. (a) Pseudo-modulated echo-detected field-sweep Q-band spectrum of **1-Y$_\mathbf{D}$** collected at 24 K; microwave frequency: 34.352 GHz, pseudo-modulation amplitude: 1 mT (b) Pseudo-modulated echo-detected field-sweep D-band spectrum of **1-Y** collected at 12 K; microwave frequency: 129.996 GHz, pseudo-modulation amplitude: 10 mT. Black traces represent experimental data. Yellow, purple, and red traces represent the first component, second component (impurity), and total simulation, respectively. Simulation of Q-band data used 2% impurity, while that of D-band data used 15% impurity. The impurity in the D-band spectrum is attributed to the fact that sample was transported to the EPR facility within an open tube inside of a sealed cryovial, which is not air-tight, and the sample was directly exposed to air while being loaded into the EPR spectrometer, as described in Section 7.1 above. However, the D-band frequency is high enough to separate most of the **1-Y** signal (g = [1.9957, 1.9957, 1.965]) from that for the impurity (g = 1.9657), and therefore the impurity is not expected to greatly affect the interpretation of this data. Additionally, the EDNMR spectra collected on the D-band sample (see Figure S41) are impacted by the impurity only at the highest field (4.725 T), and thus the conclusions drawn from these spectra are not meaningfully impacted by the impurity.

7.2 EDNMR Spectroscopy

Electron–electron double resonance (ELDOR) spectroscopy is a pulse EPR technique that utilizes two microwave pulses. ELDOR-detected nuclear magnetic resonance (EDNMR) spectroscopy is a technique to study hyperfine interactions of a paramagnetic system. It involves application of a long microwave pulse—termed a high-turning-angle (HTA) pulse—to excite the system from the ground state to (forbidden) excited states, effectively depopulating the ground state. The second pulse is the EPR probe pulse, which typically is the Hanh echo sequence ($\pi/2 - \tau - \pi - \tau$ – echo) that measures the EPR transition of the system, after it has been altered by the HTA pulse.

Figure 43a below depicts a standard Hanh echo sequence used to measure EPR signal, which appears as an echo signal. Figure 43b depicts a typical EDNMR sequence, where a HTA pulse is applied before the Hanh echo sequence. The HTA pulse alters the ground state population of the system and therefore the echo intensity of the EPR signal. By varying the frequency of the HTA pulse (v), the hyperfine energy levels can be probed.

Consider a simplified example of **1-Y** with an unpaired electron ($S = {}^1/_2$) coupled to a ^{127}I nucleus ($I = {}^5/_2$). The energy levels of the system can be described by a Hamiltonian:

$$\hat{H} = \mu_B B \cdot g \cdot \hat{S} + \hat{S} \cdot A \cdot \hat{I} - \mu_N B \cdot g_N \cdot \hat{I}$$

where the first, second, and third terms are electronic Zeeman, electron–nuclear hyperfine, and nuclear Zeeman interactions, respectively (the nuclear quadrupole interaction is ignored for the sake of simplicity).[10] Here, S and I are electron and nuclear spin operators, A is the hyperfine tensor, g is electronic g tensor, g_N is nuclear g factor, μ_B is Bohr magneton, μ_N is nuclear magneton, and B is magnetic field. Figure 43c displays an energy level diagram of the system upon introduction of the three interactions in respective order. The electronic Zeeman energy is generally much larger than hyperfine and nuclear Zeeman interactions. On the other hand, hyperfine and nuclear Zeeman interactions can be of the same order of magnitude, depending on magnetic field and hyperfine interaction strength. Figure 43c depicts the case of strong coupling (the hyperfine interaction is larger than the nuclear Zeeman interaction).

The black double-headed arrow represents a standard EPR transition (selection rule $\Delta M_S = \pm 1$, $\Delta M_I = 0$). Red arrows represent forbidden transitions facilitated by the HTA pulse ($\Delta M_S = \pm 1$, $\Delta M_I = \pm 1, \pm 2, \ldots$). Note that only a few selected transitions are shown for the sake of simplicity. For full details on EDNMR, see ref. 11). By sweeping the frequency of the HTA pulse (ν), the energy levels of the system may be mapped. To obtain information on nuclear magnetic resonance (NMR) transition ($\Delta M_I = \pm 1$, $\Delta M_S = 0$, labeled as blue double-headed arrow in Figure 43c), one subtracts the frequency of EPR transition (ν_0) from HTA pulse frequency (ν). The EDNMR spectrum reports signal intensity as a function of $|\nu - \nu_0|$. The signals appear as negative peaks, as they correspond to the loss of EPR signal, but are subsequently converted to positive peaks by inverting the data. Figure S44 (left) depicts the resulting EDNMR spectra of **1-Y** measured at six different field positions along the EPR signal envelope shown on the right. The signal at $\nu - \nu_0 = 0$ corresponds to a normal EPR transition and is subtracted out through background subtraction (using a Lorentzian function centered at $\nu - \nu_0 = 0$), generating a blind spot where any data near this position may be concealed. Multiple quantum transitions were also observed and simulated (Figure S44, orange, yellow, green, and purple lines). These transitions originate from the $\Delta M_I = \pm 2, \pm 3, \pm 4, \pm 5$ transitions (Figure 43c, orange, yellow, green, and purple arrows). Simulations of EDNMR spectra were performed in a similar fashion to that described by Cox et al.[12] The ^{127}I nucleus was simulated using the salt function of the Easyspin 6.0.0 toolbox.[9] Multiple-quantum transitions were simulated by scaling the frequency relative to the single quantum transitions. The linewidth and intensity of the transitions were manually adjusted to fit the spectra.

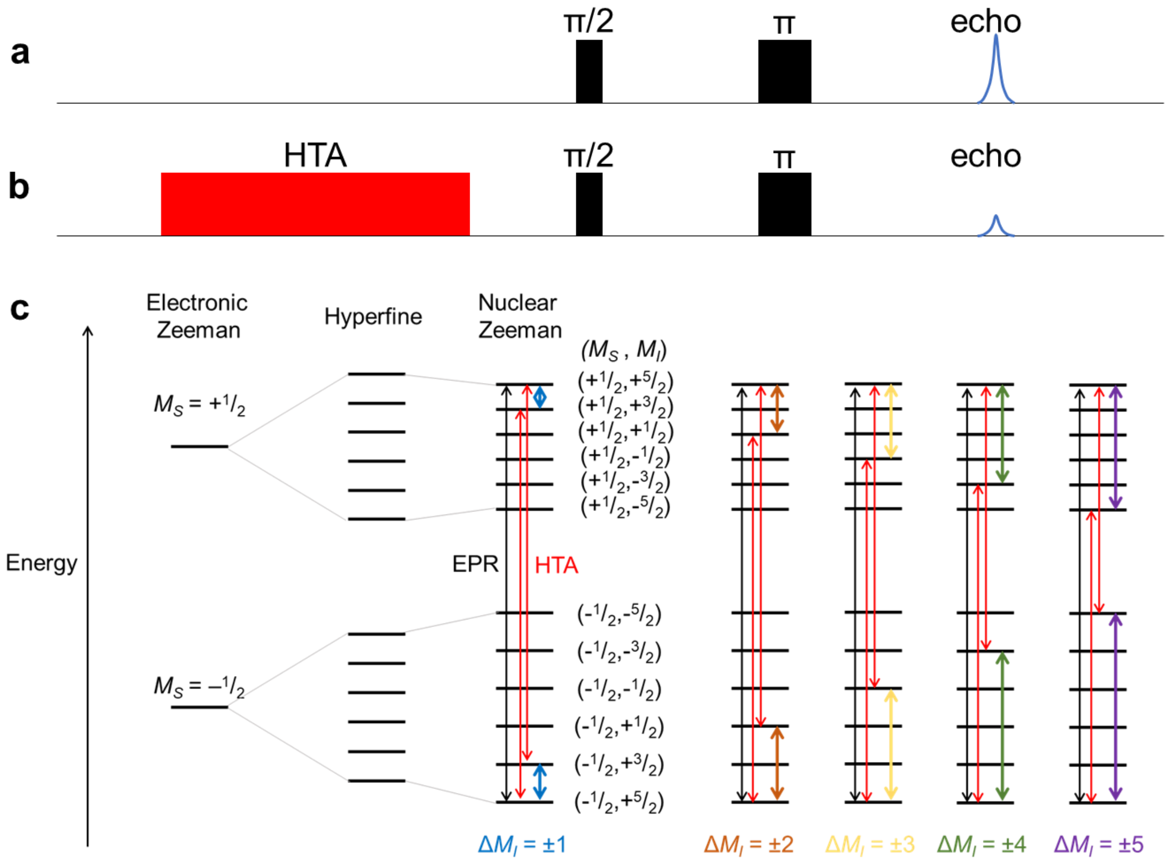

Figure 39. Hahn echo pulse sequence (a) and EDNMR pulse sequence (b). Black rectangles indicate microwave pulses at the EPR frequency (ν_0). Red rectangle represents the HTA pulse, which was applied at a variable frequency ν. (c) Energy level diagram of an $S = {}^1/_2$ spin system subjected to electronic Zeeman, electron–nuclear hyperfine, and nuclear Zeeman interactions, respectively. The EPR, EDNMR, and NMR transitions are labeled with black, red, and blue double-headed arrows, respectively. Each state is labeled by the two quantum numbers (M_S, M_I) to the right. Four additional energy diagrams illustrate the multiple-quantum NMR transitions $\Delta M_I = \pm 2, \pm 3, \pm 4, \pm 5$ (orange, yellow, green, and purple arrows, respectively).

7.3 Analysis of Hyperfine Coupling Parameters

Hyperfine interactions may be decomposed into isotropic (A_{iso}) and anisotropic (T) parts.

$$\boldsymbol{A} = A_{\mathrm{iso}} \begin{pmatrix} 1 & 0 & 0 \\ 0 & 1 & 0 \\ 0 & 0 & 1 \end{pmatrix} + \boldsymbol{T}$$

For an axial system,

$$\boldsymbol{A} = \begin{pmatrix} A_{\perp} & 0 & 0 \\ 0 & A_{\perp} & 0 \\ 0 & 0 & A_{||} \end{pmatrix} = A_{\mathrm{iso}} \begin{pmatrix} 1 & 0 & 0 \\ 0 & 1 & 0 \\ 0 & 0 & 1 \end{pmatrix} + T \begin{pmatrix} -1 & 0 & 0 \\ 0 & -1 & 0 \\ 0 & 0 & 2 \end{pmatrix}$$

Thus, it follows that $A_{\mathrm{iso}} = \frac{2A_{\perp}+A_{||}}{3}$ and $T = \frac{A_{||}-A_{\perp}}{3}$. The component T is composed of local contribution (T_{local}), which originates from spin density on p, d, or f orbitals, and non-local contribution ($T_{non\text{-}local}$), which originates from through-space dipolar interactions:

$$T = T_{\mathrm{local}} + T_{\mathrm{non-local}}$$

Assuming that electron density is equally divided among the three Y ions, we approximate $T_{non\text{-}local}$ contributions of ~0.4 and ~0.04 MHz for dipolar interaction between a point charge on three Y nuclei, each with $^1/_3$ of an electron charge, and the nuclear spin of ^{127}I and ^{89}Y, respectively, using the equation:

$$T = \sum_i \frac{1}{4\pi} \mu_0 g \mu_B g_N \mu_N \left(\frac{3\, \vec{r_i} \cdot \vec{r_i}}{{r_i}^5} - \frac{1}{{r_i}^3} \right)$$

where μ_0 is the magnetic permeability of free space and $\vec{r_i}$ is a vector connecting the point charge on each Y nucleus with the nucleus of interest.[13] These values are negligible compared to experimental T (^{127}I) and T (^{89}Y) and thus are omitted in the subsequent calculations. An estimate $T_{non\text{-}local}$ (^2H) of ~0.4 MHz was obtained using the same calculation, which agrees with the experimental T (^2H) of 0.27 MHz, because no p orbital is involved in the bonding of H/D ligands, thus the absence of local contribution to T (^2H).

Morton and Preston calculated isotropic hyperfine parameters for unit spin density (A) on an s orbital, and anisotropic hyperfine parameters (P) of unit spin density on a p, d, or f orbital for various elements:[14]

$$A = \frac{8\pi}{3} g \mu_B \gamma |\Psi(0)|^2$$

$$P = g \mu_B \gamma \, \langle r^{-3} \rangle$$

where γ is the magnetogyric ratio of a given nucleus. By comparing our experimental A_{iso} and T_{local} values with those from Morton and Preston, it was possible to extract the electron density on each orbital: %s = $A_{iso}/A \times 100$ and %p (or %d) = $T_{local}/\alpha P \times 100$, where α is the angular factor of the orbital (α = $^2/_5$ for p orbitals, and $^2/_7$ for d orbitals). The resulting electron densities are listed in Table 1 in the manuscript.

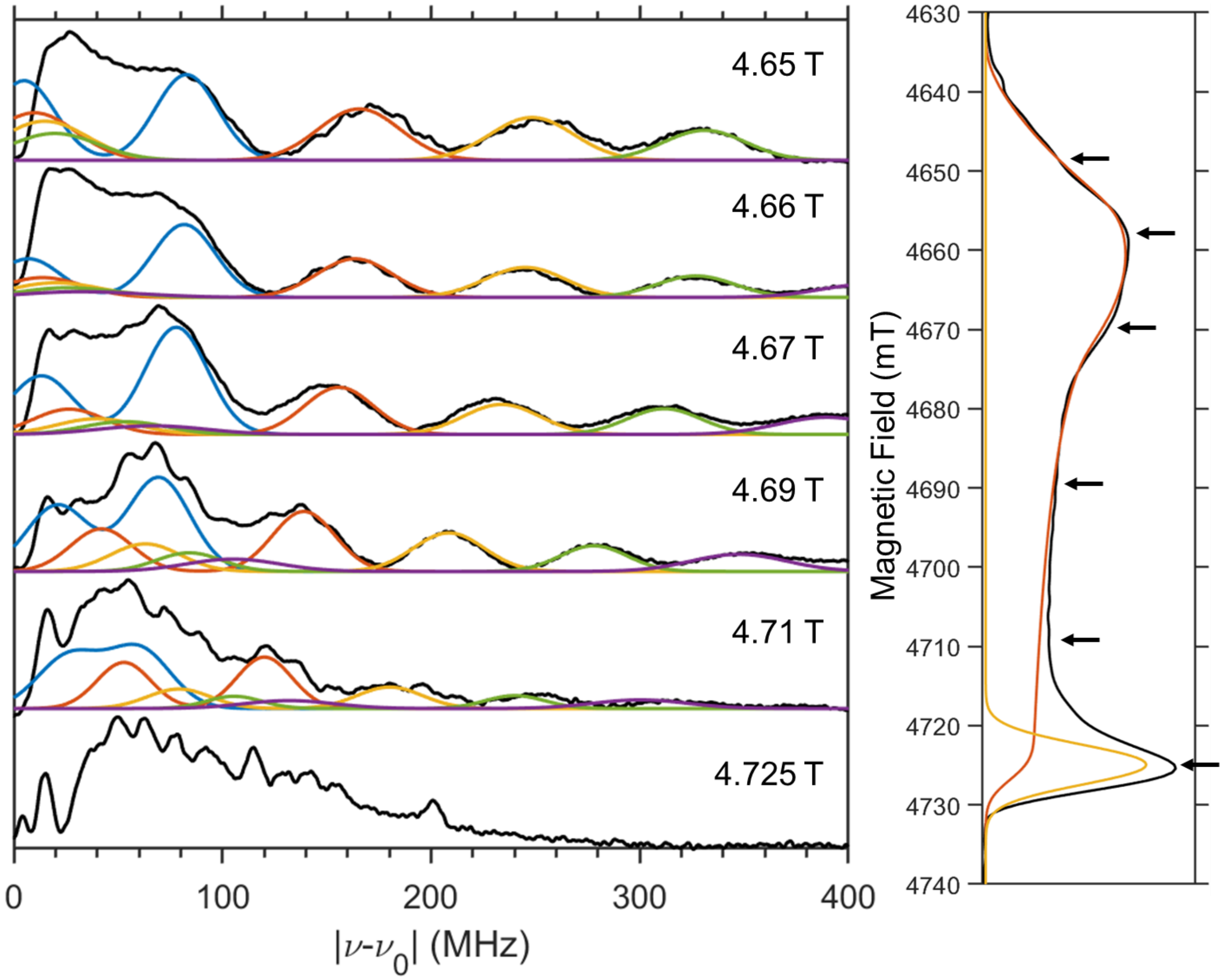

Figure S40. (Left) D-band EDNMR spectra of **1-Y** (black traces) collected at 12 K at the indicated magnetic fields. Simulations of the spectra were generated using an anisotropic ^{127}I hyperfine parameter (blue traces) of A = [87, 87, 20] MHz, AFrame = [0, 0, 0]°, and an axial g tensor of [1.9957, 1.9957, 1.965]. The AFrame denotes the Euler angles, which define the orientation of the A tensor with respect to the g tensor in the convention used in EasySpin software.[9] Multiple quantum transitions were observed as a result of the large quadrupole moment of the ^{127}I nuclei and are plotted as red, yellow, green, and purple traces for ΔM_I = ±2, ±3, ±4, and ±5 transitions, respectively. (Right) Echo-detected field-sweep D-band spectrum of **1-Y** (black trace). The spectrum was fit to two paramagnetic components as indicated with red and yellow traces, the latter of which was assigned to an impurity constituting ~15% of the overall signal resulting possibly from air exposure of the sample. Arrows indicate the magnetic fields at which the EDNMR spectra (left) were measured.

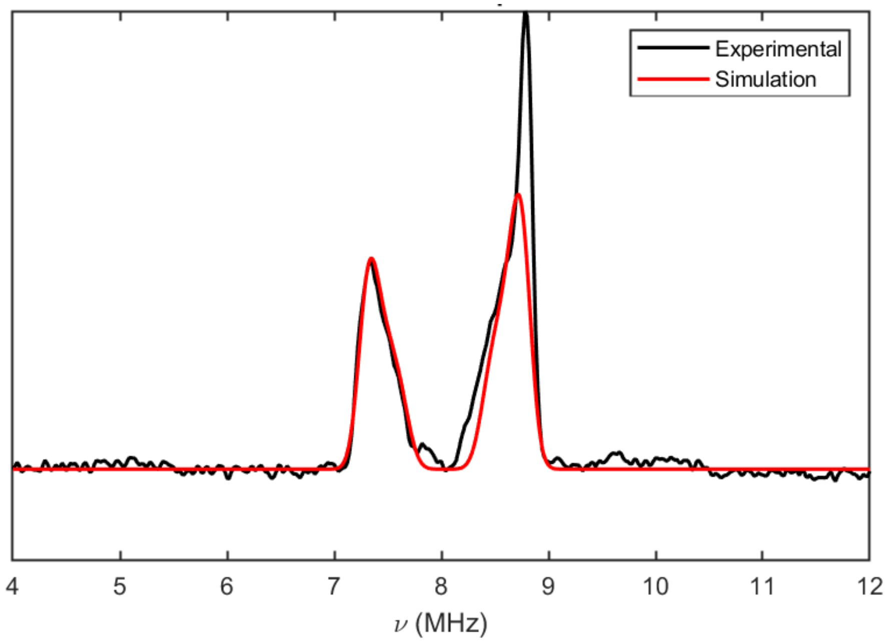

Figure S41. Q-band ^{2}H Mims ENDOR spectrum of **1-Y$_D$** (black line) and a corresponding simulation (red line). Experimental conditions: magnetic field, 1.229 T; temperature, 20 K; microwave frequency, 34.352 GHz. Simulation parameters: $A(^2\text{H}) = [1.5, 1.5, 0.7]$ MHz.

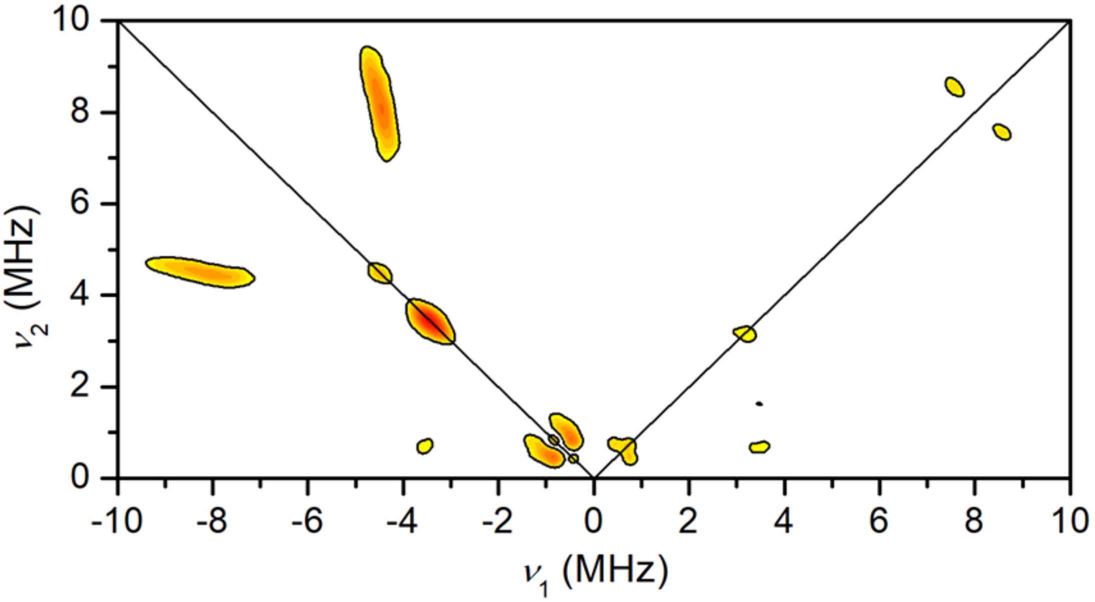

Figure S42. Q-band HYSCORE spectrum of **1-Y$_D$**, presented as a red-yellow contour map. Black lines enclosing the data mark the contour boundary of depth 1.5 (arbitrary unit). Experimental conditions: magnetic field, 1.229 T; temperature, 20 K; microwave frequency, 34.351 GHz; π/2 pulse, 16 ns; τ, 300 ns. Figure 2b in the main text shows this spectrum overlaid with simulations of ^{89}Y and ^{2}H hyperfine interactions. Signals below $|\nu_1|$ or $|\nu_2|$ < 4 MHz are likely artefacts or unidentified hyperfine interactions.

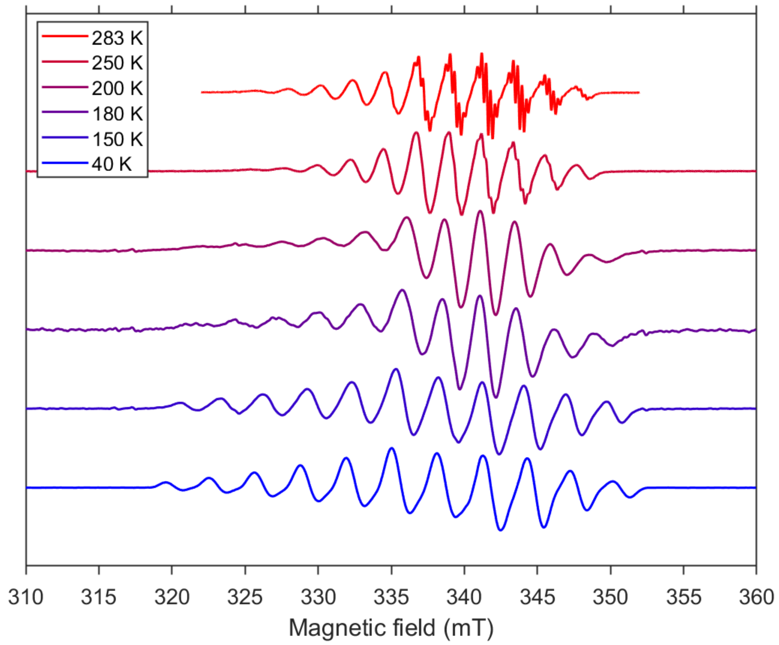

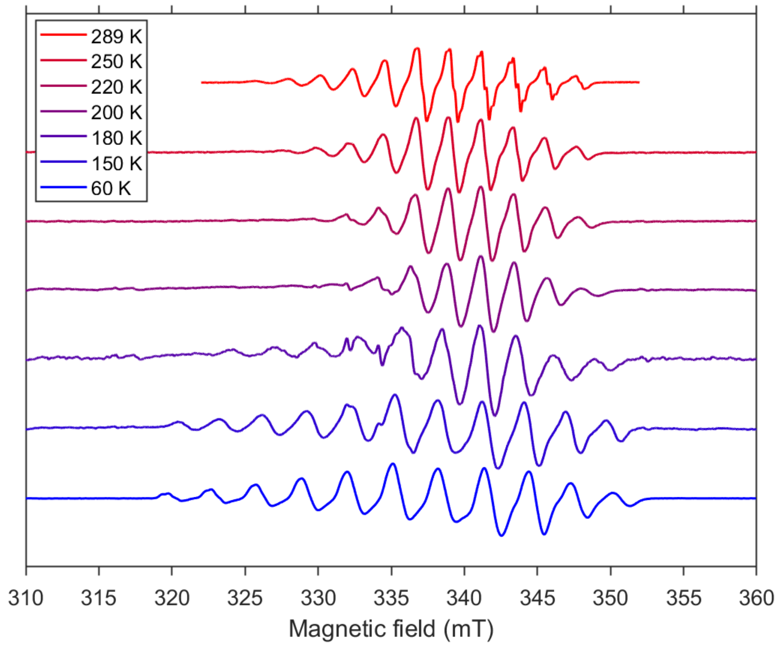

Figure S43. (Upper) Variable-temperature CW X-band spectra of **1-Y**. Experimental conditions: microwave frequency, 9.388–9.389 GHz; microwave power, 0.2 mW (280–150 K) or 20 μW (40 K); modulation frequency, 100 kHz; modulation amplitude, 0.1 or 0.3 mT. Spectra were normalized to the same maximum intensity. (Lower) Variable-temperature CW X-band spectra of **1-Y$_D$**. Experimental conditions: microwave frequency, 9.388–9.390 GHz; microwave power, 0.2 mW (1 mW for 200 K and 20 μW for 60 K); modulation frequency, 100 kHz; modulation amplitude, 0.3 mT (0.1 mT for 289 K). Spectra were normalized to the same maximum intensity.

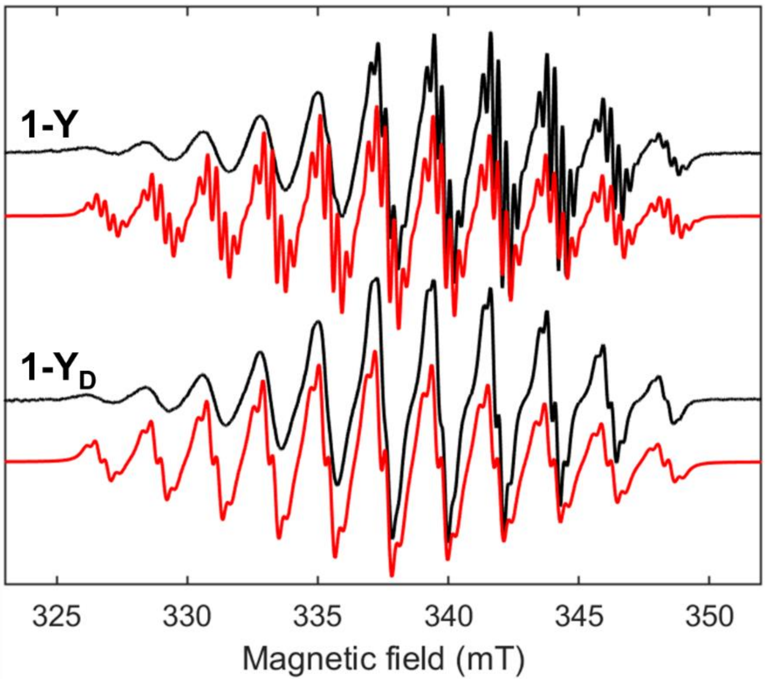

Figure S44. CW X-band spectra of **1-Y** (top) and **1-Y$_D$** (bottom) measured at 283 and 289 K, respectively. Black lines are experimental data and red lines are simulations using g_{iso} = 1.986, two A_{iso} (^{127}I) = 60 MHz, three A_{iso} (^{89}Y) = 8.8 MHz, and (for **1-Y** only) three A_{iso} (^{1}H) = 7.82 MHz. A_{iso} (^{1}H) = 7.82 MHz for **1-Y** corresponds to A_{iso} (^{2}H) = 1.20 MHz for **1-Y$_D$**. This hyperfine splitting is too small to be resolved by CW EPR spectroscopy and thus A_{iso} (^{2}H) was not included in the simulation. Experimental conditions: microwave frequency, 9.39 GHz; microwave power, 0.2 mW; modulation frequency, 100 kHz; modulation amplitude, 0.1 mT; conversion time, 29 ms.

8. Computational Methods and Analysis

For calculation of the IR spectra of **1-Y** and **1-Y$_D$**, geometry optimization and calculation of the normal modes of vibration were performed in the gas-phase using unrestricted DFT within the Gaussian 09 rev. D package.[15] The single-crystal structure of **1-Y** was used as a starting point, and all atomic positions were optimised simultaneously. The PBE density-functional was used in conjunction with Grimme's D3 dispersion correction,[16-20] the cc-pVTZ basis set was used for C, cc-pVDZ for H atoms, cc-pVTZ-PP for I atoms,[21,22] while the Stuttgart RSC 1997 effective core potential (ECP) was employed for the 28 core electrons of Y and the remaining valence electrons were described with the corresponding valence basis set.[23,24] Upon optimisation, the structure of **1-Y** is modified only slightly, with an overall root-mean-squared difference of 0.198 Å across the entire structure (Table S8). The vibrational modes of the deuterated isotopologue **1-Y$_D$** were calculated by substituting the three hydride ions for deuterides (using the masses defined within the Gaussian code) and repeating the frequency analysis portion of the calculation in Gaussian using the same Hessian matrix as for **1-Y** (Table S9). The theoretical IR spectra were generated by summing over the contributions of all modes $\{j\}$, where each mode is represented by a Gaussian function centred at the DFT calculated mode frequency ν_j, with an area equal to the calculated linear absorption coefficient, and a full-width-at-half-maximum of 15 cm^{-1}.

The calculated IR spectra of **1-Y** and **1-Y$_D$** are identical save for a handful of signals in the region 300–1500 cm^{-1} (Figure S48) and agree well with the experimental spectra, which also only differ within this region (Figure S11). The Vibrational Projection Analysis (ViPA) technique allows us to quantify the similarity between the normal modes of **1-Y** and **1-Y$_D$** (Table S10).[25] The calculated intense signal at 621 cm^{-1} matches well with the experimental signal at 637 cm^{-1}, and corresponds to a hydride mode in which all hydrides move out of the Y_3 plane in phase (mode 156). ViPA shows us that the calculated mode at 442 cm^{-1} (mode 132) in **1-Y$_D$** is a 98% match to mode 156 of **1-Y**, again in good agreement with the experimental signal at 457 cm^{-1}. Similarly, the two signals calculated at 982 and 987 cm^{-1} for **1-Y**—which are hydride symmetric and antisymmetric stretching modes within the Y_3 plane (modes 218 and 219)—match well with the experimental absorbances at 972 cm^{-1}. These signals shift lower in energy in **1-Y$_D$** to 697 and 700 cm^{-1} (modes 158 and 159, corresponding to experimental signal at 696 cm^{-1}), but now also include out of plane Cp vibrational character due to the Cp modes in both **1-Y** and **1-Y$_D$** at ~700 cm^{-1}. Finally, the intense signals at 1270–1370 cm^{-1} (experimental signals at 1240–1390 cm^{-1}) in **1-Y** are caused by a group of vibrational modes (modes 292-302) which all involve considerable motion of both the hydride and Cp ligands. These modes consist of hydride symmetric and antisymmetric stretches within the Y_3 plane which occur along with Cp *tert*-butyl C–H bends and stretches. However, in **1-Y$_D$** these are effectively split into two sets. Set i) consists of a pair of pure deuteride stretching modes at 922 and 927 cm^{-1} (modes 206 and 207) which can be described by a linear combination of modes 292-302 of **1-Y** such that the C–H components are eliminated (experimental signals at 910 cm^{-1}). Set ii) remains at 1350 cm^{-1}, though are much less intense, and correspond to pure *tert*-butyl C–H stretches of **1-Y$_D$**.

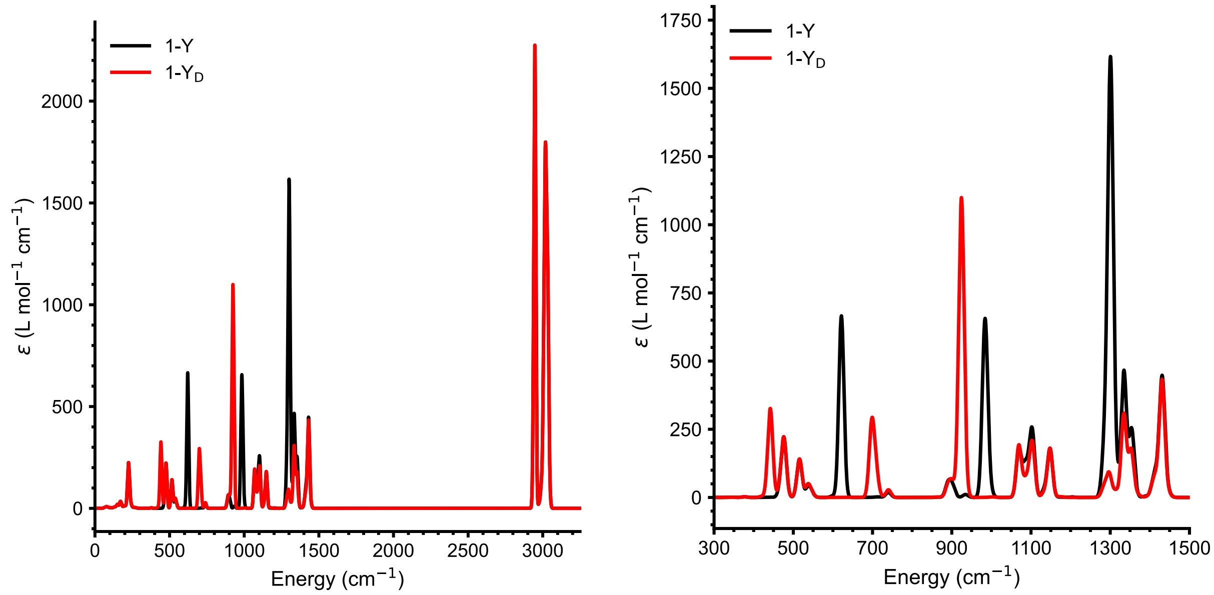

Figure S45. Left: Calculated IR spectra for **1-Y** and **1-Y$_D$**. Right: zoom in on the 300-1500 cm^{-1} region.

Table S8. Optimized coordinates for **1-Y**.

Atom	x (Å)	y (Å)	z (Å)
I	-0.020336	0.019683	-2.413380
I	0.021866	-0.043140	2.441698
Y	-1.734346	-1.078317	0.002599
Y	1.794357	-0.973081	0.003106
Y	-0.061117	2.026507	0.024769
C	-4.317103	-1.189986	-0.492695
C	-4.180782	-1.458338	0.913422
C	-3.543777	-2.737486	1.058960
C	-3.361516	-3.541293	2.340818
H	-2.897006	-4.488669	2.032453
C	-5.063162	0.001006	-1.081734
H	-5.251089	0.686799	-0.241045
C	-2.838555	-4.672139	-0.604007
H	-2.781913	-4.703697	-1.701247
C	-3.287975	-3.259713	-0.251934
C	-4.213431	0.751326	2.287728
H	-3.114552	0.755747	2.371446
H	-4.623086	1.182283	3.217209
H	-4.482949	1.426580	1.463038

C	-3.766112	-2.305538	-1.213119
C	-1.442389	-5.072705	-0.109467
H	-0.691015	-4.330746	-0.417235
H	-1.155892	-6.054118	-0.524517
H	-1.391511	-5.151696	0.987763
C	-3.890277	-5.726401	-0.206998
H	-3.937317	-5.870970	0.883231
H	-3.641039	-6.701346	-0.658646
H	-4.895062	-5.434982	-0.549432
C	-1.207031	4.453477	0.197343
C	-3.928700	-2.531002	-2.712583
H	-4.338927	-1.594631	-3.115892
C	-4.759802	-0.669121	2.082539
H	-4.469023	-1.226649	2.984917
C	-2.400172	-2.937371	3.374034
H	-1.390614	-2.818006	2.949238
H	-2.316361	-3.599053	4.252796
H	-2.725108	-1.947211	3.730587
C	3.237517	-3.073320	-0.627486
C	3.359669	-2.955075	0.799915
C	1.085472	4.328366	0.558345
C	3.928762	-1.962134	-1.225119
C	5.083530	1.317779	0.294610
H	4.048765	1.596869	0.048458
H	5.756174	2.120027	-0.053554
H	5.160852	1.280361	1.392370
C	-4.708392	-3.929089	2.980155
H	-5.212285	-3.069039	3.446686
H	-4.551503	-4.685955	3.766892
H	-5.393521	-4.350933	2.228315
C	1.446323	-4.221495	2.009615
H	0.892090	-3.283126	2.175492
H	1.253217	-4.885993	2.869058
H	1.018578	-4.697268	1.116496
C	-0.192981	4.397240	1.213489
C	0.858358	4.342938	-0.861144
C	4.472269	-1.158808	-0.164876
C	-6.298952	-0.663924	2.062003
H	-6.692839	-0.024983	1.256649
H	-6.693366	-0.275289	3.015915
H	-6.697041	-1.679638	1.911862
C	-4.288967	0.827754	-2.122708
H	-3.315287	1.150831	-1.722531
H	-4.860939	1.733023	-2.386182
H	-4.103588	0.274370	-3.056000

C	2.747435	3.286347	2.255670
H	2.125115	3.378972	3.158970
H	3.801620	3.337641	2.575577
H	2.576386	2.285187	1.829749
C	4.120819	-1.771772	1.083012
C	-2.638075	-2.788792	-3.505395
H	-1.930025	-1.954147	-3.392540
H	-2.866900	-2.892973	-4.579360
H	-2.117330	-3.706638	-3.190130
C	-3.682827	3.756293	-0.119290
H	-3.740963	3.811424	-1.217276
H	-4.694108	3.957623	0.273188
H	-3.404437	2.724656	0.141311
C	6.897382	-0.426306	-0.007074
H	7.611358	0.340475	-0.351986
H	7.169023	-1.385141	-0.474308
H	7.030071	-0.535221	1.080355
C	-0.558696	4.420194	-1.081184
C	1.505380	-3.928262	-2.362697
H	0.718363	-3.307142	-1.905776
H	1.031240	-4.856371	-2.724232
H	1.885736	-3.388885	-3.244021
C	3.726058	-5.287710	1.733523
H	3.392727	-5.870242	0.860879
H	3.570014	-5.913611	2.628195
H	4.806585	-5.105575	1.624071
C	-6.443952	-0.406856	-1.629620
H	-6.353478	-1.011942	-2.545192
H	-7.037627	0.488275	-1.880389
H	-7.006726	-0.998881	-0.891715
C	2.860191	3.285546	-2.143516
H	3.546042	3.187626	-1.289947
H	3.474776	3.427343	-3.048878
H	2.324600	2.328146	-2.246266
C	1.887542	4.461958	-1.979426
H	1.303936	4.489208	-2.911187
C	-4.986259	-3.611231	-3.012998
H	-4.621366	-4.622439	-2.777004
H	-5.250840	-3.597352	-4.083644
H	-5.903791	-3.440038	-2.428744
C	-0.904913	6.044367	-3.007308
H	-0.956981	6.839786	-2.247514
H	-1.602571	6.299996	-3.822368
H	0.111138	6.051500	-3.430035
C	3.676569	-5.174924	-1.978677

H	4.153802	-4.697251	-2.848497
H	3.219827	-6.116835	-2.326252
H	4.469052	-5.421732	-1.255464
C	-2.675592	4.754142	0.468675
H	-2.798818	4.689070	1.559069
C	2.651343	5.796905	-1.914845
H	1.959128	6.644968	-1.795677
H	3.231198	5.951298	-2.840315
H	3.360533	5.819908	-1.073018
C	2.458484	4.383585	1.216783
H	3.187690	4.222373	0.407715
C	5.697137	-2.367673	2.988294
H	5.204024	-3.279053	3.358886
H	6.255954	-1.929240	3.832034
H	6.421511	-2.669721	2.215794
C	2.754520	5.777942	1.800273
H	2.551861	6.569382	1.062421
H	3.812284	5.850885	2.104259
H	2.138932	5.984120	2.689842
C	-0.823760	6.075664	3.009000
H	-0.192697	6.799560	2.470228
H	-0.740748	6.281779	4.089338
H	-1.869338	6.258712	2.718001
C	-3.045741	6.200792	0.086801
H	-2.321419	6.920177	0.498333
H	-4.045048	6.454379	0.478940
H	-3.074330	6.342348	-1.004607
C	3.824850	-0.516538	-3.386168
H	2.751302	-0.316214	-3.251555
H	4.022836	-0.567047	-4.470070
H	4.374711	0.352682	-2.993153
C	4.692420	-1.341912	2.428931
H	5.281818	-0.437354	2.224814
C	-1.189014	3.555174	-3.442838
H	-0.152859	3.335586	-3.745353
H	-1.751617	3.830767	-4.350933
H	-1.621427	2.620075	-3.052602
C	-1.288884	3.620766	3.438167
H	-2.329415	3.637137	3.077803
H	-1.311481	3.844327	4.518049
H	-0.914475	2.592836	3.317301
C	2.609721	-4.262700	-1.344989
H	2.116185	-4.858802	-0.561960
C	5.457083	-0.014771	-0.370354
H	5.463427	0.186754	-1.450965

C	2.951796	-3.963998	1.866665
H	3.266096	-3.522803	2.823670
C	5.705966	-2.171865	-3.020495
H	6.385184	-1.374048	-2.683248
H	5.851874	-2.292908	-4.107049
H	6.011847	-3.106254	-2.524762
C	-1.277879	4.678146	-2.400227
H	-2.344221	4.752068	-2.145174
C	3.662961	-0.923807	3.487916
H	3.063691	-0.068233	3.137516
H	4.171681	-0.618485	4.418000
H	2.960480	-1.733567	3.741467
C	-0.387450	4.628808	2.708144
H	0.607151	4.517270	3.162725
C	4.233560	-1.836592	-2.714056
H	3.638284	-2.616562	-3.210281
H	1.866669	1.141955	0.001852
H	-1.931076	1.033077	0.043292
H	0.063582	-2.202734	-0.012960

Table S9. Vibrational mode energies and absorption intensities for **1-Y** and **1-Y$_D$**.

Mode	**1-Y** Energy (cm^{-1})	**1-Y** Intensity (km mol^{-1})	**1-Y$_D$** Energy (cm^{-1})	**1-Y$_D$** Intensity (km mol^{-1})
1	17.8049	0.0010	17.8041	0.0010
2	20.1197	0.0020	20.1186	0.0020
3	24.0583	0.0094	24.0460	0.0093
4	30.7192	0.0043	30.7137	0.0043
5	31.6089	0.0028	31.6029	0.0028
6	38.3733	0.0399	38.3621	0.0397
7	43.5791	0.0100	43.5344	0.0099
8	44.4420	0.0071	44.3940	0.0071
9	56.4450	0.0265	56.3585	0.0262
10	59.4576	0.1675	59.4448	0.1669
11	61.7608	0.1864	61.7479	0.1875
12	62.6432	0.2175	62.6159	0.2125
13	63.9432	0.1925	63.9195	0.1951
14	65.7318	0.2048	65.7185	0.2081
15	68.6545	0.2033	68.6485	0.2042
16	69.3683	0.2244	69.3615	0.2231
17	73.0474	0.6878	73.0151	0.6846
18	74.7665	0.8052	74.7392	0.7918
19	75.7410	0.2278	75.6877	0.2435
20	78.8206	0.4159	78.7705	0.4114

21	80.9424	0.5072	80.9218	0.5124
22	82.3067	0.5937	82.2813	0.5942
23	84.5777	0.4443	84.5570	0.4463
24	86.4694	0.0823	86.3345	0.0814
25	88.5325	0.0367	88.5247	0.0360
26	92.0085	0.1922	91.9120	0.1933
27	94.5831	0.1597	94.5672	0.1591
28	96.3543	0.0343	96.2863	0.0319
29	98.8028	0.0947	98.7888	0.0958
30	100.6968	0.2774	100.6413	0.2796
31	102.3052	0.6162	102.2698	0.6051
32	104.1342	0.5128	104.1020	0.5101
33	105.8096	0.0790	105.7833	0.0835
34	112.1340	0.1720	112.1026	0.1704
35	114.6574	0.1924	114.6336	0.1909
36	116.3435	0.1636	116.3306	0.1631
37	120.8581	0.0684	120.8451	0.0692
38	122.6765	0.0677	122.6244	0.0671
39	125.1788	0.3358	125.1570	0.3381
40	126.9253	0.2639	126.9104	0.2574
41	128.4921	0.0415	128.4220	0.0373
42	129.9803	0.5534	129.6495	0.6059
43	131.4229	0.3834	131.1744	0.5417
44	131.7378	0.8082	131.4326	0.5397
45	142.2490	0.5029	142.1764	0.4861
46	143.4111	0.3355	143.3380	0.3152
47	145.5166	1.3920	145.4654	1.3326
48	151.2962	5.7951	151.2710	5.7602
49	166.4953	0.0363	166.4747	0.0365
50	167.8691	0.3334	167.8285	0.3295
51	169.1136	0.9807	169.0704	0.9017
52	169.9726	7.0915	169.9449	7.0621
53	171.1639	0.6418	171.1332	0.6172
54	171.7883	2.1519	171.7015	2.0671
55	173.5014	0.5099	173.4712	0.5034
56	174.0026	0.7223	173.9642	0.6581
57	177.5812	0.3944	177.5634	0.3809
58	178.0023	0.2930	178.0005	0.2959
59	180.9866	0.5118	180.9846	0.5037
60	183.7099	0.1639	183.7038	0.1679
61	185.0425	0.3095	185.0145	0.2902
62	187.0125	0.1266	186.9885	0.1254
63	187.7571	0.3858	187.7157	0.3634
64	190.8282	0.0234	190.8152	0.0232
65	192.5898	0.5351	192.5496	0.5310

66	194.1359	0.9254	194.1168	0.9303
67	197.9189	1.2555	197.9070	1.2680
68	198.9424	0.5165	198.9317	0.5284
69	200.2858	0.8357	200.2699	0.8465
70	204.6077	0.3433	204.6033	0.3563
71	208.1825	0.1652	208.1757	0.1714
72	209.2658	0.2663	209.2570	0.2750
73	210.6660	0.5823	210.6608	0.5963
74	211.8907	0.1817	211.8882	0.1875
75	214.0664	0.6100	214.0625	0.6296
76	215.1260	0.5421	215.1163	0.5568
77	216.1505	0.1289	216.1343	0.1404
78	217.9605	0.3409	217.9552	0.3442
79	219.8491	0.0463	219.8338	0.0420
80	222.2084	4.8735	222.1816	5.4132
81	223.2847	8.9392	223.2146	16.7188
82	223.4745	28.7270	223.3796	21.4691
83	225.1729	11.3351	225.1136	14.2497
84	225.5390	21.4719	225.4777	17.7525
85	228.1981	3.6402	228.1791	3.4969
86	229.6731	1.8556	229.6588	1.8142
87	229.9936	3.1766	229.9835	3.0450
88	231.3842	0.0406	231.3740	0.0411
89	232.2125	0.5469	232.2048	0.5673
90	235.1093	0.0931	235.1039	0.0909
91	235.5158	1.7622	235.4975	1.6855
92	235.7869	0.3856	235.7821	0.3857
93	237.7612	2.5611	237.7428	2.5160
94	241.4813	1.5425	241.4705	1.5162
95	244.1016	0.7297	244.0961	0.7153
96	244.5751	1.0189	244.5668	1.0118
97	246.1464	0.0681	246.1336	0.0666
98	246.4540	0.0626	246.4285	0.0682
99	247.2153	0.1461	247.2055	0.1456
100	255.5301	0.0780	255.5102	0.0912
101	256.4239	0.1566	256.3452	0.1511
102	257.9411	0.4721	257.8647	0.5740
103	259.0566	0.2130	258.9761	0.2742
104	260.0413	0.0483	259.9829	0.0621
105	262.0230	0.1002	261.9761	0.1084
106	267.2312	0.1502	267.2150	0.1452
107	269.6132	0.5598	269.6020	0.5546
108	270.5242	0.6860	270.5087	0.6827
109	291.8787	0.1117	291.7879	0.1927
110	292.3086	0.0737	292.2047	0.1183

111	293.4829	0.0943	293.3910	0.1352
112	294.8026	0.0652	294.7668	0.0916
113	295.2388	0.0125	295.2123	0.0128
114	296.3275	0.0200	296.2956	0.0305
115	341.4627	0.1024	341.2810	0.0984
116	341.9506	0.0505	341.6854	0.0911
117	342.2701	0.1177	342.0670	0.0906
118	342.5590	0.0490	342.5106	0.0968
119	343.1150	0.0360	343.0564	0.0522
120	343.8037	0.0146	343.7647	0.0204
121	376.6616	0.5147	376.6382	0.5191
122	376.8081	0.4370	376.7932	0.4203
123	378.0732	0.0016	378.0309	0.0058
124	418.5902	0.0490	418.3629	0.1392
125	419.3071	0.0365	418.6270	0.0455
126	419.3882	0.0237	419.1658	0.4196
127	419.7858	0.0059	419.5217	0.0502
128	419.9285	0.0395	419.7730	0.3771
129	420.8517	0.0105	420.7080	0.0524
130	464.4560	0.4617	426.7706	0.4003
131	465.3414	1.3197	427.6736	0.3151
132	465.9612	0.9094	441.8459	119.9898
133	466.3642	0.1219	464.4593	0.6896
134	468.1488	3.4351	465.3566	2.0517
135	469.1075	2.8204	465.9624	1.1621
136	476.0830	38.2821	466.3658	0.1276
137	476.3855	38.7658	468.1585	3.7418
138	477.5798	1.0024	469.1135	2.8932
139	515.4882	25.7943	476.0725	37.9755
140	515.6066	23.0463	476.3753	38.4103
141	516.1355	2.9077	477.5382	1.0171
142	535.4843	0.8325	515.5182	25.8584
143	535.5949	3.3685	515.6383	23.2390
144	536.2006	3.7006	516.1555	2.7214
145	539.9707	3.5948	535.4875	0.7812
146	540.1536	2.4625	535.6443	4.5714
147	540.3638	1.4457	536.2589	6.2342
148	544.3250	0.2922	539.7361	3.2055
149	545.0292	0.2998	539.9467	3.1610
150	545.7176	0.2026	540.3877	0.4027
151	547.4498	0.5707	544.3512	0.1100
152	547.6556	0.8529	545.0275	0.2204
153	547.8885	1.7884	545.7206	0.1263
154	599.2052	1.1949	547.4497	0.6094
155	601.0447	0.8692	547.6343	0.9481

156	621.3487	244.8248	547.8265	1.4052
157	705.4851	0.0429	692.2124	3.4454
158	705.8797	0.0523	697.2404	54.0175
159	706.3220	0.0759	699.5489	44.3248
160	707.7635	0.2490	706.9530	3.4022
161	708.2378	0.1876	707.4439	3.3745
162	708.5506	0.1741	707.8140	3.2854
163	720.5764	0.1710	708.5914	1.0190
164	721.0817	0.1631	709.2075	7.1711
165	721.6799	0.0680	709.7518	14.2355
166	739.6041	1.4739	720.5836	0.2106
167	739.7526	1.2831	721.0815	0.2197
168	739.9582	0.9702	721.6883	0.1549
169	740.1716	0.7053	739.6851	1.2513
170	740.3175	1.0199	739.8765	2.8180
171	740.4979	1.5861	739.9730	1.8813
172	861.9916	0.0219	740.2228	1.3239
173	862.1092	0.0425	740.3247	0.8072
174	862.2468	0.0106	740.5668	2.3106
175	863.2626	0.0360	857.2841	0.4528
176	863.5143	0.0611	861.9521	0.0839
177	864.0356	0.0882	862.0796	0.0314
178	874.6995	0.1016	862.3670	0.0359
179	875.1165	0.0602	863.2885	0.0270
180	875.6179	0.0503	863.5149	0.0169
181	888.8301	2.6488	864.1077	0.0288
182	889.5171	5.6955	874.6923	0.0430
183	890.1206	5.8346	875.1153	0.0447
184	890.2453	0.8557	875.6285	0.0324
185	891.0384	0.2803	888.9974	2.2800
186	891.7718	0.7308	889.5816	4.9656
187	892.4939	0.6785	890.1385	3.7820
188	892.8791	1.5409	890.2623	1.1786
189	893.2751	0.4599	891.0565	0.0969
190	896.4135	0.5497	891.8002	0.9679
191	897.0027	0.1068	892.5565	0.9446
192	897.2926	1.3893	892.9380	1.5613
193	899.3699	1.0084	893.3589	0.3863
194	899.8107	1.1763	896.4917	0.6329
195	900.5710	1.6995	897.0274	0.2023
196	901.0280	1.5798	897.2184	1.1605
197	901.1703	1.9248	899.3796	1.1165
198	901.5108	3.3145	899.8126	1.5938
199	903.9703	1.6640	900.5865	2.3455
200	904.3033	1.0762	901.0743	2.5420

201	905.1211	1.8077	901.1882	2.2895
202	932.1222	0.1859	901.5072	3.3529
203	932.1498	0.3325	903.9305	2.3023
204	932.3161	0.0973	904.2957	1.2668
205	933.4942	0.1227	905.1269	2.0378
206	933.7339	0.1925	922.0438	218.1201
207	933.8213	0.2481	926.6511	211.0755
208	934.1728	0.4963	932.1715	0.4282
209	934.4814	0.2049	932.1739	0.5035
210	934.5787	1.4899	932.3479	0.6823
211	935.6011	0.0722	933.5382	0.1454
212	935.9293	0.0030	933.7509	0.3617
213	935.9926	0.0571	933.8503	0.2336
214	937.0543	0.4289	934.1941	0.3653
215	937.1215	0.0525	934.5049	0.6029
216	937.8075	0.2133	934.6291	1.3411
217	980.0010	28.8028	935.6694	0.5624
218	982.3701	122.7345	936.0106	0.7558
219	987.0069	110.1012	936.0383	0.8876
220	998.2859	2.6101	937.1124	0.5418
221	998.5312	2.4472	937.1435	0.0599
222	998.7689	0.9273	937.8376	0.2187
223	999.6916	0.5407	998.0024	0.0121
224	1000.1953	0.1408	998.3094	0.0427
225	1000.4733	0.0378	998.6245	0.0127
226	1068.0491	3.0039	999.6481	0.3106
227	1068.2649	25.3505	1000.1691	0.3601
228	1068.4305	0.1121	1000.4182	0.0935
229	1069.1347	3.2133	1068.0521	3.1961
230	1069.9109	14.8928	1068.2700	25.8099
231	1070.0849	18.0688	1068.4363	0.0528
232	1083.9732	17.6826	1069.1548	3.6262
233	1084.2233	16.7007	1069.9321	17.0724
234	1085.3511	0.1812	1070.0942	20.8625
235	1087.2258	2.4019	1084.1896	8.8910
236	1087.4050	0.1244	1084.4326	8.6860
237	1087.6955	2.7894	1085.2190	0.1625
238	1089.6081	0.9512	1087.3928	0.5936
239	1089.8045	0.6213	1087.3973	0.4560
240	1090.0206	0.2829	1087.9045	0.9883
241	1094.7461	4.4492	1089.5157	1.0803
242	1095.8073	5.4359	1089.7436	0.4479
243	1095.8906	6.0793	1090.0223	0.2007
244	1097.1827	2.6857	1094.7075	3.4710
245	1097.5584	1.8952	1095.6690	5.6775

246	1097.9356	2.7022	1095.8627	2.0490
247	1099.0866	0.5963	1097.1817	2.0317
248	1099.3361	1.3754	1097.5614	1.5352
249	1099.4100	3.2944	1097.9316	2.1243
250	1100.1563	5.1554	1099.0875	0.4324
251	1100.2563	3.8111	1099.3332	1.1570
252	1101.6629	2.9213	1099.4063	1.9083
253	1103.8405	23.2891	1100.1854	3.7941
254	1104.0729	31.1696	1100.2719	2.6146
255	1104.7903	11.3229	1101.6729	1.9355
256	1132.8665	1.6337	1103.8987	18.4759
257	1133.0477	3.4319	1104.1658	26.7716
258	1133.2512	4.6268	1104.8071	11.8513
259	1146.1738	0.8242	1132.8761	0.7949
260	1146.1801	2.1868	1133.0883	2.5230
261	1146.9306	29.7964	1133.2971	2.9509
262	1148.6035	15.4829	1146.1702	1.0482
263	1149.0340	16.0260	1146.1936	1.8291
264	1149.4456	2.4246	1146.9106	28.7238
265	1173.5532	0.0736	1148.6036	16.1470
266	1173.8207	0.1261	1149.0292	16.7446
267	1174.0768	0.1612	1149.4635	2.3128
268	1174.3233	0.0428	1173.5264	0.1113
269	1174.4232	0.0169	1173.8320	0.5554
270	1174.8959	0.0252	1174.0871	0.4860
271	1203.1079	0.7509	1174.3047	0.1292
272	1281.2326	1.6135	1174.4292	0.1302
273	1281.8109	1.5910	1174.8948	0.0411
274	1282.4609	1.9615	1281.2193	1.7756
275	1283.8494	10.2893	1281.8026	2.0468
276	1284.1610	1.3209	1282.4600	2.6965
277	1284.2406	2.9914	1284.0148	0.1711
278	1284.6596	4.0941	1284.1610	0.2691
279	1285.0682	14.6295	1284.2896	0.3525
280	1285.2793	11.0444	1284.7254	0.7972
281	1286.2963	3.9943	1285.3160	0.8755
282	1286.3520	2.3498	1285.6426	1.0619
283	1286.5822	1.0681	1286.4910	0.4319
284	1288.4106	2.4463	1286.6444	0.5543
285	1289.3290	0.7982	1286.7389	0.4686
286	1289.7754	0.7207	1288.4100	2.5383
287	1295.3913	66.0172	1289.3421	1.4469
288	1296.0620	31.2905	1289.7823	1.9346
289	1296.2638	14.9950	1295.7827	3.5911
290	1296.7125	3.7745	1296.4141	1.6016

291	1296.9390	7.3355	1296.4654	6.4751
292	1297.2183	20.8887	1296.7153	3.0013
293	1297.6629	36.0533	1297.0363	1.9532
294	1298.3446	33.0730	1297.5173	4.0111
295	1299.0022	1.7083	1298.0556	0.6685
296	1299.9439	126.8908	1299.0569	2.3382
297	1301.4234	27.7011	1299.1820	5.2983
298	1302.0122	29.3961	1301.6292	1.0029
299	1302.5237	11.7186	1301.9489	0.9479
300	1303.7812	249.5685	1302.6009	0.3571
301	1315.3739	0.1907	1314.5652	4.2778
302	1315.9010	7.6542	1315.1004	3.9033
303	1316.7953	16.9636	1315.3924	0.3656
304	1329.6094	0.1939	1329.4955	0.1081
305	1329.8273	3.6482	1329.7360	1.8981
306	1330.4865	0.2705	1330.4153	0.2746
307	1331.1347	0.9997	1331.0296	0.3201
308	1331.5588	4.6602	1331.4251	2.5416
309	1332.1562	0.9811	1331.9944	1.1413
310	1332.6553	23.9626	1332.5254	15.7446
311	1332.8856	13.8994	1332.7405	12.6905
312	1333.4916	0.6022	1333.3873	0.9942
313	1333.5471	2.5465	1333.5168	1.9200
314	1333.9908	1.1848	1333.9510	1.5343
315	1334.6166	12.5120	1334.5548	9.4376
316	1334.9790	38.9973	1334.7842	28.4923
317	1335.6533	46.8626	1335.2336	24.8502
318	1335.8335	22.6471	1335.7105	12.6216
319	1348.0603	0.7901	1347.9957	1.6176
320	1348.1493	0.0642	1348.0620	0.4070
321	1348.8081	4.1988	1348.7328	4.6113
322	1351.0026	4.1210	1350.9690	4.2556
323	1351.4670	15.1120	1351.4456	14.7600
324	1351.9100	6.1611	1351.8773	6.4400
325	1352.0382	0.8988	1352.0234	0.9492
326	1352.2451	1.1826	1352.2000	1.5551
327	1352.6346	4.6593	1352.5864	3.8457
328	1352.9750	7.8760	1352.9186	7.5933
329	1353.1137	0.8295	1353.0110	0.5068
330	1354.0047	15.6304	1353.7532	7.9901
331	1356.0855	6.6617	1355.6372	3.6648
332	1356.2759	8.7617	1356.0036	2.3347
333	1357.2223	22.3821	1356.7576	7.6379
334	1368.8152	0.0373	1368.7830	0.6055
335	1369.0667	0.3382	1368.9898	1.3651

336	1369.9049	0.1160	1369.7394	1.6518
337	1385.7026	0.1452	1385.6626	0.0705
338	1386.0090	0.0034	1385.9765	0.0084
339	1386.4969	0.1620	1386.4688	0.0776
340	1389.0783	0.4840	1389.0368	0.1383
341	1389.4998	0.6883	1389.4615	0.3485
342	1389.6653	0.1711	1389.6520	0.0849
343	1403.7027	0.8684	1403.5912	2.0632
344	1404.8820	0.7745	1404.8443	0.8277
345	1405.7746	3.0840	1405.7356	3.2617
346	1409.4250	6.7274	1409.3870	6.3197
347	1410.9188	3.3898	1410.7969	2.4714
348	1411.1215	3.1939	1411.0060	1.0390
349	1411.9094	0.5644	1411.8438	1.4434
350	1411.9716	0.6472	1411.8663	0.3607
351	1412.3596	3.2409	1412.3105	4.0470
352	1412.6676	2.5231	1412.6267	2.4554
353	1413.0485	1.2424	1413.0214	0.9773
354	1413.5041	0.1293	1413.4798	0.0455
355	1414.8870	1.0662	1414.8693	1.0748
356	1415.6602	0.3195	1415.5958	0.1265
357	1416.2343	0.2040	1416.1847	0.0503
358	1417.4918	1.0497	1417.4301	0.6412
359	1418.7628	0.4604	1418.7504	0.5412
360	1418.9841	0.6025	1418.9540	0.3176
361	1419.3132	0.9326	1419.2916	0.8343
362	1419.7213	1.5862	1419.6486	1.0954
363	1419.7832	0.7028	1419.7533	0.7733
364	1420.3681	0.4544	1420.3117	0.4096
365	1420.5104	0.8183	1420.4770	0.5634
366	1420.6672	1.5941	1420.6413	1.3640
367	1420.9955	1.1635	1420.9708	1.2176
368	1421.0756	0.9271	1421.0212	0.8569
369	1421.2692	0.8776	1421.2222	0.7632
370	1421.5969	0.8111	1421.5758	0.5563
371	1421.8112	1.1231	1421.7823	0.8583
372	1421.9815	0.7237	1421.9283	0.6682
373	1422.1781	1.2602	1422.1401	0.5422
374	1422.3300	0.6027	1422.3016	0.5007
375	1423.1181	1.2602	1423.1117	1.1855
376	1423.5577	2.1881	1423.5319	1.7739
377	1424.0065	2.0085	1423.9694	1.6231
378	1424.6927	5.8855	1424.6791	5.8126
379	1428.4081	10.7233	1428.2989	12.3605
380	1429.1899	2.3357	1429.1268	2.1138

381	1429.5972	21.2245	1429.5427	22.0306
382	1429.9238	1.8317	1429.8931	1.8872
383	1430.6609	27.6638	1430.6375	27.5599
384	1430.9718	18.2100	1430.9346	17.6660
385	1431.2938	7.9297	1431.2425	12.5161
386	1431.3547	9.7296	1431.3129	4.5633
387	1431.6963	13.6649	1431.6786	14.0963
388	1432.2124	2.6408	1432.1302	2.8479
389	1432.8319	11.5717	1432.7598	7.3861
390	1432.9511	2.0071	1432.8851	4.5430
391	1433.2183	3.8489	1433.2089	3.4619
392	1433.6637	7.4850	1433.6194	7.2335
393	1433.8046	10.6436	1433.7016	7.4746
394	1437.1112	7.4603	1437.0790	6.7995
395	1438.2345	0.8717	1438.1101	0.6750
396	1438.4497	0.9258	1438.3392	1.0728
397	1442.3743	0.3052	1442.3620	0.2554
398	1442.9701	0.6514	1442.9492	0.4638
399	1443.3242	0.2910	1443.2956	0.1850
400	1443.6278	0.6319	1443.6224	0.5929
401	1444.1588	1.2633	1444.1472	1.2887
402	1444.1994	0.7942	1444.1935	0.7128
403	1444.9373	0.5494	1444.9104	0.4851
404	1445.2678	0.7890	1445.2541	0.5619
405	1445.7269	0.0343	1445.6963	0.0243
406	1447.0729	0.8709	1447.0236	1.1498
407	1449.2705	0.5647	1449.2150	0.3119
408	1449.9153	1.2433	1449.8588	0.6912
409	2939.4458	20.4933	2939.4435	20.5314
410	2940.5253	5.6175	2940.5246	5.5355
411	2940.5969	34.6462	2940.5959	34.7136
412	2940.8186	18.0473	2940.8185	18.0414
413	2941.6020	19.2310	2941.6005	19.2510
414	2941.7872	12.8914	2941.7845	13.0374
415	2941.8437	33.7987	2941.8429	33.7863
416	2942.0734	9.2312	2942.0708	9.1643
417	2942.1541	20.0102	2942.1515	20.0642
418	2942.3933	15.3654	2942.3928	15.3903
419	2943.4280	5.1865	2943.4268	5.1589
420	2943.7092	3.5910	2943.7077	3.5588
421	2944.5014	10.5103	2944.5013	10.4996
422	2944.7365	15.0734	2944.7364	15.0659
423	2945.2377	9.4671	2945.2375	9.4758
424	2947.0003	32.5059	2947.0000	32.4528
425	2947.0587	25.2027	2947.0583	25.2075

426	2947.3362	21.0385	2947.3361	21.0281
427	2947.4088	38.4102	2947.4085	38.4052
428	2947.5735	49.1241	2947.5732	49.1499
429	2947.7012	34.6356	2947.7010	34.6450
430	2948.3087	3.8802	2948.3087	3.8958
431	2948.3249	75.1910	2948.3249	75.2252
432	2948.4229	50.3845	2948.4228	50.5830
433	2948.4614	29.0145	2948.4613	29.1156
434	2948.5142	103.6164	2948.5139	103.4632
435	2948.5864	118.5079	2948.5862	118.6057
436	2948.6542	43.8893	2948.6541	43.8830
437	2948.7687	34.8168	2948.7685	34.7910
438	2949.0565	23.7391	2949.0563	23.7459
439	2975.2100	2.3671	2975.2098	2.3601
440	2975.2610	2.5282	2975.2608	2.5210
441	2976.1272	2.3128	2976.1270	2.3059
442	2986.8520	3.1363	2986.8520	3.1339
443	2988.0611	1.3038	2988.0611	1.3038
444	2988.3993	1.0827	2988.3993	1.0839
445	2989.1981	4.0433	2989.1981	4.0394
446	2989.3014	3.7811	2989.3013	3.7814
447	2989.5398	4.3659	2989.5398	4.3642
448	2992.0005	7.3248	2992.0005	7.3151
449	2992.1726	5.1412	2992.1726	5.1386
450	2993.6250	5.6504	2993.6249	5.6554
451	2995.2221	1.8129	2995.2220	1.8143
452	2995.9552	2.8577	2995.9551	2.8546
453	2997.6515	2.8234	2997.6515	2.8262
454	3004.3053	25.4429	3004.2936	25.7060
455	3005.3151	27.9175	3005.3060	28.1063
456	3006.4228	23.6959	3006.4154	23.7831
457	3006.5871	20.8995	3006.5775	21.0830
458	3008.3448	43.9803	3008.3444	44.0096
459	3008.4326	29.6117	3008.4325	29.6325
460	3008.7253	31.7823	3008.7249	31.7839
461	3010.1223	15.3381	3010.1161	15.4361
462	3011.0655	14.4735	3011.0607	14.5411
463	3011.5181	3.7351	3011.5169	3.7372
464	3011.7021	8.3965	3011.7020	8.4064
465	3011.7479	5.5681	3011.7472	5.5813
466	3011.9529	2.7655	3011.9516	2.7893
467	3012.2864	4.6642	3012.2862	4.6652
468	3012.4250	1.9294	3012.4233	1.9621
469	3015.1502	6.3756	3015.1493	6.3756
470	3015.3017	3.8265	3015.3013	3.8216

471	3015.3467	3.3715	3015.3465	3.3832
472	3015.8992	18.5741	3015.8988	18.5726
473	3016.0024	30.1993	3016.0011	30.0923
474	3016.4323	41.7911	3016.4312	41.6678
475	3016.7013	36.7471	3016.7009	36.8792
476	3016.8534	11.0622	3016.8528	10.9211
477	3016.9691	92.5645	3016.9684	92.5500
478	3017.9415	35.9503	3017.9413	35.9506
479	3018.3819	30.3508	3018.3815	30.3114
480	3018.9567	33.6694	3018.9563	33.6522
481	3019.7311	43.7400	3019.7303	43.8360
482	3019.9539	45.4727	3019.9527	45.3865
483	3020.4316	44.5485	3020.4307	44.4968
484	3022.2209	39.0537	3022.2177	39.1565
485	3023.6477	17.9891	3023.6475	18.0195
486	3024.2075	16.6118	3024.2069	16.6901
487	3024.3497	17.3511	3024.3494	17.3611
488	3024.9069	20.2522	3024.9032	20.2398
489	3025.5558	23.1271	3025.5513	23.2029
490	3026.8000	4.5586	3026.7997	4.5614
491	3028.1513	3.8893	3028.1512	3.8875
492	3028.8119	5.2962	3028.8118	5.2999
493	3029.5297	12.1794	3029.5294	12.1605
494	3029.7083	8.6816	3029.7080	8.6504
495	3030.3169	12.7408	3030.3168	12.7325
496	3030.6808	10.8951	3030.6778	10.9437
497	3031.1422	4.4355	3031.1398	4.4776
498	3031.3419	13.5469	3031.3401	13.4891
499	3031.7166	2.7129	3031.7146	2.4933
500	3031.7557	46.4635	3031.7540	46.7124
501	3032.2524	27.5238	3032.2514	27.5136
502	3033.3148	10.9871	3033.3134	10.9476
503	3033.7572	35.9441	3033.7540	36.0439
504	3034.0452	59.9952	3034.0425	60.0667
505	3035.3911	51.3145	3035.3908	51.3775
506	3035.5057	25.2425	3035.5049	25.1251
507	3036.0001	35.6925	3035.9998	35.6628
508	3037.2618	3.5257	3037.2611	3.5208
509	3037.4794	1.8790	3037.4780	1.8813
510	3037.8835	9.4925	3037.8827	9.4930
511	3040.9615	9.8047	3040.9604	9.7704
512	3041.6192	9.8141	3041.6181	9.7692
513	3046.7523	13.4392	3046.7515	13.3986

Table S10. ViPA breakdown of modes of **1-Y$_D$** in the basis of modes of **1-Y**. Only contributions > 5% are listed.

**1-Y$_D$**		**1-Y**		
Mode	Energy (cm^{-1})	Contribution (%)*	Mode	Energy (cm^{-1})
132	441.8459	98.202	156	621.3487
158	697.2404	18.740<br>9.932<br>63.853	158<br>159<br>218	705.8797<br>706.3220<br>982.3701
159	699.5489	37.348<br>46.203	157<br>219	705.4851<br>978.0069
206	922.0438	5.001<br>11.547<br>49.019<br>9.842	294<br>297<br>299<br>302	1298.3446<br>1301.4234<br>1302.5237<br>1315.9010
207	926.6511	60.519<br>18.379	300<br>303	1303.7812<br>1316.7953

For calculation of the UV-Vis spectra and hyperfine coupling of **1-Y** (and **1-Y$_D$**), and the exchange coupling of **1-Gd**, the positions of the bridging hydride atoms were optimized from the respective X-Ray crystallographic structures using density-functional theory with ORCA v5.0.0.[26-28] In the case of **1**-Gd, the Gd atoms were replaced with closed-shell Y to facilitate convergence of the SCF. The PBE0 density-functional was used in conjunction with the RI-COSX approximation, where 28-electron def2-ECP were employed for I and Y, and the def2-TZVP valence basis set was used for all atoms.[21, 29-32]

For determination of the UV-Vis spectrum of **1-Y**, the electronic structure was calculated using CASSCF methods in OpenMolcas v21.06, where the two-electron integrals were decomposed with a Cholesky decomposition (10^{-8} threshold), and the basis sets were from the ANO-RCC library with VDZP quality for Y, I, H(hydride) and C(Cp), and with MB quality for all other atoms.[33-35] We employed the second order DKH relativistic decoupling.[36] State-averaged CASSCF (SA-CASSCF) calculations were performed for the 16 lowest doublet states in an active space of 1 electron in 16 orbitals, comprising orbitals with significant Y(4d) character. The ground state SOMO is a σ-like bonding orbital comprised of the d_{z^2} orbitals of the Y ions, while the space of Y(4d) excited states comprise various combinations with π-like and δ-like character (Table S11). Corrections for dynamic electron correlation were added using multiconfigurational pair-density-functional theory (MCPDFT) using the tPBE functional.[37] The UV-Vis spectrum was calculated on the basis of the SA-CASSCF transition intensities in the velocity gauge and the MCPDFT energies, and is in excellent agreement with the experiment (Figure S49). The feature at ca. 13,200 cm^{-1} is the σ to σ* transition (state 1 to 4/5), and the feature at ca. 33,300 cm^{-1} is a σ to π transition (state 1 to 15).

For determination of the hyperfine coupling and spin density in **1-Y** and **1-Y$_D$**, we employed ORCA v5.0.0 using the PBE density-functional with the ZORA relativistic Hamiltonian, the RI approximation, the ZORA-def2-TZVP basis set for all C and H atoms, and the old-ZORA-TZVP

basis set for Y and I. Isotropic and anisotropic hyperfine couplings were using the picture change corrections (Table S12), giving $A_\perp(^{89}Y) = 13(2)$ MHz, $A_\parallel(^{89}Y) = -0.43(8)$ MHz, $A_\perp(^{127}Y) = -42(1)$ MHz, $A_\parallel(^{127}Y) = -0.17$ and -2.53 MHz, $A_\perp(^{2}H) = -2.1(3)$ MHz, $A_\parallel(^{2}H) = -0.48(1)$ MHz.

For determination of the exchange coupling in **1-Gd**, we employed broken-symmetry DFT and SA-CASSCF-MCPDFT calculations. Broken-symmetry DFT calculations were performed with the Gaussian 09 rev. D package.[15] The B3LYP density-functional was used[38-40] in conjunction with the 6-31G* basis set for carbon and hydrogen atoms,[41] the 46 core electron Stuttgart-Dresden ECP and the corresponding double-zeta valence basis set for I,[42-44] while the 46 core electron Cundari Stevens Double Zeta ECP and the corresponding valence basis set was employed for Gd.[45] Calculations were performed for the high-spin, and four broken-symmetry solutions (Table S14 and Figure S51), leading to the exchange coupling values of $J_{4f-\sigma} = 176$ cm^{-1} with $J_{Gd-Gd} = -2.2$ cm^{-1} obtained using the Noodleman method.[46] CASSCF-MCPDFT calculations were performed with OpenMolcas v21.06,[33] using a Cholesky decomposition for the two-electron integrals (10^{-8} threshold), second order DKH relativistic decoupling,[36] and basis sets from the ANO-RCC library with VDZP quality for Gd, I, H(hydride) and C(Cp), and with MB quality for all other atoms.[34-35] The Gd atoms were first replaced with Lu (ANO-RCC-VDZP basis) and the σ-like SOMO was obtained using a CAS(1,1)SCF calculation (Figure S50). The inactive orbitals were localized using the Pipek-Mezey method,[47] and the 4f orbitals for each Ln site were identified. CASSCF calculations were then performed for each Gd site separately, where the other two Gd sites remained defined as Lu, considering one $S = 4$ and one $S = 3$ root for an active space of 8 electrons in 8 orbitals, comprising the Gd 4f orbitals and the σ-like SOMO obtained from the Lu_3 calculation which was not allowed to relax in the SCF procedure. Corrections for dynamic electron correlation were added using multiconfigurational pair-density-functional theory (MCPDFT) using the tPBE functional.[37] The energies show that $J_{4f-\sigma}$ is ferromagnetic and the average values for CASSCF and MCPDFT are $J_{4f-\sigma} = 333$ cm^{-1} and 219 cm^{-1}, respectively (Table S15).

Table S11. SA-CASSCF-MCPDFT energies (in cm^{-1}) and natural orbitals for the low-lying excited states in **1-Y**.

State	CASSCF Energy	CASSCF-MCPDFT Energy	Orbital
1	0	0	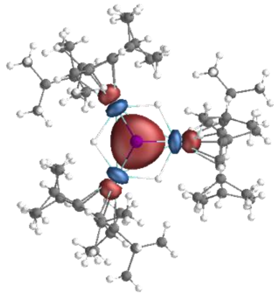

2	12183	9473	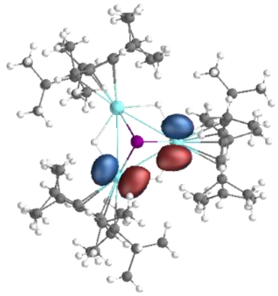
3	12728	9970	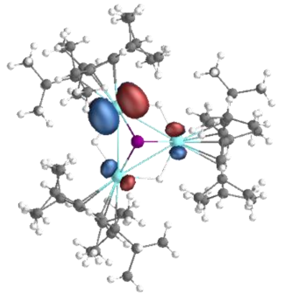
4	17551	13201	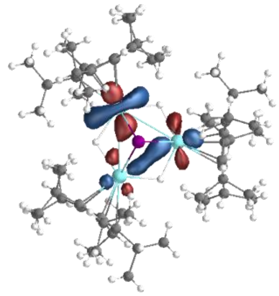
5	17661	13228	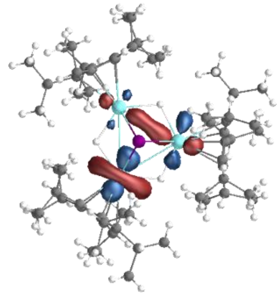
6	20312	16116	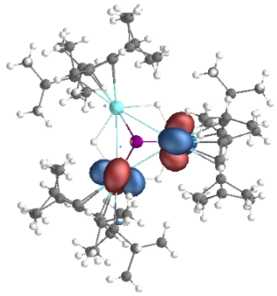

7	20643	15943	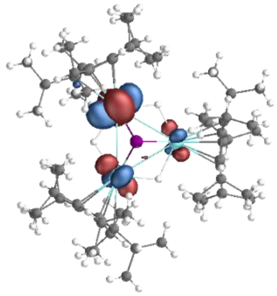
8	21411	12668	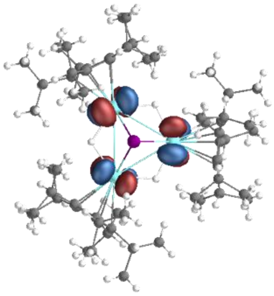
9	24525	18901	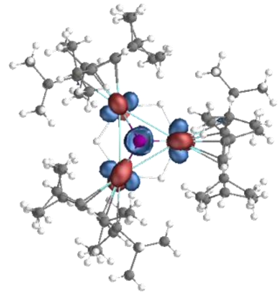
10	32036	26420	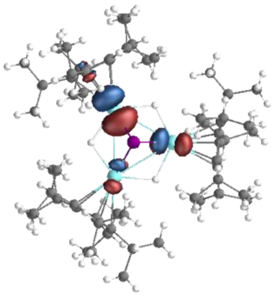
11	32182	26531	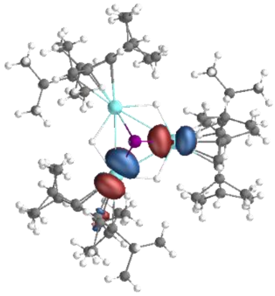

12	33273	26251	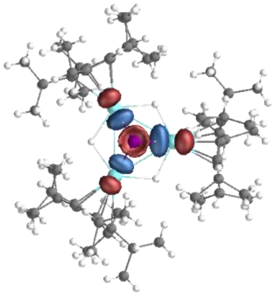
13	35685	29269	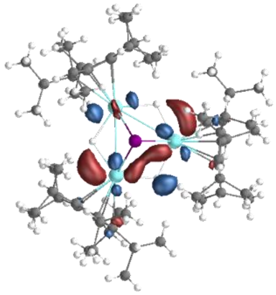
14	36405	29988	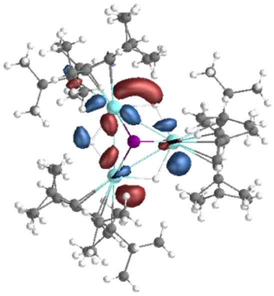
15	39349	33267	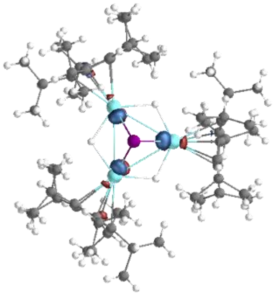
16	43689	31964	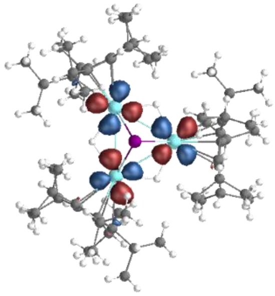

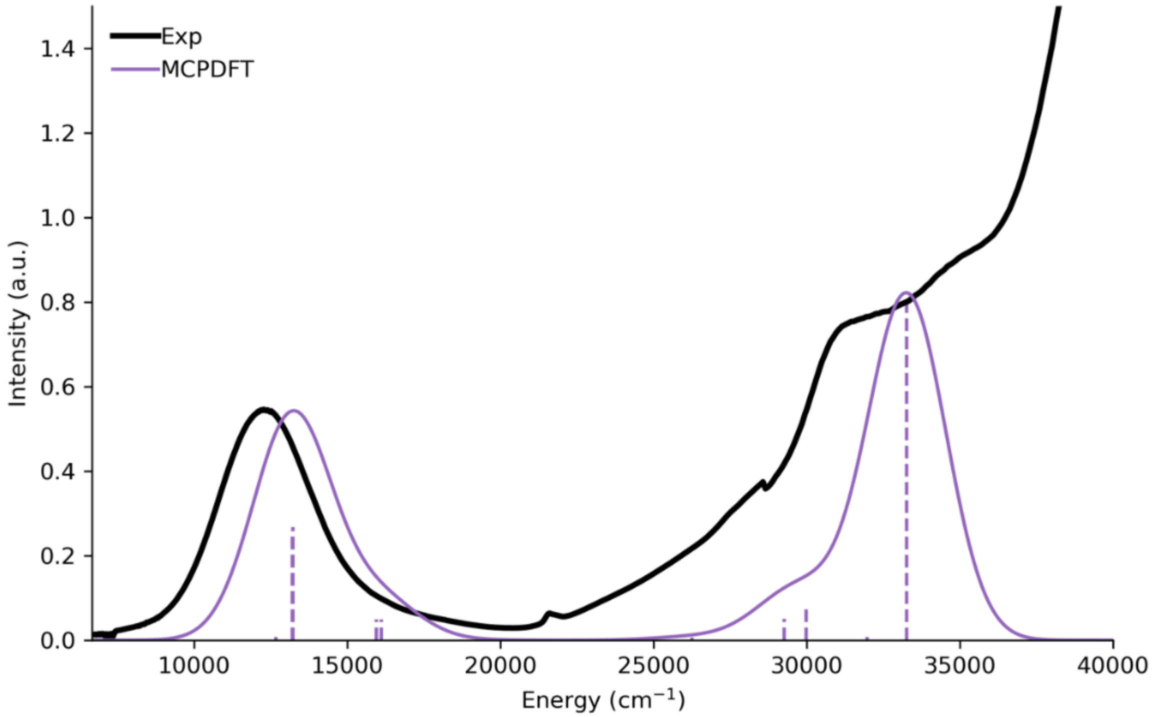

Figure S46. Comparison of experimental (black trace) and calculated (purple trace) UV-Vis spectra of **1-Y**. The calculated spectrum was determined from SA-CASSCF-MCPDFT methods as described above.

Table S12. DFT-calculated hyperfine parameters in **1-Y** and **1-Y$_D$**. The calculated *g* tensor components are g_1 = 1.91, g_2 = 1.98, g_3 = 2.02.

**Nucleus**	**A_1 (MHz) ($A_{\|\|}$)**	**A_2 (MHz)**	**A_3 (MHz)**	**Avg of A_2 and A_3 ($A_\perp$)**
^{89}Y1	−0.36	11.59	14.89	
^{89}Y2	−0.52	11.04	13.83	
^{89}Y3	−0.41	11.45	14.70	
**Avg ^{89}Y**	**−0.43**	**11.36**	**14.47**	**12.92**
^{127}I1	−0.17	−42.27	−43.89	
^{127}I2	−2.53	−40.60	−41.55	
**Avg ^{127}I**	**−1.35**	**−41.44**	**−42.72**	**−42.08**
^{1}H1/^{2}H1	−3.05/−0.48	−12.11/−1.88	−15.45/−2.40	
^{1}H2/^{2}H2	−3.12/−0.49	−12.18/−1.90	−15.53/−2.42	
^{1}H3/^{2}H3	−3.02/−0.47	−11.73/−1.82	−14.82/−2.31	
**Avg ^{2}H**	**−0.48**	**−1.87**	**−2.38**	**−2.13**

Table S13. DFT-calculated spin density in **1-Y**.

Atom	s (%)	p (%)	d (%)	Total (%)
Y1	0.7	2.3	28.4	92.0
Y2	0.7	2.2	27.0	
Y3	0.7	2.3	27.8	
I1	0.0	−0.4	2.3	3.6
I2	0.0	−0.6	2.3	
H1	−0.5	0.2	-	−1.0
[1]H2	−0.5	0.2	-	
H3	−0.5	0.1	-	

Table S14. Broken-symmetry DFT results for exchange coupling in **1-Gd**.

State/Site	Spin configuration Gd1 - Gd2 - Gd3 - σ	Relative Energy (cm^{-1})
**High-spin**	↑ - ↑ - ↑ - ↑	0
**BS1**	↓ - ↑ - ↑ - ↑	1017
**BS2**	↑ - ↓ - ↑ - ↑	1044
**BS3**	↑ - ↑ - ↓ - ↑	998
**BS4**	↑ - ↑ - ↑ - ↓	3698

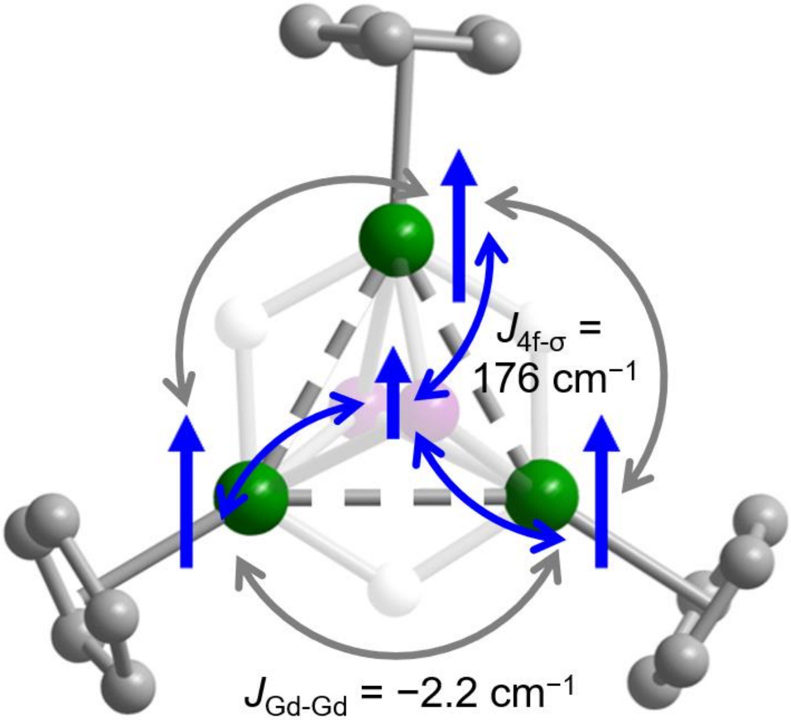

Figure S47. Illustration of the spin-spin coupling topology of the four spin centers in **1-Gd**. The spin-spin exchange coupling values were obtained using the Noodleman method.[46] The calculated $J_{4f-\sigma}$ value agrees well with the value of $J_{4f-\sigma}$ = 168(1) cm^{-1} determined from experimental data as discussed in the main text. A small J_{Gd-Gd} = −2.2 cm^{-1} was also determined using DFT.

Table S15. CASSCF and CASSCF-MCPDFT results for exchange coupling in **1-Gd**.

**State/Site**	**CASSCF**			**CASSCF-MCPDFT**		
	**Gd1**	**Gd2**	**Gd3**	**Gd1**	**Gd2**	**Gd3**
$\boldsymbol{E(S=3)-E(S=4)}$ **(cm^{-1})**	2510	2741	2736	1656	1807	1797
$\boldsymbol{J_{4f-\sigma}}$ **(cm^{-1})**	314	343	342	207	226	225

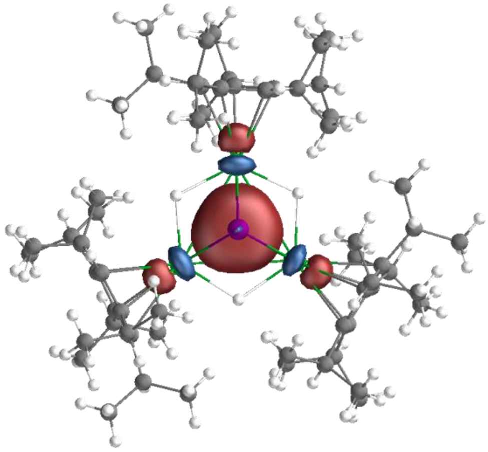

Figure S48. σ bonding SOMO of **1-Gd** as determined from CAS(1,1)SCF calculations where Gd has been replaced with Lu.

9. References

1 Dezember, T. and Sitzmann, H. *Z. Naturforsch B.* **1997**, *52*, 911.

2 Gould, C. A.; McClain, K. R.; Reta, D.; Kragskow, J. G. C.; Marchiori, D. A.; Lachman, E.; Choi, E.-S.; Analytis, J. G.; Britt, R. D.; Chilton, N. F.; Harvey, B. G.; Long, J. R. Ultrahard Magnetism from Mixed-Valence Dilanthanide Complexes with Metal-Metal Bonding. *Science* **2022**, *375*, 198–202.

3 Connelly, S. J.; Kaminsky, W.; Heinekey, D. M. Structure and Solution Reactivity of (Triethylsilylium)triethylsilane Cations. *Organometallics* **2013**, *32*, 7478.

4 (a) Sheldrick, G. M. SHELXT, University of Göttingen, Germany, 2015 (b) Sheldrick, G. M. SHELXL, University of Göttingen, Germany, 2015.

5 Dolomanov, O. V.; Bourhis, L. J.; Gildea, R. J.; Howard, J. A. K.; Puschmann, H. *J. Appl. Cryst.* **2009**, *42*, 339–341.

6 Chilton, N. F.; Anderson, R. P.; Turner, L. D.; Soncini, A.; Murray, K. S. PHI: a powerful new program for the analysis of anisotropic monomeric and exchange-coupled polynuclear d- and f-block complexes. *J. Comput. Chem.* **2013**, *34*, 1164–1175.

7 Oyala, P. H.; Ravichandran, K. R.; Funk, M. A.; Stucky, P. A., Stich, T. A.; Drennan, C. L.; Britt, R. D.; Stubbe, J. Biophysical Characterization of Fluorotyrosine Probes SiteSpecifically Incorporated into Enzymes: E. coli Ribonucleotide Reductase as an Example. *J. Am. Chem. Soc.* **2016**, *138*, 7951–7964

8 Cox, N.; Lubitz, W.; Savitsky, A. W-band ELDOR-detected NMR (EDNMR) spectroscopy as a versatile technique for the characterisation of transition metal–ligand interactions. *Mol. Phys.* **2013**, *111*, 2788–2808
9 Stoll, S.; Schweiger, A. EasySpin, a comprehensive software package for spectral simulation and analysis in EPR *J. Magn. Reson.* **2006**, *178*, 42–55
10 Cox, N.; Nalepa, A.; Lubitz, W.; Savitsky, A. ELDOR-Detected NMR: A General and Robust Method for Electron-Nuclear Hyperfine Spectroscopy? *J. Magn. Reson.* **2017**, *280*, 63–78.
11 Goldfarb, D. ELDOR-detected NMR. *eMagRes* **2017**, *6*, 101–114.
12 Cox, N.; Retegan, M.; Neese, F.; Pantazis, D. A.; Boussac, A.; Lubitz, W. Electronic Structure of the Oxygen-Evolving Complex in Photosystem II Prior to O-O Bond Formation. *Science* **2014**, 345, 804–808.
13 Bennati, M. EPR Interactions – Hyperfine Couplings. *eMagRes* **2017**, *6*, 271–282.
14 Morton, J. R.; Preston, K. F. Atomic Parameters for Paramagnetic Resonance Data. *J. Magn. Reson.* **1978**, *30*, 577–582.
15 Frisch, M. J.; Trucks, G. W.; Schlegel, H. B.; Scuseria, G. E.; Robb, M. A.; Cheeseman, J. R.; Scalmani, G.; Barone, V.; Mennucci, B.; Petersson, G. A.; Nakatsuji, H.; Caricato, M.; Li, X.; Hratchian, H. P.; Izmaylov, A. F.; Bloino, J.; Zheng, G.; Sonnenberg, J. L.; Hada, M.; Ehara, M.; Toyota, K.; Fukuda, R.; Hasegawa, J.; Ishida, M.; Nakajima, T.; Honda, Y.; Kitao, O.; Nakai, H.; Vreven, T.; Montgomery, J. A., Jr.; Peralta, J. E.; Ogliaro, F.; Bearpark, M.; Heyd, J. J.; Brothers, E.; Kudin, K. N.; Staroverov, V. N.; Kobayashi, R.; Normand, J.; Raghavachari, K.; Rendell, A.; Burant, J. C.; Iyengar, S. S.; Tomasi, J.; Cossi, M.; Rega, N.; Millam, J. M.; Klene, M.; Knox, J. E.; Cross, J. B.; Bakken, V.; Adamo, C.; Jaramillo, J.; Gomperts, R.; Stratmann, R. E.; Yazyev, O.; Austin, A. J.; Cammi, R.; Pomelli, C.; Ochterski, J. W.; Martin, R. L.; Morokuma, K.; Zakrzewski, V. G.; Voth, G. A.; Salvador, P.; Dannenberg, J. J.; Dapprich, S.; Daniels, A. D.; Farkas, Ö.; Foresman, J. B.; Ortiz, J. V.; Cioslowski, J.; Fox, D. J. Gaussian 09, Revision D.01, Wallingford CT 2013.
16 Perdew, J. P.; Burke, K.; and Ernzerhof, M. *Phys. Rev. Lett.*, **1997**, *78*, 1396–1396.
17 Perdew, J. P.; Burke, K.; and Ernzerhof, M. *Phys. Rev. Lett*., **1996**, *77*, 3865–3868.
18 Grimme, S. Wiley Interdiscip. *Rev. Comput. Mol. Sci*., **2011**, *1*, 211–228.
19 Grimme, S. *J. Comput. Chem.*, **2004**, *25*, 1463–1473.
20 Grimme, S. *J. Comput. Chem.*, **2006**, *27*, 1787–1799.
21 Dunning, T. H. *J. Chem. Phys.*, **1989**, *90*, 1007–1023.
22 Peterson, K. A.; Figgen, D.; Goll, E.; Stoll, H.; Dolg, M. *J. Chem. Phys.*, **2003**, *119*, 11113–11123.
23 Dolg, M.; Stoll, H.; Preuss, H.; Pitzer, R. M. *J. Phys. Chem.*, **1993**, *97*, 5852–5859.
24 Andrae, D.; Häußermann, U.; Dolg, M.; Stoll, H.; Preuß, H. *Theor. Chim. Acta*, **1990**, *77*, 123–141.
25 Grafton, A. K.; Wheeler, R. A. *J. Comput. Chem.*, **1998**, *19*, 1663–1674.
26 Neese, F. The ORCA program system, Wiley Interdiscip. *Rev.: Comput. Mol. Sci.*, **2012**, *2*, 73–78.
27 Neese, F. Software update: the ORCA program system, version 4.0, Wiley Interdiscip. *Rev.: Comput. Mol. Sci.*, **2017**, *8*, e1327.
28 Neese, F.; Wennmohs, F.; Becker, U.; Riplinger, C. The ORCA quantum chemistry program package, *J. Chem. Phys.*, **2020**, *152*, 224108.
29 Weigend, F.; Ahlrichs, R. *Phys. Chem. Chem. Phys.* **2005**, *7*, 3297.

30 Andrae, D.; Haeussermann, U.; Dolg, M.; Stoll, H.; Preuss, H. *Theor. Chim. Acta,* **1990**, *77*, 123-141

31 Weigend, F. *Phys. Chem. Chem. Phys.*, **2006**, *8*, 1057.

32 Adamo, C.; Barone, V. *J. Chem. Phys.*, **1999**, *110*, 6158.

33 Fdez. Galván, I.; Vacher, M.; Alavi, A.; Angeli, C.; Aquilante, F.; Autschbach, J.; Bao, J. J.; Bokarev, S. I.; Bogdanov, N. A.; Carlson, R. K.; Chibotaru, L. F.; Creutzberg, J.; Dattani, N.; Delcey, M. G.; Dong, S. S.; Dreuw, A.; Freitag, L.; Frutos, L. M.; Gagliardi, L.; Gendron, F.; Giussani, A.; González, L.; Grell, G.; Guo, M.; Hoyer, C. E.; Johansson, M.; Keller, S.; Knecht, S.; Kovačević, G.; Källman, E.; Li Manni, G.; Lundberg, M.; Ma, Y.; Mai, S.; Malhado, J. P.; Malmqvist, P. Å.; Marquetand, P.; Mewes, S. A.; Norell, J.; Olivucci, M.; Oppel, M.; Phung, Q. M.; Pierloot, K.; Plasser, F.; Reiher, M.; Sand, A. M.; Schapiro, I.; Sharma, P.; Stein, C. J.; Sørensen, L. K.; Truhlar, D. G.; Ugandi, M.; Ungur, L.; Valentini, A.; Vancoillie, S.; Veryazov, V.; Weser, O.; Wesołowski, T. A.; Widmark, P.-O.; Wouters, S.; Zech, A.; Zobel, J. P.; Lindh, R. OpenMolcas: From Source Code to Insight. *J. Chem. Theory Comput.* **2019**, *15*, 5925–5964.

34 Roos, B. O.; Lindh, R.; Malmqvist, P.A.; Veryazov, V.; Widmark, P.O. *J. Phys. Chem. A*, **2005**, *109*, 6575–6579.

35 Roos, B. O.; Lindh, R.; Malmqvist, P.A.; Veryazov, V.; Widmark, P.O. *J. Phys. Chem. A*, **2004**, *108*, 2851–2858.

36 Reiher, M. *Theor Chem Acc*, **2006**, *116*, 241–252.

37 Manni, G. L.; Carlson, R. K.; Luo, S.; Ma, D.; Olsen, J.; Truhlar, D. G.; Gagliardi, L. *J. Chem. Theory Comput.* **2014**, *10*, 3669–3680.

38 Becke, A.D. *J.Chem.Phys.* **1993**, *98*, 5648-5652.

39 Lee, C.; Yang, W.; Parr, R.G. *Phys. Rev. B*, **1998**, *37*, 785-789.

40 Stephens, P.J.; Devlin, F.J.; Chabalowski, C.F.; Frisch, M.J. *J.Phys.Chem*. **1994**, *98*, 11623-11627.

41 Ditchfield, R; Hehre, W.J; Pople, J. A. Self-Consistent Molecular-Orbital Methods. IX. An Extended Gaussian-Type Basis for Molecular-Orbital Studies of Organic Molecules. *J. Chem. Phys*. **1971**, *54*, 724–728.

42 Chan, W.T.; Fournier, R. Binding of ammonia to small copper and silver clusters. *Chem. Phys. Lett*. **1999**, *315*, 257–265.

43 Valdés, Á.; Prosmiti, R.; Villarreal, P.; Delgado-Barrio, G. $HeBr_2$ complex: Ground-state potential and vibrational dynamics from ab initio calculations. *Mol. Phys.* **2004**, *102*, 2277–2283.

44 Lei, M.; Wang, N.; Zhu, L.H.; Tang, H.Q. Peculiar and rapid photocatalytic degradation of tetrabromodiphenyl ethers over Ag/TiO_2 induced by interaction between silver nanoparticles and bromine atoms in the target. *Chemosphere* **2016**, *150*, 536–544.

45 Cundari, T. R.; Stevens, W. J. *J. Chem. Phys.*, **1993**, *98*, 5555–5565.

46 Noodleman, L.; Case, D. A.; Aizman, A. Broken symmetry analysis of spin coupling in iron-sulfur clusters *J. Am. Chem. Soc.* **1988**, *110*, 1001–1005

47 Pipek, J.; Mezey, P. G. A fast intrinsic localization procedure applicable for ab initio and semiempirical linear combination of atomic orbital wave functions. *J. Chem. Phys*. **1989**, 90, 4916.
